# A machine learning-derived aging index for risk stratification and mortality prediction in cardiovascular-kidney-metabolic syndrome: A retrospective cohort study

**DOI:** 10.1371/journal.pmed.1005078

**Published:** 2026-10-06

**Authors:** Zhengyang Zhu, Kejun Ren, Dong Wang, Yong Lv, Hua Jin, Lei Zhang, Yiping Wang

**Affiliations:** First Affiliated Hospital, Anhui University of Chinese Medicine, Hefei, Anhui Province, China; University Medical Center Groningen, KINGDOM OF THE NETHERLANDS

## Abstract

**Background:**

The cardiovascular‑kidney‑metabolic (CKM) syndrome shows substantial heterogeneity in progression, yet existing biological age indices are not tailored to CKM pathways. We developed an aging index (CKMAI) using machine learning and evaluated its predictive performance for mortality and high‑risk CKM status.

**Methods and findings:**

We conducted a retrospective cohort study of 6,896 adults from the National Health and Nutrition Examination Survey (NHANES) 2005–2018, weighted for the US adult population. A two‑stage machine learning framework evaluated over 100 candidate survival models; the optimal model was selected via Pareto front optimization. Predictive performance was assessed using time‑dependent area under the curve (AUC), net reclassification improvement (NRI), integrated discrimination improvement (IDI), calibration, and decision curve analysis (DCA). Nonlinear relationships and threshold effects were examined with restricted cubic splines and two‑piecewise regression. Additive interactions were quantified by relative excess risk due to interaction (RERI) and attributable proportion (AP). Unsupervised clustering identified aging‑metabolic phenotypes. Weighted bootstrap mediation analysis assessed the mediating role of depression.

The Pareto‑optimal simple Cox model achieved a mean C‑index of 0.893. CKMAI consistently outperformed PhenoAge, KDM, and the cardiometabolic index (CMI) across all outcomes. For all‑cause mortality, CKMAI’s time‑dependent AUCs were 0.893 (95% CI [0.851, 0.925]) at 3 years, 0.907 (95% CI [0.884, 0.927]) at 5 years, and 0.890 (95% CI [0.868, 0.910]) at 10 years; for cardiovascular mortality, 0.904 (95% CI [0.840, 0.943]), 0.937 (95% CI [0.892, 0.960]), and 0.937 (95% CI [0.900, 0.959]); for high‑risk CKM status, 0.790 (95% CI [0.765, 0.816]). NRI and IDI were significant at all time points (*P* < 0.05 for all), calibration was good (Greenwood‑Nam‑D’Agostino test *P* > 0.05), and DCA confirmed superior net benefit. Nonlinear associations were found for all three outcomes. Threshold analysis identified inflection points at CKMAI 61.587 (all‑cause mortality), 60.732 (cardiovascular mortality), and 34.092 (high‑risk CKM status), with steeper risk increases below each threshold. CKMAI exhibited super‑additive interactions with PhenoAge (RERI 15.06, 95% CI [8.14, 38.44]). Clustering revealed six distinct aging‑metabolic phenotypes; the frail elderly cluster had the highest mortality risk (hazard ratio [HR] 11.74, 95% CI [7.85, 17.57]). Depression partially mediated the CKMAI‑outcome associations (proportions mediated: 5.6% [95% CI 2.3%–9.0%] for all‑cause mortality, 8.2% [95% CI 2.8%–22.0%] for cardiovascular mortality, 11.1% [95% CI 6.7%–24.0%] for high‑risk CKM status). Findings were robust across prespecified subgroups and multiple sensitivity analyses. A key limitation is the lack of validation in geographically independent cohorts with complete mortality follow-up; however, temporal validation within NHANES and preliminary external validation in a hospital based Chinese cohort (*n* = 261) supported the generalizability of the CKMAI for identifying high risk CKM status.

**Conclusions:**

CKMAI is a valid, CKM‑specific aging index that outperforms universal biological age measures in predicting mortality and identifying high‑risk CKM status. Its super‑additive interactions with PhenoAge and the partial mediation by depression offer new mechanistic insights into CKM heterogeneity. By integrating a diverse set of clinical laboratory measures, nutritional assessments, lifestyle factors, and social determinants of health, CKMAI provides a practical, implementable tool for risk stratification and targeted prevention in CKM syndrome.

## Author summary

**Why was this study done?:**

- CKM syndrome is a complex condition involving the heart, kidneys, and metabolism, but it progresses very differently from person to person, making it difficult to predict who will develop serious complications.
- Existing measures of biological aging were not specifically designed for CKM syndrome, and it was unclear whether a tool tailored to this condition could better identify high-risk individuals.
- Depression is common in people with CKM syndrome, but its role in accelerating disease progression has not been well studied.

**What did the researchers do and find?:**

- Using data from nearly 7,000 US adults and machine learning techniques, the researchers developed a new aging index (CKMAI) specifically for CKM syndrome based on 18 routine blood tests and clinical measures.
- The CKMAI outperformed existing aging indices in predicting death from any cause, death from cardiovascular disease, and identifying individuals at high risk of CKM progression.
- The index was validated in a separate hospital cohort of 261 Chinese patients and across different time periods within the main dataset, confirming its reliability.
- Depression was found to partially explain the link between accelerated aging and CKM progression, suggesting that addressing mental health could be a target for intervention.

**What do these findings mean?:**

- The CKMAI provides a practical, low-cost tool that could help doctors identify patients with CKM syndrome who need more intensive monitoring or earlier treatment.
- The results suggest that combining CKM-specific aging measures with depression screening could improve risk assessment and guide personalized care.
- To facilitate clinical use, the researchers developed a free online calculator (Shiny app) and an open-source R package that allow clinicians and researchers to easily compute the CKMAI.
- The main limitations are that the primary analysis relied on a single US dataset and the hospital validation cohort was relatively small and lacked long-term follow-up for deaths, so further validation in larger, more diverse populations is needed.

## Abstract 3

In this cohort study, Zhengyang Zhu and colleagues developed a machine learning-derived cardiovascular-kidney-metabolic aging index that outperformed established biological age measures in identifying high-risk CKM profiles and predicting mortality, while revealing distinct aging-metabolic phenotypes and associations with depression that may inform risk stratification and prevention efforts.

## Introduction

CKM syndrome, introduced by the American Heart Association in 2023, represents a paradigm shift in understanding the intertwined pathophysiology of obesity, diabetes, chronic kidney disease (CKD), and cardiovascular disease (CVD) [1,2]. The CKM staging system (stages 0–4) provides a structured framework for risk stratification, yet disease progression exhibits substantial interindividual heterogeneity: some individuals remain stable at early stages for years, while others rapidly advance to subclinical or clinical cardiovascular disease [3]. The determinants of this heterogeneity remain poorly understood, limiting the development of precision prevention strategies [4,5].

Biological age indices have emerged as powerful tools to capture interindividual variation in aging rates. PhenoAge and KDM biological age, derived from clinical biomarkers, have demonstrated robust associations with all-cause and cardiovascular mortality [6,7]. However, these are universal measures not specifically optimized for CKM-related pathways, despite growing evidence that inflammation, oxidative stress, and insulin resistance—central to CKM progression—are also key drivers of biological aging [8,9]. We therefore conceptualized the CKMAI as an aging index specifically tailored to the CKM pathway—not as a replacement for universal biological age measures, but as a complementary tool that captures the physiological decline occurring across the metabolic, renal, and inflammatory systems that are central to CKM syndrome. While CKMAI is specialized for CKM risk, the biomarkers it incorporates reflect well established hallmarks of biological aging, including chronic inflammation, oxidative stress, and organ function decline. Thus, CKMAI quantifies the biological wear and tear accumulating along the CKM continuum, rather than merely serving as a disease specific score. This raises the possibility that a CKM-specific aging index, integrating biomarkers across the metabolic, kidney, and cardiovascular domains, could outperform universal measures in predicting CKM-relevant outcomes [10].

Depression commonly co-occurs with CKM syndrome and has been linked to accelerated biological aging [11]. Depressive symptoms are more prevalent in advanced CKM stages [12], and depression independently predicts cardiovascular events and mortality in populations with metabolic and kidney diseases [13,14]. Emerging evidence suggests that systemic inflammation partially mediates this association [15,16]. Yet the role of depression as a potential mediator between CKM-specific aging and mortality has not been systematically examined, representing a critical gap in understanding the psychological-biological interface of CKM progression.

In this study, we developed a CKMAI using machine learning, integrating biomarkers across metabolic, kidney, aging, inflammatory-nutritional, and psychosocial domains. We evaluated its predictive performance for all-cause and cardiovascular mortality and high-risk CKM status compared with PhenoAge, KDM, and a traditional metabolic index (CMI). We also explored additive interactions with universal biological age measures, identified aging-metabolic phenotypes via clustering, and quantified the mediating role of depression in the CKMAI-mortality association. To assess the generalizability of the index, we performed multiple complementary validation strategies: temporal validation within NHANES (training on 2005–2010 and testing on 2011–2018), fixed weight validation across nonoverlapping cycles, and external validation in an independent hospital cohort (*n* = 261). By leveraging nationally representative data from NHANES, this study aimed to provide a practical tool for risk stratification in CKM syndrome and to offer insights into the heterogeneous mechanisms driving its progression.

## Methods

### Study design and data sources

This retrospective cohort study used data from NHANES conducted between 2005 and 2018. This study is reported according to the Strengthening the Reporting of Observational Studies in Epidemiology (STROBE) guideline for cohort studies (S1 Checklist), as this study also involves the development and validation of a risk prediction model using machine learning, we additionally followed the Transparent Reporting of a multivariable prediction model for Individual Prognosis Or Diagnosis – Artificial Intelligence (TRIPOD‑AI) guideline (S2 Checklist). NHANES is a continuous, nationally representative cross‑sectional survey of the noninstitutionalized civilian US population, administered by the National Center for Health Statistics (NCHS) of the Centers for Disease Control and Prevention (CDC) [17,18]. The survey employs a complex, multistage probability sampling design to ensure national representativeness. Data collection includes standardized in‑home interviews, physical examinations at mobile examination centers, and comprehensive laboratory assessments following rigorous quality control protocols [19]. Mortality outcomes were ascertained through probabilistic linkage to the National Death Index (NDI), with follow‑up available through December 31, 2019 [20]. The National Health and Nutrition Examination Survey (NHANES) protocol was approved by the National Center for Health Statistics (NCHS) Research Ethics Review Board (Protocol #2011-17). All NHANES participants provided written informed consent. The present study is a secondary analysis of de-identified, publicly available NHANES data and was exempt from additional institutional review board approval [21]. For the hospital-based validation cohort, the study was conducted in accordance with the Declaration of Helsinki. The requirement for formal ethics approval and written informed consent was waived by the Medical Ethics Committee of the First Affiliated Hospital of Anhui University of Chinese Medicine for this retrospective study utilizing fully anonymized medical records. The study was determined to be exempt from full ethics review based on its retrospective, noninterventional design, the use of de-identified data, and the minimal risk posed to participants, in accordance with Chinese regulations for research using anonymized clinical data. All patient data were anonymized prior to analysis, and patient privacy was protected throughout the study. The sample size was determined by the availability of complete data in NHANES 2005–2018; post‑hoc power analysis indicated that the study had >80% power to detect hazard ratios (HRs) of 1.05 or greater for all‑cause mortality.

### Study population

A total of 70,190 participants of all ages were initially identified from the NHANES 2005–2018 cycles. Participants were sequentially excluded according to prespecified criteria. First, individuals younger than 18 years (*n* = 28,047) were removed to focus the analysis on the adult population. Next, participants with missing data for CKM stage classification (*n* = 14,747) were excluded. Subsequently, participants with incomplete information on the primary outcome or core exposure variables (*n* = 17,201) were removed. Finally, participants with missing data for any covariate included in the multivariable models (*n* = 3,299) were excluded. After applying these exclusion criteria, the final analytical cohort comprised 6,896 participants, when weighted to account for the complex survey design, the analytical cohort was representative of the noninstitutionalized US adult population (2005–2018). Participants were randomly divided into a training set (70%, *n* = 4,827) and a testing set (30%, *n* = 2,069) using stratified sampling to preserve the distribution of CKM stages and survey years. For temporal validation, the same sequential exclusion criteria were applied to extract two independent time windows: an early period (2005–2010, *n* = 3,900) and a late period (2011–2018, *n* = 2,996). The early period was used as a secondary training set for retraining the entire modeling pipeline, and the late period served as a temporally independent test set. The detailed participant selection process is illustrated in Fig 1. Because participants were enrolled across different NHANES cycles (2005–2018), follow-up durations varied substantially, ranging from 1 to 14 years. To ensure comparability and minimize the impact of differential follow-up, we used time dependent AUC at fixed time points (3, 5, and 10 years) as the primary discrimination metric for mortality outcomes. This approach standardizes performance evaluation at specific horizons rather than relying on variable individual follow-up lengths. Additionally, CKM stages were assessed at baseline using clinical and laboratory data collected during the index NHANES examination visit, and all outcomes (mortality and CKM high risk status) were measured from baseline forward. This is consistent with the American Heart Association CKM staging framework and the study objectives of baseline risk stratification.

**Fig 1:**
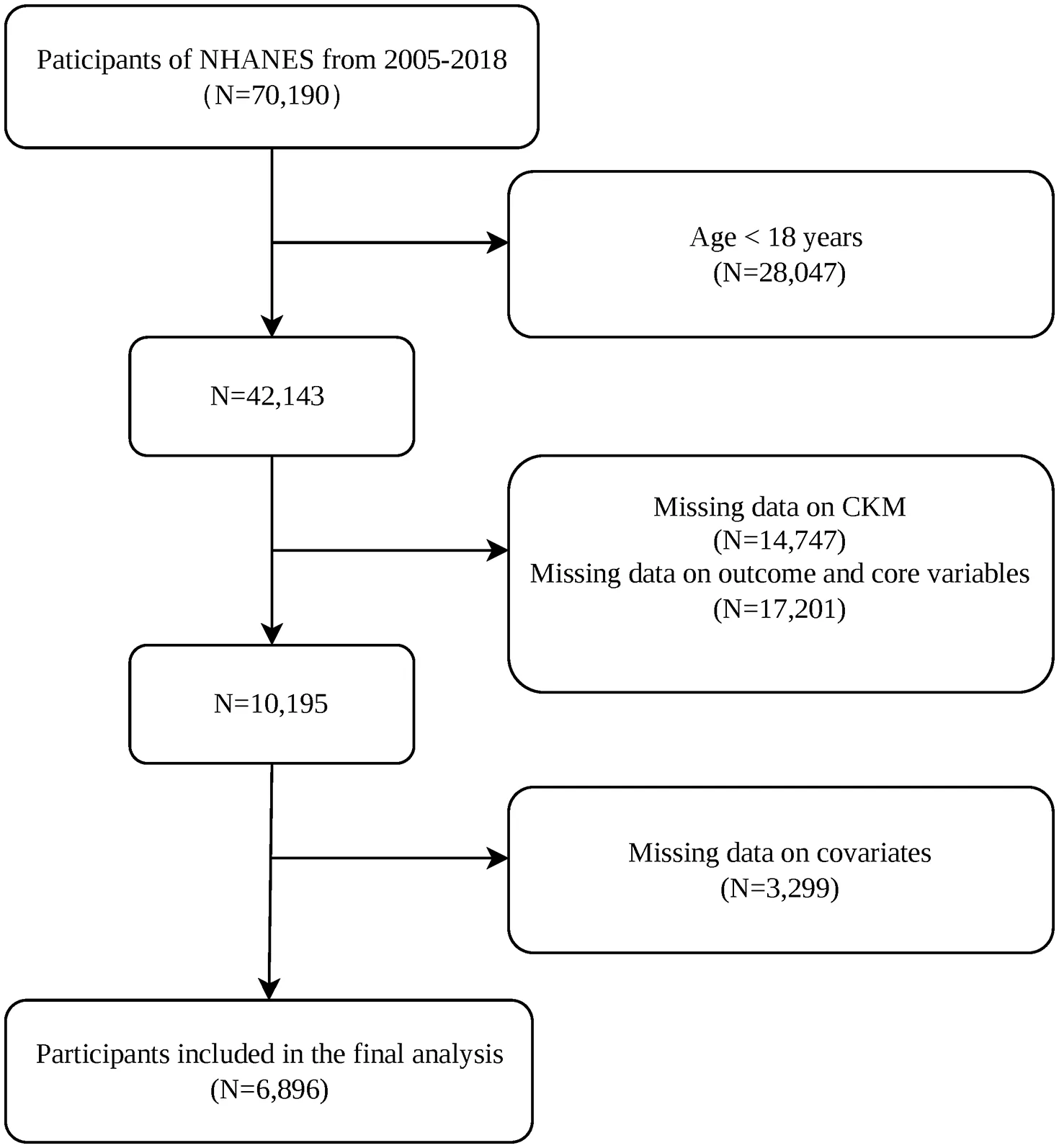
Flowchart of participant selection in NHANES (2005–2018). **Legend:** In the NHANES database, each participant was assigned a unique SEQN identifier. Across different data collection cycles, the same participant’s records were linked and consolidated using their respective SEQN identifiers.

### Hospital validation cohort

To provide a real-world external validation of the CKMAI, we assembled an independent retrospective cohort from the First Affiliated Hospital of Anhui University of Chinese Medicine. Consecutive patients aged ≥18 years with available data for CKM staging (including eGFR, UACR or urine protein, and clinical CVD history) and all CKMAI component variables were enrolled from January 2024 to December 2025. Patients with missing data for any CKMAI component variable were excluded. The final hospital cohort comprised 261 participants.

In this cohort, detailed household income (PIR) was not available. Therefore, we constructed a locally adapted socioeconomic status (SES) indicator using the following algorithm: participants with employee insurance and urban residence were classified as High SES; those with resident insurance or rural residence with nonfarming occupations were classified as Medium SES; and those with New Rural Cooperative Medical Scheme, self pay, or remote rural residence were classified as Low SES. This three-tier classification corresponds to the PIR tertiles (≥3.5, 1.3–3.5, <1.3) used in the original NHANES-derived model. All participants were of Han Chinese ethnicity and were uniformly coded as “Other” (NHANES category 4) for the ethnicity variable. The same SES approximation and ethnicity coding were applied consistently across all participants.

### Exposure assessment: Definition of CKMAI

CKMAI was defined as a composite score derived from a Cox proportional hazards model optimized for predicting both all‑cause mortality and high‑risk CKM status, as defined in the Methods. Variables were selected from five prespecified domains based on their pathophysiological relevance to CKM syndrome: metabolic stress (body mass index [BMI], waist circumference [WC], waist‑to‑height ratio [WHtR], triglycerides [TG], high‑density lipoprotein cholesterol [HDL‑C], total cholesterol [TC], triglyceride‑to‑HDL ratio [TG/HDL], hemoglobin A1c [HbA1c], fasting glucose [FBG], hypertension, diabetes); kidney injury (urine albumin‑to‑creatinine ratio [UACR], estimated glomerular filtration rate [eGFR], serum creatinine [SCR], blood urea nitrogen [BUN], uric acid [UA], CKD); systemic aging (chronological age); inflammatory‑nutritional status (white blood cell count [WBC], lymphocyte percentage [LYM], mean corpuscular volume [MCV], red cell distribution width [RDW], platelet count [PLT], albumin [ALB], alkaline phosphatase [ALP], vitamin E [VE], vitamin B6 [B6], vitamin C [VC], total energy intake); and psychosocial factors (depression, smoking, alcohol consumption, physical activity, education, poverty‑to‑income ratio [PIR], marital status, ethnicity, and sex). Because participants were enrolled across different NHANES cycles (2005–2018), follow-up durations varied substantially, ranging from 1 to 14 years. To ensure comparability and minimize the impact of differential follow-up, we used time-dependent AUC at fixed time points (3, 5, and 10 years) as the primary discrimination metric for mortality outcomes. This approach standardizes performance evaluation at specific horizons rather than relying on variable individual follow-up lengths. Additionally, CKM stages were assessed at baseline using clinical and laboratory data collected during the index NHANES examination visit, and all outcomes (mortality and CKM high risk status) were measured from baseline forward. This is consistent with the American Heart Association CKM staging framework and the study objectives of baseline risk stratification. All continuous variables were standardized to a mean of 0 and standard deviation of 1; categorical variables were converted to dummy indicators.

To construct CKMAI, we employed a two-stage machine learning framework that systematically evaluated over 100 candidate models. The full set of standardized variables was entered into each candidate model without prescreening. Model performance was assessed in the independent testing set using two metrics: Harrell’s C-index for all-cause mortality and AUC for high-risk CKM status (CKM stage ≥3).

The optimal model was selected via Pareto front optimization, which identifies models that are not dominated by any other in terms of both performance metrics. Specifically, a model was considered Pareto optimal if no other model achieved a higher C index without a lower AUC, or vice versa. The model located on the Pareto frontier with the highest sum of standardized ranks for the two metrics (C index and AUC) was selected as the final model. This approach defines “highest combined performance” as the maximum sum of standardized ranks, ensuring a balanced optimization of both endpoints without requiring arbitrary weighting. Among all candidate models, a simple Cox proportional hazards model consistently achieved the highest combined performance and was therefore selected as the final model for CKMAI construction. For all validation analyses, the CKMAI was calculated using the fixed original weights and the standardization parameters (means and SDs) derived from the original 70% training set, unless otherwise specified. This ensures that the index is applied consistently across all cohorts without refitting the model. For the temporal validation analysis where retraining was performed, the CKMAI weights and standardization parameters were derived from the early period training set (2005–2010) and applied to the late period test set (2011–2018); this is explicitly noted as “retrained” in the results.

Coefficients from the final model were normalized to a maximum absolute weight of 1. CKMAI was defined as the weighted sum of standardized variables:


$$\begin{array}{c} \text{CKMAI}=\sum_{i=\text{1}}^{p}w_{i}\times X_{i} \end{array}$$

where $w_{i}$ is the normalized weight for the i-th variable and $X_{i}$ is its standardized value. To enhance interpretability, CKMAI was linearly transformed to a 0–100 scale:


$$\begin{array}{c} {\text{CKMAI}}_{\text{0}-\text{1}\text{0}\text{0}}=\text{1}\text{0}\text{0}\times \frac{\text{CKMAI}-\text{min}\left({\text{CKMAI}}_{\text{train}}\right)}{\text{max}\left({\text{CKMAI}}_{\text{train}}\right)-\text{min}\left({\text{CKMAI}}_{\text{train}}\right)} \end{array}$$

### Two‑stage machine learning model selection

All machine learning analyses were conducted in R version 4.4.0 (R Foundation for Statistical Computing). The construction of CKMAI involved a comprehensive evaluation of feature selection methods and modeling approaches.

This stability selection stage was a feature screening procedure only; no predictive performance metrics (C index or AUC) were calculated during this step. Variables with selection frequency ≥60% across bootstrap samples were retained as candidates for the subsequent model training stage: univariate screening (based on log‑rank test), Lasso [22], Ridge, elastic net with α = 0.4, 0.6, and 0.8 [23], stepwise Cox regression (both directions, backward, forward), CoxBoost [24], supervised principal components, partial least squares Cox regression, random survival forest [25], and gradient boosting machine [26]. For penalized regression methods, the optimal regularization parameter was determined via 3 fold cross validation (nfolds = 3) using the one standard error rule. For stepwise procedures, selection was based on the Akaike information criterion. For tree-based and boosting methods, default hyperparameters were used as specified in the R packages (randomForestSRC and gbm), with detailed tuning ranges provided in S2 Methods. The complete hyperparameter specifications for all 14 feature selection strategies and 15 modeling approaches are provided in the Supporting information to ensure full reproducibility.

Model building employed 15 approaches: simple Cox proportional hazards, Lasso, Ridge, elastic net (α = 0.4, 0.6, 0.8), stepwise Cox (both, backward, forward), CoxBoost, supervised principal components, partial least squares Cox, survival support vector machine, random survival forest, and gradient boosting machine. Each feature selection strategy was combined with each modeling approach, generating 210 candidate models (14 feature selection strategies × 15 modeling approaches). After removing combinations that failed to converge or produced invalid results, 182 models were successfully evaluated. Model performance was evaluated in the testing set using Harrell’s C‑index and the AUC for high‑risk CKM status. These two metrics were used as the primary criteria for model selection. Cardiovascular mortality and renal mortality were evaluated during validation but were not used for model selection (renal mortality was excluded from formal analysis due to only 10 events).

All candidate models were trained on the training set and evaluated in the independent testing set using two performance metrics: Harrell’s C‑index for all‑cause mortality and the area under the curve (AUC) for high‑risk CKM status (CKM stage ≥3). The optimal model was selected via Pareto front optimization, which identifies models that are not dominated by any other in terms of both metrics. Specifically, a model was considered optimal if no other model achieved a higher C‑index without a lower AUC, or vice versa. The model located at the Pareto frontier with the highest sum of standardized ranks for the two metrics was selected as the final model.

To enhance interpretability of the selected model, SHapley Additive exPlanations (SHAP) [27] were applied. SHAP values, which quantify the contribution of each variable to individual mortality risk predictions, were calculated using the fastshap package in R. For each participant in the testing set, the SHAP value for each variable was computed as the average marginal contribution of that variable across all possible coalitions of features. Variable importance was subsequently summarized as the mean absolute SHAP value across all participants, and individual SHAP contributions were visualized to illustrate the direction and magnitude of each variable’s effect. SHAP analyses were performed using the final selected model.

### Outcome ascertainment

All‑cause mortality was the primary outcome. Mortality data were obtained through probabilistic linkage to the National Death Index records, a federally maintained mortality database aggregating state death certificate data [28]. Follow‑up duration extended from the NHANES examination date until the date of death or December 31, 2019, whichever occurred first. Participants who were alive at the end of follow‑up were censored. Cardiovascular mortality was defined as death attributed to cardiovascular or cerebrovascular causes, identified by International Classification of Diseases, 10th Revision (ICD‑10) codes I00–I09, I11, I13, I20–I51, or I60–I69. For brevity, cardiovascular mortality is hereafter termed cardiovascular mortality. Operationally, cardiovascular deaths were identified using values 001 and 005 in the “ucode_leading” field of death records, per NCHS 2019 Public‑Use Linked Mortality File codebook. Kidney‑related deaths were considered in the stability screening phase of model development but were not included in the primary analyses due to the small number of events (*n* = 10). Kidney‑related deaths were defined using ICD‑10 codes N00–N19, N25–N29, or I12–I13. Including such a small number of events would risk model overfitting and unstable estimates; therefore, these outcomes were not pursued further.

CKM syndrome stages were defined according to the American Heart Association framework [29,30]. Stage 0 was defined as BMI < 25 kg/m^2^, absence of hypertension, absence of diabetes, eGFR ≥ 60 mL/min/1.73 m^2^, and UACR < 30 mg/g. Stage 1 was defined as BMI ≥ 25 kg/m^2^ without meeting stage 2 criteria. Stage 2 was defined as the presence of hypertension (systolic blood pressure ≥ 140 mmHg or diastolic blood pressure ≥90 mmHg, or self‑reported diagnosis or antihypertensive medication use), diabetes (hemoglobin A1c ≥ 6.5% or fasting glucose ≥ 126 mg/dL, or self‑reported diagnosis or antidiabetic medication use), or dyslipidemia (triglycerides ≥ 150 mg/dL or high‑density lipoprotein cholesterol < 40 mg/dL in men or < 50 mg/dL in women, or use of lipid‑lowering medications). Stage 3 was defined as eGFR < 45 mL/min/1.73 m^2^ or UACR > 300 mg/g. Stage 4 was defined as a history of cardiovascular disease (self‑reported physician diagnosis of congestive heart failure, coronary heart disease, angina pectoris, myocardial infarction, or stroke). High‑risk CKM status, a secondary outcome, was defined as CKM stage ≥ 3 (i.e., stage 3 or stage 4). This combined definition was chosen to align with the clinical objective of identifying patients who warrant intensified intervention, as both stages represent advanced disease requiring escalation of care, and delaying action until stage 4 would miss the window for early prevention. This combined definition was chosen to align with the clinical objective of identifying patients who warrant intensified intervention, as both stages represent advanced disease requiring escalation of care, and delaying action until stage 4 would miss the window for early prevention.

### Covariates

This study utilized data collected according to standardized protocols within NHANES. Covariates were selected based on prior literature and clinical relevance, encompassing demographic characteristics, lifestyle factors, comorbid conditions, and laboratory measurements. Demographic variables included age (continuous, years), sex (male or female), race and ethnicity (categorized as Mexican American, non‑Hispanic White, non‑Hispanic Black, or other), educational attainment (less than high school, high school or equivalent, or college or above), marital status (married or living with partner, single [widowed/divorced/separated], or never married), and the poverty‑to‑income ratio (PIR; categorized as <1.3, 1.3 to <3.5, or ≥3.5). Lifestyle factors comprised smoking status (never [<100 cigarettes in lifetime] or ever [≥100 cigarettes in lifetime]), alcohol consumption (never [<12 drinks per year] or ever [≥12 drinks per year]), and physical activity (low intensity: light sweating or small increases in breathing/heart rate for at least 10 min over the past 30 days; high intensity: activity causing heavy sweating or large increases in breathing/heart rate). Comorbidities were defined using standardized criteria: hypertension was defined as systolic blood pressure ≥ 140 mmHg or diastolic blood pressure ≥ 90 mmHg, or self‑reported diagnosis or antihypertensive medication use [29]; diabetes was defined as hemoglobin A1c ≥ 6.5% or fasting glucose ≥ 126 mg/dL [30], or self‑reported diagnosis or antidiabetic medication use [31]; dyslipidemia was defined as triglycerides ≥ 150 mg/dL or high‑density lipoprotein cholesterol < 40 mg/dL in men or < 50 mg/dL in women, or use of lipid‑lowering medications [32]; CKD was defined as eGFR < 60 mL/min/1.73 m^2^ or UACR ≥ 30 mg/g [33]; CVD was defined as self‑reported physician diagnosis of congestive heart failure, coronary heart disease, angina pectoris, myocardial infarction, or stroke; cancer was defined as self‑reported physician diagnosis of any malignancy; and depression was defined as a Patient Health Questionnaire‑9 (PHQ‑9) score ≥ 10 or self‑reported physician diagnosis [34]. Laboratory parameters included eGFR (calculated using the CKD‑EPI creatinine equation), UACR, hemoglobin A1c (%), fasting glucose (mg/dL), total cholesterol (mg/dL), triglycerides (mg/dL), high‑density lipoprotein cholesterol (mg/dL), white blood cell count (×1,000 cells/μL), lymphocyte percentage (%), mean corpuscular volume (fL), red cell distribution width (%), platelet count (×1,000 cells/μL), albumin (g/dL), alkaline phosphatase (U/L), blood urea nitrogen (mg/dL), serum creatinine (mg/dL), uric acid (mg/dL), vitamin E (mg), vitamin B6 (mg), vitamin C (mg), and total energy intake (kilocalories). BMI was calculated as weight in kilograms divided by height in meters squared (kg/m^2^). All continuous variables were assessed for normality using the Shapiro–Wilk test; variables with a normal distribution are reported as mean (SD) and those with nonnormal distribution as median (IQR) in descriptive analyses. For modeling, continuous variables were standardized to a mean of 0 and standard deviation of 1, and categorical variables were converted to dummy indicators. No missing data were present for any covariate after the sequential exclusions described in the Study Population section.

### Statistical analysis

All statistical analyses accounted for the complex multistage probability sampling design of NHANES using survey weights, stratification, and clustering variables as recommended by the National Center for Health Statistics. Continuous variables are presented as mean (SD) or median (IQR) based on normality assessed by the Shapiro–Wilk test, and categorical variables as number (%). Baseline characteristics were reported for the overall cohort without group comparisons, as inferential testing is not recommended for descriptive purposes in observational studies. All analyses were prespecified unless explicitly stated as exploratory (e.g., post‑hoc subgroup analyses are clearly indicated). For the primary analyses, weighted Cox proportional hazards regression models were used to estimate HRs and 95% confidence intervals (CIs) for all‑cause and cardiovascular mortality, and weighted logistic regression models were used to estimate odds ratios (ORs) and 95% CIs for high‑risk CKM status. Three hierarchical adjustment models were constructed: Model 1 was unadjusted; Model 2 adjusted for age and sex; and Model 3 was fully adjusted for sex, ethnicity, education, marital status, poverty‑to‑income ratio, smoking, drinking, physical activity, hypertension, diabetes, cancer, and age. CKMAI acceleration (CKMAI_advance). To evaluate whether the CKMAI captures aging-related information beyond chronological age, we calculated CKMAI_advance = CKMAI − Age. A positive value indicates that an individual‘s CKM specific aging exceeds what would be expected based on chronological age alone (aging acceleration), whereas a negative value indicates relative deceleration (aging deceleration). The independent associations of CKMAI_advance with all cause mortality, cardiovascular mortality, and high risk CKM status were assessed using weighted Cox proportional hazards regression (for mortality outcomes) and weighted logistic regression (for CKM high risk status). Two hierarchical adjustment models were constructed: Model 1 adjusted for age and sex; Model 2 was fully adjusted for age, sex, ethnicity, education, marital status, poverty to income ratio, smoking, drinking, physical activity, hypertension, diabetes, and cancer. These models were designed to isolate the independent effect of aging acceleration beyond chronological age and traditional risk factors. The proportional hazards assumption was evaluated using Schoenfeld residuals, and no violations were detected. Discrimination was assessed using time‑dependent AUC and Harrell’s C‑index; reclassification was evaluated using NRI and IDI; calibration was assessed using calibration plots and the Greenwood‑Nam‑D’Agostino test; and clinical utility was assessed using DCA. Multiplicative interactions between CKMAI and CKM stage were tested using likelihood ratio tests, and additive interactions between CKMAI and PhenoAge, KDM, and CMI were quantified using RERI and AP with 95% CIs estimated via bootstrap. To assess whether the super additive interactions could be influenced by collinearity, we calculated variance inflation factors (VIF) for CKMAI, PhenoAge, KDM, and CMI in the fully adjusted models. VIF values below 5 indicate no substantial collinearity. Potential non‑linear relationships between CKMAI and outcomes were examined using restricted cubic splines with knots placed at the 5th, 35th, 65th, and 95th percentiles; the linearity assumption was tested using likelihood ratio tests comparing the spline model with a linear model. Threshold effects were further evaluated using a two‑piecewise linear regression approach: a standard linear model was first fitted, followed by a piecewise model with an inflection point determined by the segmented package, and the likelihood ratio test was used to compare the two models to determine whether a threshold effect was present. To explore the interrelationships among key variables, a correlation matrix was constructed using Pearson correlation coefficients with ggpairs, visualizing distributions on the diagonal, scatter plots with linear regression fits in the lower triangle, and correlation coefficients with significance levels in the upper triangle. Unsupervised clustering was performed using Gaussian mixture models based on the variables with the highest normalized weights in CKMAI (age, RDW, MCV, ALB, ALP, and PhenoAge); the optimal number of clusters was determined by the Bayesian information criterion and silhouette coefficient, and mortality risks across clusters were estimated using weighted Cox regression with cluster 1 (the healthiest phenotype) as the reference. To compare the predictive performance of CKMAI across CKM stages, stratified analyses were conducted for all‑cause mortality, cardiovascular mortality, and high‑risk CKM status, with interaction terms included to test for effect modification. Kaplan‑Meier survival curves were generated to visualize mortality differences across CKMAI tertiles, with log‑rank tests used for group comparisons; risk tables were provided at specified time points. A nomogram was developed based on the final Cox model to enable individualized prediction of 5‑year and 10‑year all‑cause mortality, with calibration plots assessing prediction accuracy. Mediation analyses were conducted using a weighted bootstrap approach with 1,000 replicates to estimate the average causal mediation effect (ACME), average direct effect (ADE), and proportion mediated, with depression score as the mediator and all‑cause mortality, cardiovascular mortality, and high‑risk CKM status as outcomes, adjusting for the same covariates as in Model 3. To test the robustness of the mediation direction, we performed two complementary sensitivity analyses. First, a reverse mediation analysis was conducted with depression score as the exposure, CKMAI as the mediator, and each outcome as the dependent variable, using a PSU level cluster bootstrap (500 resamples) to account for the NHANES complex survey design. Second, a moderation analysis was performed by including the interaction term CKMAI × Depression in the fully adjusted models for all three outcomes. Prespecified subgroup analyses were performed by age (<65 versus ≥65 years), sex, race and ethnicity, education, PIR, smoking, drinking, physical activity, hypertension, diabetes, CKD, cancer, and cardiovascular disease; interaction terms were introduced into the fully adjusted models, and *P* values for interaction were adjusted for multiple comparisons using the Benjamini–Hochberg false discovery rate (FDR) method (q < 0.1). The FDR threshold of 0.1 was chosen to balance the risk of type I and type II errors in these exploratory interaction analyses, as interaction tests are inherently low powered. Importantly, the primary interpretation of subgroup findings was based on the consistency of effect estimates (HRs and ORs) across strata and their 95% CIs, rather than solely on interaction *P* values. To further assess generalizability, we evaluated the discriminative performance (C index, time dependent AUC, and AUC for CKM high risk status) and calibration of the CKMAI stratified by sex and ethnicity. Subgroup analyses that were not prespecified are explicitly indicated as exploratory. Sensitivity analyses included unweighted analyses, restriction of follow‑up to 5 years, and exclusion of participants with baseline cardiovascular disease or CKD to assess the robustness of the findings. To assess the potential impact of selection bias, we compared baseline characteristics between included participants (*n* = 6,896) and excluded participants who were aged ≥18 years (*n* = 35,247), using standardized mean differences (SMD) to quantify between group differences. As an additional sensitivity analysis, we performed multiple imputation by chained equations (MICE) on the full adult NHANES sample (*n* = 42,143 after excluding age <18 years), generating 20 imputed datasets. In each imputed dataset, CKMAI scores were calculated using the fixed original weights and standardization parameters, and predictive performance for all cause mortality, cardiovascular mortality, and CKM high risk status was evaluated for CKMAI, PhenoAge, KDM, and CMI. As additional sensitivity analyses, we compared the predictive performance of the CKMAI (single index) with models using the original variables directly. For all cause mortality, a Cox model incorporating all 18 original CKMAI components was fitted; for CKM high risk status, a logistic model using the 8 variables that passed stability selection for this outcome was fitted. Performance was evaluated using time-dependent AUC (for mortality) and standard AUC (for CKM high risk status) in the independent test set. To account for the competing risk of noncardiovascular death, we supplemented the standard Cox regression for cardiovascular mortality with a Fine Gray subdistribution hazard model, treating noncardiovascular deaths as the competing event. The results were compared with the cause specific HR from the standard Cox model. Because kidney‑related deaths were too few (*n* = 10) to support stable model estimation, formal analyses for this outcome were not performed. Model interpretability using SHAP values is described in the Two‑Stage Machine Learning Model Selection section. All statistical tests were two‑sided, and a *P* value < 0.05 was considered statistically significant. A web based risk calculator implementing the final CKMAI model is available at https://zhuzhengyang.shinyapps.io/shiny/ to facilitate clinical application. An open source R package (CKMAIcalc) implementing the same framework is available at https://github.com/zhuzhengyang517/CKMAIcalc; detailed usage instructions are provided in S3 Methods. This study was conducted according to the analysis plan provided in S1 Protocol. No deviations from the prespecified analysis plan occurred. All analyses were performed as described, and any post‑hoc exploratory analyses are explicitly indicated as such in the manuscript. All statistical analyses were performed using R version 4.4.0 (R Foundation for Statistical Computing) with the survey, survival, rms, mediation, segmented, and other relevant packages. No artificial intelligence (AI) tools or technologies were used in any aspect of this study, including data collection, data analysis, interpretation of results, generation of content, preparation of figures, or writing of the manuscript. The authors assume full responsibility for the accuracy, integrity, and originality of all content presented in this manuscript.

### Temporal and external validation

To assess the generalizability of the CKMAI beyond the random 70/30 split, we performed three complementary validation analyses.

First, we conducted temporal validation by retraining the entire two stage machine learning pipeline (stability selection, multi-model training, and Pareto front optimization) using the early NHANES period (2005–2010) as the training set and evaluating performance on the late period (2011–2018). The same feature selection methods, modeling approaches, and performance metrics used in the primary analysis were applied.

Second, we performed fixed weight temporal validation to assess whether the original CKMAI formula itself generalizes across different survey cycles without retraining. The original CKMAI weights (derived from the 70% random split of the full 2005–2018 training set) were applied to the early and late periods separately, using the original standardization parameters (means and SDs from the 70% training set). To test the robustness of the standardization approach, we also re standardized the early data using the means and SDs from the late period, and re standardized the late data using the means and SDs from the early period, while keeping the CKMAI weights fixed. Performance was evaluated using Harrell’s C index for all cause and cardiovascular mortality, and AUC for high risk CKM status. Bootstrap CIs (500 resamples) were calculated for all metrics.

Third, we performed external validation in an independent hospital cohort (*n* = 261) from the First Affiliated Hospital of Anhui University of Chinese Medicine. Because detailed household income (PIR) was not available in the medical records, we constructed a locally adapted three-tier socioeconomic status (SES) indicator based on insurance type (employee, resident, or New Rural Cooperative Medical Scheme/self pay), residence (urban versus rural), and occupation (professional/worker/farmer/unemployed), corresponding to the PIR tertiles (≥3.5, 1.3–3.5, <1.3) used in the primary model. Ethnicity was uniformly coded as “Other” (NHANES category 4), reflecting the Han Chinese composition of the cohort. As the CKMAI model is defined with a fixed set of component variables derived from the primary analysis, removing any component would alter the index structure and compromise the comparability of the external validation. To enable the complete implementation of the CKMAI model in this cohort, educational categories were derived based on occupation, workplace, and residential address, which were the only available proxies for socioeconomic status. Importantly, this variable carries a relatively low weight in the CKMAI model (normalized weight of 0.143), reducing the potential impact of estimation‑related uncertainty on the overall CKMAI calculation. Three categories (less than high school, high school or equivalent, and college or above) were defined to approximate the NHANES educational classifications used in the primary analysis.

CKMAI and the comparator indices (PhenoAge, KDM, and CMI) were calculated using the fixed original weights and standardization parameters. The primary outcome for this validation was high risk CKM status (CKM stage ≥3), defined according to AHA criteria using available clinical and laboratory data. Discrimination was assessed using AUC with DeLong 95% CIs, and pairwise comparisons against CKMAI were performed using DeLong’s test. Detailed definitions of the SES approximation and educational category derivation are provided in the S1 Methods.

## Results

### Study population and baseline characteristics

After applying the exclusion criteria, the final analytical cohort comprised 6,896 participants, when weighted to account for the complex survey design, the analytical cohort was representative of the noninstitutionalized US adult population (2005–2018). The median age of the cohort was 48.0 years (IQR 34.0–60.0), and 3,293 participants (48.3%) were female. The distribution across CKM stages was as follows: stage 0 (*n* = 506, 7.3%), stage 1 (*n* = 1,118, 16.2%), stage 2 (*n* = 4,105, 59.5%), stage 3 (*n* = 374, 5.4%), and stage 4 (*n* = 793, 11.5%). Over a median follow‑up of 9.2 years (IQR 5.6–14.1), 660 all‑cause deaths (6.6%) and 201 cardiovascular deaths (2.1%) were documented. The baseline characteristics of the overall cohort stratified by CKM stage are presented in Table 1. Participants in higher CKM stages were older, had higher BMI, waist circumference, and a more adverse cardiometabolic profile, including higher prevalence of hypertension, diabetes, and CKD. Detailed baseline characteristics of the training set (*n* = 4,827) and testing set (*n* = 2,069) are provided in S1 and S2 Tables, respectively; the two sets were well balanced with no significant differences in key demographic and clinical variables.

**Table 1:** Baseline characteristics of the study population overall and by CKM stage.

Variable	Total (*n* = 6,896)	Stage 0 (*n* = 506)	Stage 1 (*n* = 1,118)	Stage 2 (*n* = 4,105)	Stage 2 (*n* = 4,105)	Stage 4 (*n* = 793)
Age, median (IQR), years	48.00 (34.00, 60.00)	30.00 (24.00, 42.00)	40.00 (29.00, 51.00)	50.00 (36.00, 61.00)	51.00 (45.00, 62.00)	65.00 (56.00, 74.00)
BMI, median (IQR), kg/m²	28.15 (24.26, 32.91)	21.30 (19.70, 22.68)	26.96 (24.12, 30.80)	29.27 (25.63, 34.00)	30.87 (28.00, 34.00)	29.60 (25.39, 34.38)
WC, median (IQR), cm	98.80 (87.80, 110.40)	76.90 (72.90, 82.30)	94.40 (87.00, 104.20)	101.40 (92.00, 112.70)	108.00 (100.40, 115.20)	105.20 (94.70, 115.80)
WHtR, median (IQR)	0.58 (0.52, 0.65)	0.46 (0.44, 0.49)	0.56 (0.51, 0.61)	0.60 (0.54, 0.67)	0.62 (0.58, 0.66)	0.62 (0.56, 0.69)
Depression score, median (IQR)	2.00 (0.00, 4.00)	2.00 (0.00, 3.00)	1.00 (0.00, 4.00)	2.00 (0.00, 4.00)	1.00 (0.00, 3.00)	2.00 (0.00, 5.00)
WBC, median (IQR), ×1,000 cells/μL	6.50 (5.50, 7.80)	5.80 (4.90, 7.00)	6.30 (5.40, 7.50)	6.60 (5.60, 8.00)	6.90 (5.70, 8.40)	6.80 (5.60, 8.10)
LYM, median (IQR), %	30.00 (24.80, 35.30)	32.40 (26.90, 37.10)	30.90 (26.30, 35.50)	30.00 (24.70, 35.30)	27.90 (23.00, 32.80)	26.90 (21.30, 32.80)
MCV, median (IQR), fL	89.70 (86.60, 92.60)	90.60 (87.60, 92.90)	89.40 (86.60, 92.10)	89.50 (86.50, 92.40)	89.80 (86.10, 92.70)	90.50 (87.20, 93.50)
RDW, median (IQR), %	13.00 (12.40, 13.60)	12.80 (12.30, 13.20)	12.90 (12.50, 13.50)	13.00 (12.40, 13.60)	13.00 (12.40, 13.50)	13.40 (12.80, 14.00)
HbA1c, median (IQR), %	5.50 (5.20, 5.80)	5.20 (5.00, 5.30)	5.40 (5.10, 5.60)	5.50 (5.20, 5.80)	5.70 (5.40, 6.20)	5.80 (5.40, 6.30)
ALB, median (IQR), g/dL	4.20 (4.00, 4.50)	4.40 (4.10, 4.60)	4.30 (4.10, 4.50)	4.20 (4.00, 4.40)	4.30 (4.10, 4.50)	4.10 (3.90, 4.30)
ALP, median (IQR), U/L	66.00 (54.00, 80.00)	56.00 (47.00, 67.00)	63.00 (53.00, 77.00)	68.00 (56.00, 81.00)	66.00 (56.00, 81.00)	72.00 (59.00, 89.00)
BUN, median (IQR), mg/dL	13.00 (11.00, 16.00)	12.00 (10.00, 14.00)	13.00 (11.00, 16.00)	13.00 (11.00, 16.00)	15.00 (12.00, 18.00)	15.00 (12.00, 20.00)
UACR, median (IQR), mg/g	6.29 (4.24, 11.31)	6.10 (4.25, 9.26)	5.22 (3.91, 7.61)	6.47 (4.35, 12.16)	7.16 (4.45, 19.15)	9.30 (5.34, 22.72)
EGFR, median (IQR), mL/min/1.73m^2^	98.56 (83.83, 111.55)	110.60 (97.19, 122.34)	103.13 (90.56, 116.10)	98.04 (83.23, 110.17)	92.37 (77.88, 105.12)	82.94 (66.53, 95.26)
TG, median (IQR), mg/dL	105.00 (71.00, 156.00)	62.00 (47.00, 79.00)	77.00 (56.00, 98.00)	133.00 (87.00, 180.00)	136.00 (96.00, 202.00)	115.00 (79.00, 169.00)
HDL, median (IQR), mg/dL	51.00 (42.00, 63.00)	64.00 (53.00, 74.00)	55.00 (47.00, 66.00)	49.00 (41.00, 60.00)	44.00 (37.00, 52.00)	48.00 (40.00, 60.00)
TG/HDL, median (IQR)	2.00 (1.18, 3.53)	1.01 (0.70, 1.35)	1.36 (0.92, 1.90)	2.62 (1.55, 4.18)	3.15 (1.88, 5.11)	2.42 (1.40, 3.95)
CMI, median (IQR)	1.19 (0.65, 2.20)	0.46 (0.32, 0.63)	0.77 (0.49, 1.10)	1.60 (0.91, 2.59)	2.03 (1.14, 3.22)	1.58 (0.85, 2.62)
PHENOAGE, median (IQR)	57.95 (44.18, 71.19)	38.62 (28.84, 48.70)	49.22 (38.39, 60.65)	59.80 (47.63, 72.00)	64.75 (55.32, 77.98)	76.15 (66.52, 84.83)
KDM, median (IQR)	41.00 (29.60, 53.56)	23.88 (18.56, 32.11)	32.74 (25.02, 41.82)	44.43 (33.55, 56.24)	48.60 (37.60, 62.64)	54.40 (42.00, 69.73)
CKMAI, median (IQR)	40.03 (28.02, 51.66)	25.93 (18.50, 34.60)	33.58 (23.66, 44.77)	41.92 (29.78, 51.68)	47.26 (37.98, 58.65)	56.93 (46.78, 63.91)
Weight, median (IQR), kg	81.50 (68.10, 96.70)	59.50 (53.40, 67.00)	79.00 (67.90, 91.20)	84.50 (71.90, 99.30)	95.50 (84.60, 104.70)	84.00 (70.20, 100.00)
Height, median (IQR), cm	169.30 (162.10, 176.70)	167.60 (162.00, 174.10)	169.20 (163.00, 176.90)	169.10 (161.50, 176.70)	175.30 (169.00, 179.60)	169.00 (161.70, 175.80)
VE, median (IQR), mg	0.06 (0.00, 0.35)	0.07 (0.00, 0.54)	0.05 (0.00, 0.34)	0.06 (0.00, 0.35)	0.09 (0.00, 0.38)	0.05 (0.00, 0.25)
B6, median (IQR), mg	0.01 (0.00, 0.15)	0.03 (0.00, 0.17)	0.01 (0.00, 0.15)	0.01 (0.00, 0.16)	0.02 (0.00, 0.14)	0.01 (0.00, 0.11)
VC, median (IQR), mg	0.00 (0.00, 0.20)	0.00 (0.00, 0.10)	0.00 (0.00, 0.10)	0.00 (0.00, 0.20)	0.00 (0.00, 0.40)	0.00 (0.00, 0.00)
TC, median (IQR), mg/dL	190.00 (165.00, 219.00)	173.00 (153.00, 195.00)	185.00 (161.00, 210.00)	197.00 (172.00, 227.00)	186.00 (161.00, 219.00)	173.00 (146.00, 201.00)
PLT, median (IQR), ×1,000 cells/μL	241.00 (205.00, 283.00)	235.00 (202.00, 271.00)	237.00 (205.00, 276.00)	248.00 (210.00, 291.00)	236.00 (197.00, 277.00)	219.00 (186.00, 263.00)
FBG, median (IQR), mg/dL	102.00 (95.00, 110.00)	92.00 (87.00, 95.00)	102.00 (97.00, 107.00)	102.00 (96.00, 112.00)	109.00 (101.00, 126.00)	108.00 (99.00, 125.00)
SCR, median (IQR), mg/dL	0.86 (0.73, 1.00)	0.80 (0.70, 0.90)	0.84 (0.72, 0.96)	0.85 (0.72, 1.00)	0.96 (0.84, 1.11)	0.93 (0.81, 1.09)
UA, median (IQR), mg/dL	5.50 (4.60, 6.40)	4.50 (3.90, 5.30)	5.20 (4.40, 6.00)	5.70 (4.80, 6.60)	6.20 (5.40, 7.10)	5.80 (4.80, 6.80)
Energy Intake, median (IQR), kcal	2013.50 (1536.50, 2589.00)	2031.00 (1545.50, 2571.50)	2081.50 (1614.00, 2627.00)	2000.00 (1527.00, 2589.00)	2165.00 (1637.00, 2781.50)	1777.50 (1397.00, 2298.00)
Follow up, median (IQR), months	62.00 (36.00, 137.00)	58.00 (36.00, 141.00)	51.00 (33.00, 131.00)	110.00 (36.00, 139.00)	121.00 (51.00, 144.00)	56.00 (30.00, 124.00)
Sex, %						
Male	33,603 (51.67)	169 (33.66)	580 (52.16)	2065 (50.38)	306 (84.85)	483 (58.94)
Female	33,293 (48.33)	337 (66.34)	538 (47.84)	2040 (49.62)	68 (15.15)	310 (41.06)
Ethnicity, %						
Mexican American	11,125 (7.76)	47 (4.32)	224 (10.20)	731 (8.30)	50 (5.16)	73 (3.88)
Non-Hispanic White	33,219 (70.66)	236 (70.47)	487 (69.09)	1855 (70.20)	195 (74.53)	446 (75.12)
Non-Hispanic Black	11,255 (9.54)	125 (13.38)	162 (7.46)	734 (9.36)	63 (8.72)	171 (11.72)
Other	11,297 (12.04)	98 (11.83)	245 (13.24)	785 (12.14)	66 (11.59)	103 (9.27)
Education, %						
Less than high school	11,565 (14.15)	62 (8.64)	218 (12.04)	951 (14.12)	100 (17.94)	234 (22.74)
High school or equivalent	11,668 (24.60)	109 (19.64)	242 (21.58)	11,002 (25.52)	100 (27.94)	215 (28.48)
College or above	33,663 (61.25)	335 (71.72)	658 (66.38)	22,152 (60.36)	174 (54.12)	344 (48.77)
Marital Status, %						
Married or living with a partner	44,268 (65.29)	250 (56.45)	709 (66.67)	22,569 (65.15)	283 (78.66)	457 (64.76)
Single (widowed/divorced/separated)	11,551 (18.55)	53 (8.16)	180 (14.74)	963 (19.92)	65 (14.79)	290 (31.10)
Never married	11,077 (16.16)	203 (35.39)	229 (18.59)	573 (14.93)	26 (6.55)	46 (4.14)
PIR, %						
<1.3	1941 (18.40)	138 (17.94)	296 (17.38)	11,149 (18.21)	98 (16.14)	260 (23.82)
1.3 to <3.5	22,723 (36.53)	184 (33.08)	424 (35.38)	11,639 (36.88)	132 (33.12)	344 (42.39)
≥3.5	22,232 (45.07)	184 (48.98)	398 (47.24)	11,317 (44.92)	144 (50.75)	189 (33.80)
Smoking, %						
No	33,624 (52.74)	313 (60.23)	646 (56.07)	22,181 (53.34)	172 (44.79)	312 (38.06)
Yes	33,272 (47.26)	193 (39.77)	472 (43.93)	1924 (46.66)	202 (55.21)	481 (61.94)
Drinking, %						
No	22,235 (27.90)	141 (24.08)	315 (23.62)	11,398 (29.62)	84 (20.00)	297 (34.60)
Yes	44,661 (72.10)	365 (75.92)	803 (76.38)	22,707 (70.38)	290 (80.00)	496 (65.40)
Physical Activity, %						
Low physical activity	22,586 (31.98)	130 (21.92)	313 (25.40)	11,607 (34.23)	137 (28.69)	399 (44.26)
High physical activity	44,310 (68.02)	376 (78.08)	805 (74.60)	22,498 (65.77)	237 (71.31)	394 (55.74)
Hypertension, %						
No	33,767 (60.09)	506 (100.00)	11,118 (100.00)	1957 (50.74)	6 (1.00)	180 (26.11)
Yes	33,129 (39.91)	0 (0.00)	0 (0.00)	22,148 (49.26)	368 (99.00)	613 (73.89)
Diabetes, %						
No	55,313 (83.12)	503 (99.73)	11,088 (97.81)	33,090 (80.93)	208 (62.23)	424 (59.72)
Yes	11,583 (16.88)	3 (0.27)	30 (2.19)	11,015 (19.07)	166 (37.77)	369 (40.28)
CKD, %						
No	55,763 (87.80)	506 (100.00)	11,118 (100.00)	33,389 (85.36)	258 (77.57)	492 (70.05)
Yes	11,133 (12.20)	0 (0.00)	0 (0.00)	716 (14.64)	116 (22.43)	301 (29.95)
Cancer, %						
No	66,218 (90.33)	487 (94.71)	11,058 (93.93)	33,740 (90.61)	301 (82.92)	632 (80.17)
Yes	678 (9.67)	19 (5.29)	60 (6.07)	365 (9.39)	73 (17.08)	161 (19.83)
Depression, %						
No	66,328 (93.05)	470 (94.64)	11,046 (94.12)	33,785 (93.24)	348 (94.28)	679 (87.00)
Yes	568 (6.95)	36 (5.36)	72 (5.88)	320 (6.76)	26 (5.72)	114 (13.00)
Mortality Status, %						
No	66,236 (93.45)	499 (98.68)	11,082 (97.93)	33,786 (94.38)	316 (89.55)	553 (73.98)
Yes	660 (6.55)	7 (1.32)	36 (2.07)	319 (5.62)	58 (10.45)	240 (26.02)
Death CVD, %						
No	66,695 (97.91)	506 (100.00)	11,108 (99.30)	44,015 (98.28)	362 (98.22)	704 (89.96)
Yes	201 (2.09)	0 (0.00)	10 (0.70)	90 (1.72)	12 (1.78)	89 (10.04)
Death Renal, %						
No	66,886 (99.93)	506 (100.00)	11,118 (100.00)	44,103 (99.98)	372 (99.68)	787 (99.54)
Yes	10 (0.07)	0 (0.00)	0 (0.00)	2 (0.02)	2 (0.32)	6 (0.46)

Abbreviations: BMI, body mass index; WC, waist circumference; WHtR, waist‑to‑height ratio; WBC, white blood cell count; LYM, lymphocyte percentage; MCV, mean corpuscular volume; RDW, red cell distribution width; PLT, platelet count; HbA1c, glycated hemoglobin; FBG, fasting blood glucose; ALP, alkaline phosphatase; BUN, blood urea nitrogen; SCR, serum creatinine; UA, uric acid; UACR, urine albumin‑to‑creatinine ratio; eGFR, estimated glomerular filtration rate; TC, total cholesterol; HDL, high‑density lipoprotein; TG, triglycerides; CMI, cardiometabolic index; VE, vitamin E; B6, vitamin B6; VC, vitamin C; PHENOAGE, phenotypic age; KDM, KDM biological age; CKMAI, CKM‑specific aging index; PIR, poverty‑to‑income ratio; CKD, chronic kidney disease; CVD, cardiovascular disease. Percentages are weighted to account for complex survey design.

### CKMAI composition and model performance

The final CKMAI was derived from a simple Cox proportional hazards model selected via Pareto front optimization across over 100 candidate models, as defined in the Methods. In the testing set, the model achieved Harrell’s C‑index values of 0.876 for all‑cause mortality, 0.914 for cardiovascular mortality, and 0.991 for renal mortality (the latter not further analyzed due to the small number of events, *n* = 10), and an AUC of 0.790 for high‑risk CKM status (Fig 2). The normalized weights of the 18 component variables are shown in Fig 3; the highest weights were observed for age (1.00), RDW (0.378), sex (0.293), PIR (0.211), ethnicity (0.208), marital status (0.198), MCV (0.177), albumin (0.154), ALP (0.144), education (0.143), and eGFR (0.137). CKMAI values were comparable between the training set (mean 40.95, SD 16.59) and the testing set (mean 41.14, SD 16.89; S3 Table).

**Fig 2:**
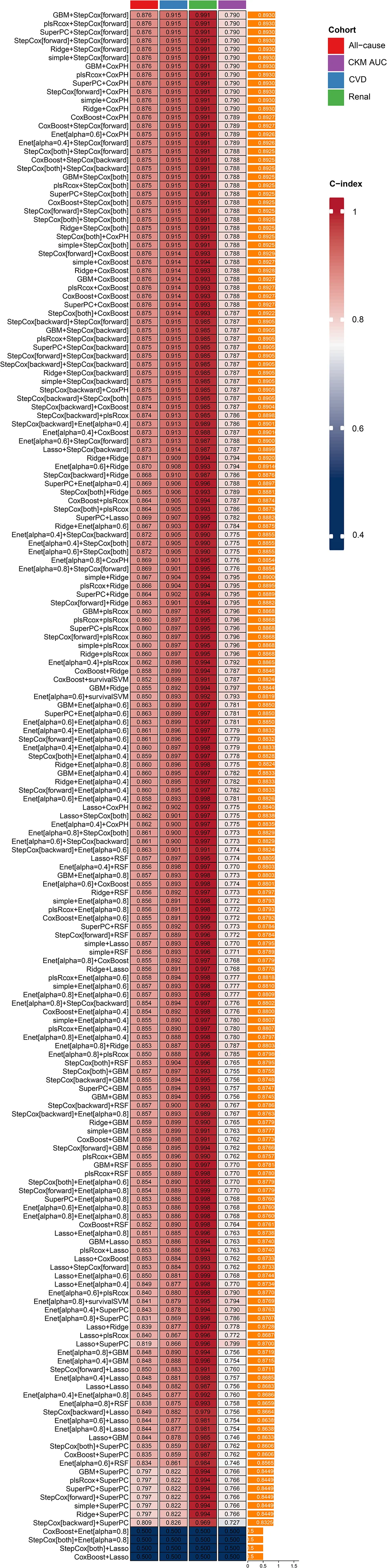
Heatmap of Harrell’s C‑index across candidate models for all‑cause, cardiovascular, renal, and CKM high‑risk outcomes. **Legend:** The heatmap displays C‑index values (color gradient from blue to red) for over 100 candidate models (rows) across four outcomes: all‑cause mortality, cardiovascular mortality, renal mortality, and CKM high‑risk status (area under the curve, AUC). The right bar shows the average C‑index per model. The simple Cox proportional hazards model (highlighted) achieved the highest combined performance and was selected as the final model for CKMAI construction. C‑index, Harrell’s concordance index; AUC, area under the curve; CKM, cardiovascular‑kidney‑metabolic; CVD, cardiovascular disease; CKMAI, CKM‑specific aging index.

**Fig 3:**
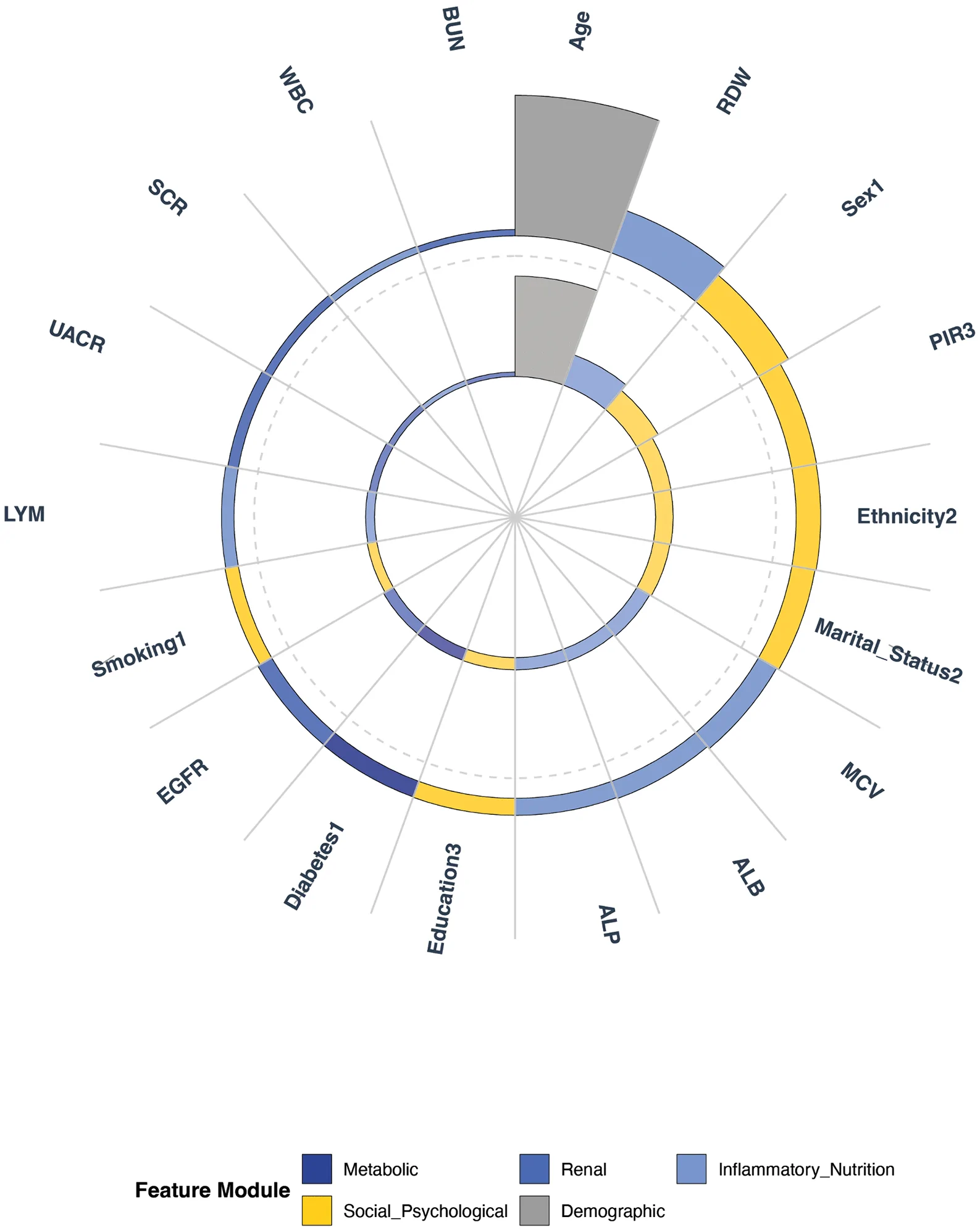
Normalized weights of the 18 component variables comprising CKMAI. **Legend:** Variables are grouped by domain: demographic, metabolic, renal, inflammatory‑nutritional, and social‑psychological. Age had the highest weight (normalized to 1.00), followed by RDW, sex (Sex1 = male), PIR3 (poverty‑income ratio ≥3.5), Ethnicity2 (Non‑Hispanic White), Marital_Status2 (single, widowed/divorced/separated), MCV, albumin, ALP, Education3 (college or above), Diabetes1 (yes), eGFR, Smoking1 (yes), LYM, UACR, SCR, WBC, and BUN. RDW, red cell distribution width; PIR, poverty‑to‑income ratio; MCV, mean corpuscular volume; ALP, alkaline phosphatase; eGFR, estimated glomerular filtration rate; LYM, lymphocyte percentage; UACR, urine albumin‑to‑creatinine ratio; SCR, serum creatinine; WBC, white blood cell count; BUN, blood urea nitrogen; CKMAI, CKM‑specific aging index.

To explore the relationships between CKMAI, established biological age indices, and key outcomes, we performed correlation analyses. As shown in S1 Fig, CKMAI exhibited strong positive correlations with both PhenoAge and KDM, whereas its correlation with chronological age was weak. PhenoAge and KDM were also highly correlated with each other. S2 Fig displays the correlation matrix among the 18 component variables of CKMAI, mortality outcomes, and CKM high‑risk status. Notably, CKM high‑risk status was strongly correlated with cardiovascular mortality and all‑cause mortality. eGFR showed strong negative correlations with age and UACR, consistent with expected renal physiology. Given the strong positive correlations between CKMAI and established biological age indices (PhenoAge and KDM), we further conducted additive interaction analyses to explore whether combining these measures exhibits synergistic effects on mortality and CKM progression.

Bootstrap stability selection was performed to identify features consistently associated with all‑cause mortality and CKM high‑risk status across resampled datasets. The selection frequencies and the overlap of stable features are presented in S3 Fig. Among the 44 candidate variables, 18 were retained as stable predictors for both outcomes, highlighting the robustness of the CKMAI framework. The complete list of stable features and their selection frequencies are provided in S4 Table. The SHAP summary plot, which displays the global feature importance and the direction of individual contributions to mortality predictions, is presented in S4 Fig.

### Predictive performance of CKMAI compared with existing indices

The predictive performance of CKMAI, PhenoAge, KDM, and CMI was evaluated in the independent testing set (Table 2). For all cause mortality, time dependent AUC values at 3, 5, and 10 years were consistently higher for CKMAI (0.893, 95% CI [0.851, 0.925]; 0.907, 95% CI [0.884, 0.927]; and 0.890, 95% CI [0.868, 0.910], respectively) than for PhenoAge (0.865, 95% CI [0.819, 0.894]; 0.870, 95% CI [0.834, 0.891]; 0.850, 95% CI [0.818, 0.878]), KDM (0.768, 95% CI [0.730, 0.826]; 0.805, 95% CI [0.768, 0.847]; 0.781, 95% CI [0.730, 0.816]), and CMI (0.460, 95% CI [0.395, 0.532]; 0.464, 95% CI [0.412, 0.536]; 0.511, 95% CI [0.464, 0.552]) (Fig 4A). DeLong tests confirmed that CKMAI significantly outperformed all other indices at each time point (*P* < 0.01 for all; S5 Table). For cardiovascular mortality, CKMAI’s time dependent AUCs at 3, 5, and 10 years were 0.904 (95% CI [0.840, 0.943]), 0.937 (95% CI [0.892, 0.960]), and 0.937 (95% CI [0.900, 0.959]), respectively, substantially higher than those of PhenoAge (0.879, 95% CI [0.815, 0.922]; 0.902, 95% CI [0.871, 0.929]; 0.908, 95% CI [0.878, 0.941]), KDM (0.773, 95% CI [0.662, 0.847]; 0.801, 95% CI [0.723, 0.869]; 0.834, 95% CI [0.781, 0.871]), and CMI (0.505, 95% CI [0.393, 0.677]; 0.525, 95% CI [0.399, 0.658]; 0.541, 95% CI [0.453, 0.618]) (Fig 4B). For high risk CKM status (CKM stage ≥3), CKMAI also yielded the highest AUC (0.790, 95% CI [0.765, 0.790]), compared with PhenoAge (0.767, 95% CI [0.741, 0.767]), KDM (0.720, 95% CI [0.692, 0.720]), and CMI (0.599, 95% CI [0.568, 0.599]) (Fig 4C).

**Table 2:** Predictive performance of CKMAI, PhenoAge, KDM, and CMI in the testing set.

Outcome	Metric	CKMAI	PhenoAge	KDM	CMI
**All-cause mortality**	Time-dependent AUC (95% CI) – 3 y	0.893 (0.851,0.925)	0.865 (0.819,0.894)	0.768 (0.730,0.826)	0.460 (0.395,0.532)
	Time-dependent AUC (95% CI) – 5 y	0.907 (0.884,0.927)	0.870 (0.834,0.891)	0.805 (0.768,0.847)	0.464 (0.412,0.536)
	Time-dependent AUC (95% CI) – 10 y	0.890 (0.868,0.910)	0.850 (0.818,0.878)	0.781 (0.730,0.816)	0.511 (0.464,0.552)
	NRI (95% CI) – 3 y	0.077 (0.031,0.122)*	0.032 (0.006,0.057)*	0.013 (,0.018,0.045)	0.003 (0.001,0.005)*
	NRI (95% CI) – 5 y	0.064 (0.029,0.100)*	0.022 (,0.002,0.049)	0.004 (–0.022,0.028)	0.003 (0.001,0.005)*
	NRI (95% CI) – 10 y	0.030 (0.002,0.061)*	0.006 (–0.014,0.028)	–0.012 (–0.028,0.003)	0.005 (0.002,0.010)*
	IDI (95% CI) – 3 y	0.007 (0.001,0.012)*	0.000 (–0.002,0.002)	0.002 (–0.000,0.004)	0.000 (–0.000,0.000)
	IDI (95% CI) – 5 y	0.015 (0.007,0.022)*	0.002 (–0.001,0.005)	0.004 (0.000,0.007)*	0.000 (–0.000,0.000)
	IDI (95% CI) – 10 y	0.030 (0.020,0.041)*	0.008 (0.002,0.014)*	0.007 (0.002,0.012)*	0.000 (–0.000,0.001)
**Cardiovascular mortality**	Time-dependent AUC (95% CI) – 3 y	0.904 (0.840,0.943)	0.879 (0.815,0.922)	0.773 (0.662,0.847)	0.505 (0.393,0.677)
	Time-dependent AUC (95% CI) – 5 y	0.937 (0.892,0.960)	0.902 (0.871,0.929)	0.801 (0.723,0.869)	0.525 (0.399,0.658)
	Time-dependent AUC (95% CI) – 10 y	0.937 (0.900,0.959)	0.908 (0.878,0.941)	0.834 (0.781,0.871)	0.541 (0.453,0.618)
**High-risk CKM status**	AUC (95% CI)	0.790 (0.765,0.816)	0.767 (0.741,0.793)	0.720 (0.692,0.749)	0.599 (0.568,0.631)

*P < 0.05 for NRI/IDI (95% CI does not include zero). Abbreviations: AUC, area under the curve; CI, confidence interval; CKM, cardiovascular kidney metabolic; CMI, cardiometabolic index; IDI, integrated discrimination improvement; KDM, KDM biological age; NRI, net reclassification improvement; PHENOAGE, phenotypic age. Time-dependent AUCs were calculated using the timeROC package. NRI and IDI were calculated using the survIDINRI package; 95% CIs were estimated using 200 bootstrap resamples. All NRI and IDI estimates are unadjusted. The variables controlled for in the models from which NRI and IDI were derived include age, sex, ethnicity, education, marital status, poverty to income ratio, smoking, drinking, physical activity, hypertension, diabetes, and cancer (fully adjusted Model 3).

**Fig 4:**
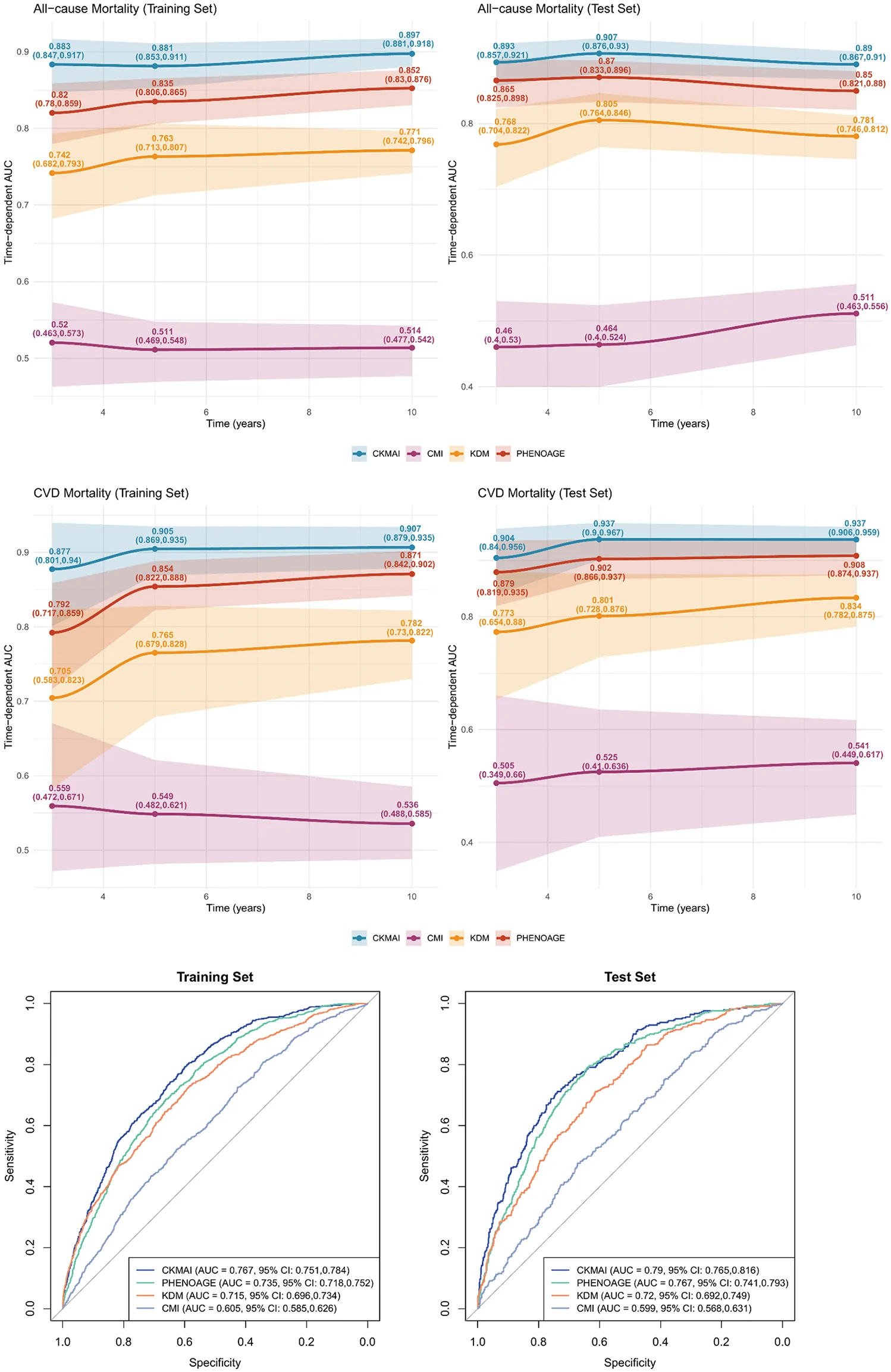
Time‑dependent area under the curve (AUC) curves for all‑cause mortality, cardiovascular mortality, and high‑risk CKM status in the testing set. **Legend:** Panel A corresponds to all‑cause mortality, panel B to cardiovascular mortality, and panel C to high‑risk CKM status. Solid lines represent CKMAI; dashed lines represent PhenoAge, KDM, and CMI. Shaded areas indicate 95% confidence intervals. CKMAI consistently outperformed the other indices at 3, 5, and 10 years. CKMAI, CKM‑specific aging index; KDM, KDM biological age; CMI, cardiometabolic index; CKM, cardiovascular‑kidney‑metabolic; AUC, area under the curve.

NRI and IDI were calculated for all cause mortality (the primary outcome); cardiovascular mortality was not analyzed for these metrics due to the limited number of events (*n* = 201). For all cause mortality, CKMAI showed significant NRI and IDI at 3, 5, and 10 years (e.g., 5 year NRI 0.064, 95% CI [0.029, 0.100]; IDI 0.015, 95% CI [0.007, 0.022]; *P* < 0.05 for all). In contrast, PhenoAge, KDM, and CMI exhibited smaller or nonsignificant improvements, with only occasional significance at specific time points (Table 2). Decision curve analysis (S5 Fig) demonstrated that CKMAI provided superior net benefit across a wide range of threshold probabilities compared with the other indices. Calibration plots (S6 Fig) indicated good agreement between predicted and observed mortality for CKMAI (Greenwood Nam D’Agostino test *P* > 0.05). Corresponding performance metrics for the training set are provided in S6 Table.

To facilitate individualized risk estimation in clinical practice, we developed nomograms based on the final CKMAI model. S7–S9 Figs present the nomograms for predicting 3‑year, 5‑year, and 10‑year all‑cause mortality, cardiovascular mortality, and high‑risk CKM status, respectively. Each nomogram translates the weighted contribution of each CKMAI component into a point scale, allowing clinicians to visually estimate a patient’s risk probability without computational tools. These nomograms also served as the basis for the development of a web‑based risk calculator, as described in the Methods section.

### Threshold and nonlinear analysis of CKMAI

RCS analyses revealed nonlinear associations between CKMAI and all cause mortality (*P* overall < 0.001, *P* nonlinear = 0.001; S10 Fig), cardiovascular mortality (*P* overall < 0.001, *P* nonlinear = 0.002; S11 Fig), and high risk CKM status (*P* overall < 0.001, *P* nonlinear = 0.020; S12 Fig). To further quantify the threshold effects, two piecewise linear regression models were fitted (Table 3). For all cause mortality, an inflection point was identified at CKMAI = 61.587. Below this threshold, each 1 unit increase in CKMAI was associated with a HR of 1.11 (95% CI [1.10, 1.12]); above the threshold, the HR was 1.07 (95% CI [1.05, 1.09]). The likelihood ratio test comparing the piecewise model with a standard linear model was significant (*P* < 0.001), confirming a threshold effect. Similarly, for cardiovascular mortality, the inflection point was 60.732, with HRs of 1.12 (95% CI [1.10, 1.15]) below and 1.07 (95% CI [1.04, 1.10]) above the threshold (P for likelihood ratio test = 0.018). For high risk CKM status (logistic model), the inflection point was 34.092; the OR below the threshold was 1.10 (95% CI [1.06, 1.14]) and above the threshold was 1.06 (95% CI [1.05, 1.06]) (*P* for likelihood ratio test = 0.041).

**Table 3:** Threshold effect analysis of CKMAI on outcomes.

Outcome	Model	Effect (HR/OR)	95% CI	*P* value	Inflection point	P for likelihood ratio test
All-cause mortality	Standard linear regression (per 1-unit)	1.09	1.09,1.10	<0.001	–	–
	Two-piecewise linear: CKMAI < 61.587	1.11	1.10,1.12	<0.001	61.587	<0.001
	Two-piecewise linear: CKMAI ≥ 61.587	1.07	1.05,1.09	<0.001		
Cardiovascular mortality	Standard linear regression (per 1-unit)	1.10	1.09,1.11	<0.001	–	–
	Two-piecewise linear: CKMAI < 60.732	1.12	1.10,1.15	<0.001	60.732	0.018
	Two-piecewise linear: CKMAI ≥ 60.732	1.07	1.04,1.10	<0.001		
High-risk CKM status	Standard logistic regression (per 1-unit)	1.06	1.06,1.07	<0.001	–	–
	Two-piecewise logistic: CKMAI < 34.092	1.10	1.06,1.14	<0.001	34.092	0.041
	Two-piecewise logistic: CKMAI ≥ 34.092	1.06	1.05,1.06	<0.001		

Abbreviations: HR, hazard ratio; OR, odds ratio; CI, confidence interval; CKM, cardiovascular kidney metabolic. The standard linear/logistic models report the effect per 1 unit increase in CKMAI. Two piecewise models were fitted using the segmented package in R, with the inflection point estimated by the package. The likelihood ratio test compared the piecewise model with a standard linear/logistic model to determine whether a threshold effect was present; the test statistic follows a χ² distribution with degrees of freedom equal to the difference in parameters between the two models. All estimates are from fully adjusted models (Model 3: adjusted for age, sex, ethnicity, education, marital status, poverty to income ratio, smoking, drinking, physical activity, hypertension, diabetes, and cancer).

To visualize the prognostic impact of these thresholds, weighted Kaplan–Meier curves were constructed for all‑cause and cardiovascular mortality, and a weighted risk plot was generated for high‑risk CKM status (S13–S15 Figs). Participants with CKMAI above the respective thresholds had significantly lower survival probabilities (log‑rank *P* < 0.001 for both mortality outcomes) and a consistently higher risk of being in CKM stage 3 or 4.

### Stepwise adjusted associations

To assess the independent associations of CKMAI, PhenoAge, KDM, and CMI with the three outcomes, we constructed three hierarchical Cox or logistic regression models: Model 1 (unadjusted), Model 2 (adjusted for age and sex), and Model 3 (fully adjusted for age, sex, ethnicity, education, marital status, poverty-to-income ratio, smoking, drinking, physical activity, hypertension, diabetes, and cancer). Table 4 summarizes the effect estimates for all indicators across the three adjustment levels.

**Table 4:** Stepwise adjusted associations of CKMAI, PhenoAge, KDM, and CMI with all‑cause mortality, cardiovascular mortality, and high‑risk CKM status.

Outcome	Indicator	Model 1 (unadjusted)	Model 2 (age + sex)	Model 3 (fully adjusted)
**All-cause mortality** (HR, 95% CI)	CKMAI	1.10 (1.09,1.11)***	1.06 (1.04,1.07)***	1.08 (1.07,1.10)***
	PhenoAge	1.08 (1.07,1.09)***	1.04 (1.03,1.04)***	1.03 (1.02,1.04)***
	KDM	1.04 (1.04,1.05)***	1.01 (1.01,1.02)***	1.01 (1.01,1.02)***
	CMI	1.00 (0.97,1.03)	1.03 (0.99,1.06)	0.97 (0.92,1.02)
**Cardiovascular mortality** (HR, 95% CI)	CKMAI	1.11 (1.10,1.12)***	1.04 (1.01,1.07)**	1.06 (1.03,1.09)***
	PhenoAge	1.09 (1.08,1.10)***	1.03 (1.02,1.05)***	1.03 (1.01,1.04)**
	KDM	1.05 (1.04,1.05)***	1.01 (1.01,1.02)*	1.01 (1.00,1.02)
	CMI	0.99 (0.94,1.05)	1.03 (0.97,1.10)	0.99 (0.91,1.08)
**High-risk CKM status** (OR, 95% CI)	CKMAI	1.06 (1.06,1.07)***	1.04 (1.03,1.05)***	1.04 (1.02,1.06)***
	PhenoAge	1.05 (1.05,1.06)***	1.03 (1.02,1.04)***	1.01 (1.01,1.03)*
	KDM	1.04 (1.04,1.05)***	1.03 (1.02,1.03)***	1.01 (1.01,1.01)*
	CMI	1.04 (1.01,1.05)*	1.03 (1.02,1.05)*	1.01 (0.99,1.02)

Abbreviations: CKMAI, CKM specific aging index; KDM, KDM biological age; CMI, cardiometabolic index; HR, hazard ratio; OR, odds ratio; CI, confidence interval. Model 1 is unadjusted. Model 2 is adjusted for age and sex. Model 3 is fully adjusted for age, sex, ethnicity, education, marital status, poverty to income ratio, smoking, drinking, physical activity, hypertension, diabetes, and cancer. *P* values were derived from Wald tests for Cox regression (mortality outcomes) and logistic regression (CKM high risk status). ***P < 0.001; **P < 0.01; *P < 0.05.

For all cause mortality, CKMAI remained significantly associated with increased risk across all three models, with a fully adjusted HR of 1.08 (95% CI [1.07, 1.10]; *P* < 0.001). PhenoAge showed a similar pattern but with a smaller effect size (Model 3 HR 1.03, 95% CI [1.02, 1.04]; *P* < 0.001), whereas KDM yielded a HR of 1.01 (95% CI [1.01, 1.02]; *P* < 0.001). CMI was not significantly associated with all cause mortality in any model (Model 3 HR 0.97, 95% CI [0.92, 1.02]; *P* = 0.260). For cardiovascular mortality, CKMAI demonstrated a consistent positive association (Model 3 HR 1.06, 95% CI [1.03, 1.09]; *P* < 0.001). PhenoAge remained significant in Model 3 (HR 1.03, 95% CI [1.01, 1.04]; *P* = 0.002), while KDM lost significance after full adjustment (HR 1.01, 95% CI [1.00, 1.02]; *P* = 0.225). CMI was not significant in any model. For high risk CKM status (logistic model), CKMAI was the only indicator that remained statistically significant in Model 3 (OR 1.04, 95% CI [1.02, 1.06]; *P* < 0.001), whereas PhenoAge and KDM showed borderline significance (*P* = 0.012 and *P* = 0.032, respectively) with effect sizes close to 1.01, and CMI was not significant (OR 1.01, 95% CI [0.99, 1.02]; *P* = 0.082).

To assess whether the CKMAI captures aging-related information independent of chronological age, we examined the association of CKMAI_advance (CKMAI − Age) with all cause mortality, cardiovascular mortality, and high risk CKM status. After full adjustment for age, sex, and all prespecified covariates, each 1 year increase in CKMAI_advance remained significantly associated with higher risk of all cause mortality (HR 1.08, 95% CI [1.07, 1.10]), cardiovascular mortality (HR 1.06, 95% CI [1.03, 1.09]), and high risk CKM status (OR 1.03, 95% CI [1.01, 1.05]) (S15 Table). These findings demonstrate that CKMAI captures aging-related signals that extend beyond chronological age, supporting its validity as an aging metric rather than merely a disease specific score.

### Stratified analysis by CKM stage

To examine whether the associations of CKMAI, PhenoAge, KDM, and CMI with mortality vary across CKM stages, we performed stratified Cox regression analyses by CKM stage (0–1, 2, 3–4). Table 5 presents HRs for all‑cause and cardiovascular mortality for each indicator within each stratum. For all cause mortality, CKMAI showed the highest HRs in CKM stage 0–1 (HR 5.24, 95% CI [3.40, 8.08]) and stage 2 (HR 4.58, 95% CI [3.83, 5.48]), with a slightly lower but still substantial HR in stage 3–4 (HR 3.50, 95% CI [2.96, 4.14]). PhenoAge and KDM exhibited similar decreasing trends across stages, whereas CMI was not significantly associated with mortality in any stage (all 95% CIs included 1 except for stages 2 and 3–4 where HR < 1 but not protective). For cardiovascular mortality, the pattern was consistent: CKMAI and PhenoAge demonstrated strong associations, particularly in early CKM stages, while KDM showed weaker effects and CMI remained non‑significant. These findings indicate that CKMAI is a robust predictor of mortality across all CKM stages, with the strongest relative risk observed in early‑stage individuals. Forest plots summarizing these stratified results are provided in S16–S17 Figs.

**Table 5:** Hazard ratios (95% CI) of CKMAI, PhenoAge, KDM, and CMI for all‑cause and cardiovascular mortality stratified by CKM stage.

Outcome	Indicator	CKM 0–1	CKM 2	CKM 3–4
**All-cause mortality**	CKMAI	5.24 (3.40,8.08)*	4.58 (3.83,5.48)*	3.50 (2.96,4.14)*
	PhenoAge	4.94 (3.33,7.33)*	3.90 (3.31,4.60)*	3.35 (2.86,3.92)*
	KDM	5.64 (3.63,8.74)*	2.37 (2.12,2.64)*	1.66 (1.47,1.88)*
	CMI	2.99 (0.72,12.48)	0.74 (0.61,0.89)*	0.87 (0.77,0.98)*
**Cardiovascular mortality**	CKMAI	5.56 (3.15,9.80)*	6.26 (4.66,8.42)*	3.60 (2.82,4.61)*
	PhenoAge	6.25 (3.11,12.56)*	5.20 (3.98,6.80)*	3.72 (2.93,4.72)*
	KDM	10.41 (5.98,18.11)*	2.70 (2.29,3.17)*	1.64 (1.32,2.03)*
	CMI	20.86 (3.64,119.55)*a	0.79 (0.54,1.18)	0.75 (0.55,1.02)

Abbreviations: HR, hazard ratio; CI, confidence interval; CKM, cardiovascular kidney metabolic; CMI, cardiometabolic index. Models were adjusted for age, sex, ethnicity, education, marital status, poverty to income ratio, smoking, drinking, physical activity, hypertension, diabetes, and cancer. Asterisk (*) indicates that the 95% confidence interval does not include 1, denoting a statistically significant hazard ratio. *P* values were derived from Wald tests in weighted Cox regression models. *a* Wide confidence interval due to small number of cardiovascular deaths in CKM stage 0–1.

### Additive interactions

To evaluate whether CKMAI and existing biological age measures have synergistic effects beyond their individual contributions, we quantified additive interactions using the relative excess RERI and AP. Table 6 presents the RERI and AP estimates with 95% CIs for all‑cause mortality, cardiovascular mortality, and high‑risk CKM status. For all cause mortality, CKMAI and PhenoAge exhibited a significant super additive interaction (RERI 15.06, 95% CI [8.14, 38.44]; AP 0.86, 95% CI [0.67, 1.00]). A similar but smaller interaction was observed for CKMAI and KDM (RERI 10.21, 95% CI [4.82, 31.43]; AP 0.60, 95% CI [0.41, 0.78]). In contrast, the combination of CKMAI and CMI did not reach statistical significance (RERI 1.37, 95% CI [−5.46, 8.53]). For cardiovascular mortality, the additive interactions were even more pronounced for PhenoAge (RERI 40.81, 95% CI [21.72, 116.14]; AP 0.85, 95% CI [0.70, 0.97]) and KDM (RERI 25.06, 95% CI [11.54, 90.44]; AP 0.62, 95% CI [0.40, 0.78]), whereas CMI again showed no evidence of interaction (RERI −3.79, 95% CI [−28.53, 9.47]). For high risk CKM status (logistic model), CKMAI and PhenoAge demonstrated a significant additive interaction (RERI 3.09, 95% CI [1.38, 4.72]; AP 0.42, 95% CI [0.19, 0.58]). An interaction was also observed for CKMAI and CMI (RERI 2.98, 95% CI [1.51, 4.74]; AP 0.33, 95% CI [0.19, 0.46]), while the interaction with KDM was not significant (RERI 0.65, 95% CI [−1.18, 2.29]). The wide confidence interval for the cardiovascular mortality interaction (RERI 40.81, 95% CI [21.72, 116.14]) reflects the limited number of cardiovascular deaths (*n* = 201) and the resulting reduced statistical precision. Importantly, the lower bound of the 95% CI remains well above zero, indicating a statistically significant synergistic effect despite the wide interval. Detailed collinearity assessment (VIF) is provided in S20 Table. The joint forest plots for each outcome are provided in S18–S20 Figs.

**Table 6:** Additive interactions between CKMAI and universal biological age measures (PhenoAge, KDM, CMI) for all‑cause mortality, cardiovascular mortality, and high‑risk CKM status.

Outcome	Indicator	RERI (95% CI)	AP (95% CI)
**All-cause mortality**	PhenoAge	15.06 (8.14,38.44)*	0.86 (0.67,1.00)*
	KDM	10.21 (4.82,31.43)*	0.60 (0.41,0.78)*
	CMI	1.37 (–5.46,8.53)	0.08 (–0.28,0.37)
**Cardiovascular mortality**	PhenoAge	40.81 (21.72,116.14)*	0.85 (0.70,0.97)*
	KDM	25.06 (11.54,90.44)*	0.62 (0.40,0.78)*
	CMI	–3.79 (–28.53,9.47)	–0.14 (–0.65,0.22)
**High-risk CKM status**	PhenoAge	3.09 (1.38,4.72)*	0.42 (0.19,0.58)*
	KDM	0.65 (–1.18,2.29)	0.08 (–0.15,0.26)
	CMI	2.98 (1.51,4.74)*	0.33 (0.19,0.46)*

Abbreviations: RERI, relative excess risk due to interaction; AP, attributable proportion; CI, confidence interval; CKM, cardiovascular kidney metabolic; CMI, cardiometabolic index. RERI and AP were calculated using the epiR package in R. Asterisk (*) indicates that the 95% confidence interval does not include 0, denoting a statistically significant additive interaction. 95% CIs were estimated using 200 bootstrap resamples. For high risk CKM status, the model is logistic regression; for mortality outcomes, the model is Cox regression. All models were adjusted for age, sex, ethnicity, education, marital status, poverty to income ratio, smoking, drinking, physical activity, hypertension, diabetes, and cancer. *P* values for interaction were derived from Wald tests.

### Clustering analysis

Given that CKMAI exhibited the strongest additive interaction with PhenoAge, we selected PhenoAge together with the other five highest‑weighted variables (age, RDW, MCV, albumin, and ALP) to perform unsupervised clustering. Using Gaussian mixture models, we aimed to identify distinct aging‑metabolic phenotypes within the CKM syndrome continuum. The optimal number of clusters was determined as six by the Bayesian information criterion and silhouette coefficient. Table 7 summarizes the clinical characteristics of each cluster and their associated risks for all‑cause mortality, cardiovascular mortality, and high‑risk CKM status (CKM stage ≥3).

**Table 7:** Characteristics and risks of six aging‑metabolic phenotypes identified by clustering.

Cluster	Phenotype description	Age (years)	RDW (%)	MCV (fL)	Albumin (g/dL)	ALP (U/L)	PhenoAge	All-cause HR (95% CI)*	Cardiovascular HR (95% CI)*	High-risk CKM proportion (%) (95% CI)
1	Middle-aged, mild high RDW, normal ALB (mild inflammation)	43.7 (0.28)	12.98 (0.03)	89.58 (0.13)	4.25 (0.01)	57.81 (0.38)	55.29 (0.57)	1.00 (ref)	1.00 (ref)	11.6 (9.5,13.7)
2	Elderly, high RDW, low ALB, high ALP (significant aging + malnutrition)	71.7 (0.22)	13.33 (0.04)	91.01 (0.15)	4.17 (0.01)	61.29 (0.51)	79.59 (0.37)	11.74 (7.85,17.57)	30.34 (16.38,56.20)	29.1 (25.9,32.3)
3	Young,optimal indicators (healthy phenotype)	26.3 (0.16)	12.67 (0.03)	88.39 (0.14)	4.36 (0.01)	66.56 (0.63)	37.73 (0.51)	0.14 (0.04,0.49)	0.07 (0.01,0.54)	1.7 (0.9,2.4)
4	Middle-aged, high MCV, high ALP (biliary/metabolic abnormality)	55.0 (0.30)	12.79 (0.02)	90.89 (0.13)	4.26 (0.01)	77.38 (0.58)	59.64 (0.36)	2.81 (1.80,4.39)	5.48 (2.75,10.91)	14.5 (12.3,16.7)
5	Middle-aged, high RDW, low ALB, very high ALP (severe liver/bone metabolic disorder)	50.4 (1.01)	13.75 (0.06)	90.96 (0.35)	3.97 (0.03)	104.35 (2.64)	73.29 (1.17)	7.11 (4.14,12.22)	12.62 (4.86,32.76)	22.6 (16.4,28.8)
6	Middle-aged, very high RDW, very low MCV, low ALB (microcytic anemia + inflammation)	46.7 (1.02)	15.58 (0.07)	80.27 (0.26)	3.98 (0.02)	74.80 (1.39)	63.82 (1.07)	4.78 (2.89,7.91)	11.46 (5.73,22.91)	15.6 (11.9,19.3)

Data are mean (standard error) for continuous variables; hazard ratios (95% CI) for mortality with cluster 1 as reference; proportion (95% CI) for high risk CKM status. Clustering was performed using Gaussian mixture models with the mclust package in R; the optimal number of clusters was determined by the Bayesian information criterion (BIC) and silhouette coefficient. RDW, red cell distribution width; MCV, mean corpuscular volume; ALP, alkaline phosphatase; HR, hazard ratio; CKM, cardiovascular kidney metabolic. Mortality HRs were derived from weighted Cox regression models adjusted for age, sex, ethnicity, education, marital status, poverty to income ratio, smoking, drinking, physical activity, hypertension, diabetes, and cancer. *P* values for HRs were derived from Wald tests.

Cluster 3 (young healthy) had the youngest mean age (26.3 years) and lowest PhenoAge (37.7), with the most favorable biomarker profile (lowest RDW, highest albumin). In contrast, cluster 2 (frail elderly) comprised the oldest individuals (mean age 71.7 years), highest PhenoAge (79.6), and elevated RDW and ALP, together with lower albumin. Clusters 5 and 6 represented intermediate‑risk phenotypes: cluster 5 was characterized by markedly elevated ALP (104.4 U/L) and low albumin, while cluster 6 showed extremely high RDW (15.6%) and low MCV (80.3 fL), suggesting an anemia‑inflammation phenotype.

Compared with cluster 1 (reference), cluster 3 had significantly lower risks for all outcomes (all cause mortality HR 0.14, 95% CI [0.04, 0.49]; cardiovascular mortality HR 0.07, 95% CI [0.01, 0.54]; proportion of high risk CKM 1.7%). Cluster 2 had the highest risks (all cause HR 11.74, 95% CI [7.85, 17.57]; cardiovascular HR 30.34, 95% CI [16.38, 56.20]; high risk CKM proportion 29.1%). Clusters 5 and 6 also exhibited elevated risks, with HRs ranging from 4.78 to 12.62. The Kaplan‑Meier survival curves for all‑cause and cardiovascular mortality across the six clusters are shown in S21, S22 Figs, respectively, and the proportion of high‑risk CKM status by cluster is displayed in S23 Fig. These results underscore the marked heterogeneity in aging‑metabolic phenotypes and their strong association with clinical outcomes.

### Subgroup and sensitivity analyses

We performed prespecified subgroup analyses to evaluate whether the association between CKMAI and outcomes was consistent across diverse clinical and demographic strata. Figure 5 presents a combined forest plot summarizing HRs for all‑cause mortality, cardiovascular mortality, and ORs for high‑risk CKM status across all subgroups. CKMAI was significantly associated with increased risk in every subgroup examined. For all‑cause mortality, HRs ranged from 1.07 to 1.13 (all *P* < 0.001); for cardiovascular mortality, HRs ranged from 1.07 to 1.14 (all *P* < 0.001); and for high‑risk CKM status, ORs ranged from 1.04 to 1.09 (all *P* < 0.001). Although some subgroup‑specific interaction *P* values were nominally significant (e.g., ethnicity in all‑cause mortality, *P* = 0.001; diabetes and CKD in all‑cause mortality, both *P* = 0.002), none remained significant after Benjamini‑Hochberg false discovery rate correction (all adjusted *P* > 0.05). Therefore, no meaningful effect modification was identified, indicating that CKMAI’s predictive performance is robust across different patient populations.

**Fig 5:**
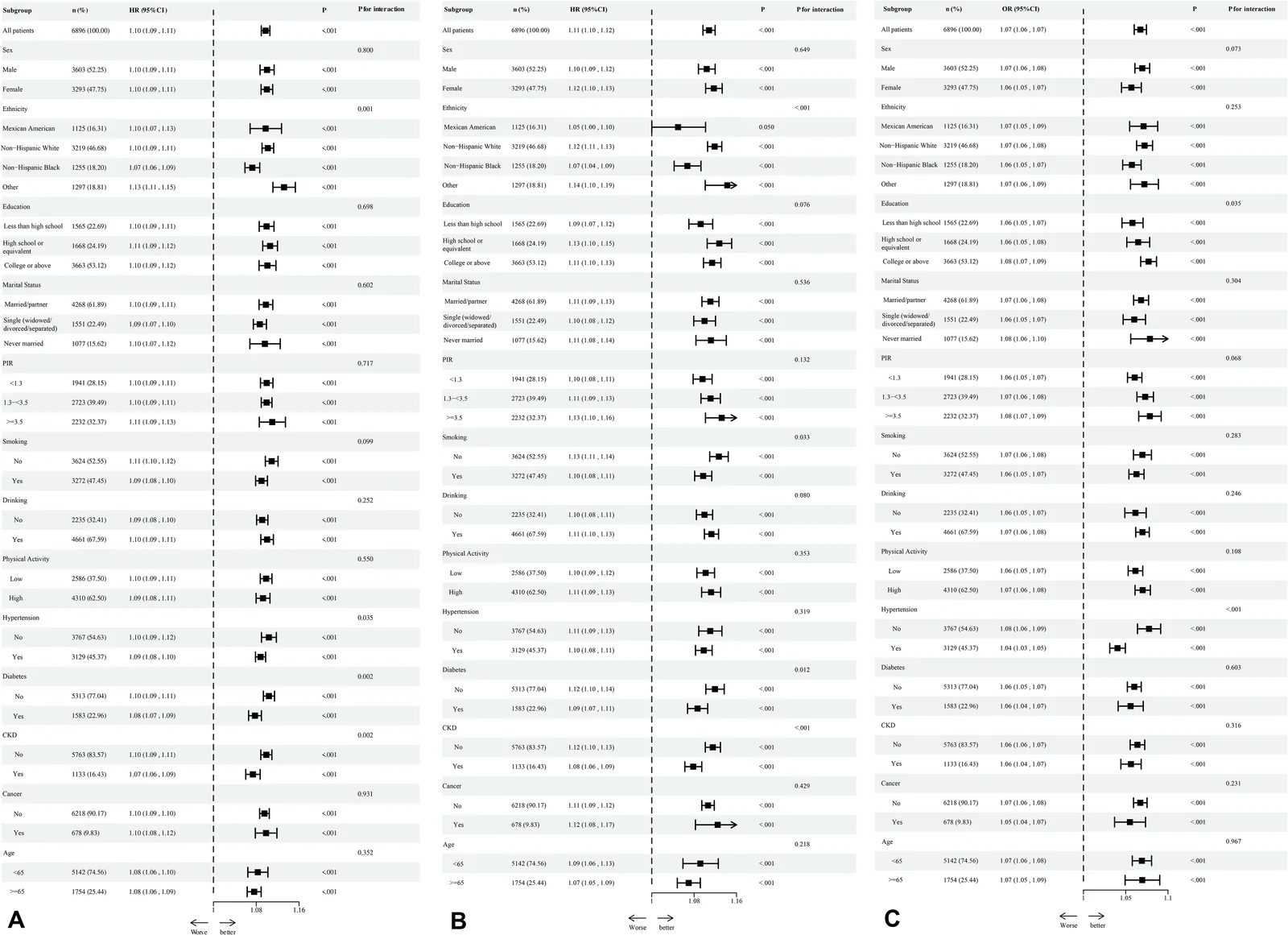
Combined forest plot of subgroup analyses for all‑cause mortality, cardiovascular mortality, and high‑risk CKM status. **Legend:** Panel A displays hazard ratios (HRs) for all cause mortality, panel B for cardiovascular mortality, and panel C for high risk CKM status (odds ratios, ORs). All estimates are shown with 95% confidence intervals. All HRs/ORs were statistically significant (*P* < 0.05). *P* values for interaction were derived from Wald tests for the interaction terms in the fully adjusted models, followed by Benjamini Hochberg false discovery rate (FDR) correction for multiple comparisons. No significant effect modification was detected after FDR correction (all adjusted P for interaction > 0.05). HR, hazard ratio; CI, confidence interval; OR, odds ratio; CKD, chronic kidney disease.

To further assess the generalizability of the CKMAI across demographic groups, we evaluated its discriminative performance (C index, time-dependent AUC, and AUC for CKM high risk status) stratified by sex and ethnicity. As shown in S25 Table, the CKMAI demonstrated consistently good discrimination across all subgroups. C index for all cause mortality ranged from 0.794 (Non-Hispanic Black) to 0.902 (Other ethnicity), and AUC for high risk CKM status ranged from 0.727 (Female) to 0.767 (Mexican American). Calibration plots (S27 Fig) showed good agreement between predicted and observed risks across all subgroups, with minor deviations observed only in the Non-Hispanic Black subgroup at extreme risk thresholds.

To further assess the robustness of our findings, we conducted several sensitivity analyses. First, unweighted Cox regression gave an HR of 1.092 (95% CI [1.088, 1.096]) for all cause mortality, closely matching the primary weighted estimate (HR 1.097) (S7 Table). Second, restricting follow-up to 5 years yielded an HR of 1.096 (95% CI [1.085, 1.108]) (S7 Table). Third, excluding participants with baseline cardiovascular disease or CKD resulted in an HR of 1.097 (95% CI [1.084, 1.110]) (S7 Table). Fourth, we compared the predictive performance of the CKMAI (single index) with that of models using the original variables directly. For all cause mortality, an 18 variable Cox model was fitted; for CKM high risk status, an 8 variable model (variables that passed stability selection for this outcome) was used. The CKMAI achieved nearly identical performance to the full 18 variable model for all cause mortality (e.g., 3 year AUC: 0.893 versus 0.889; 5 year AUC: 0.907 versus 0.907) and slightly outperformed the stability selected model for CKM high risk status (AUC 0.790 versus 0.775). Full results are provided in S16 Table. Fifth, we evaluated the discriminative ability of the CKMAI for stage 3 alone and stage 4 alone. The CKMAI demonstrated significant discrimination for both phenotypes: AUC 0.74 (95% CI [0.72, 0.93]) for stage 3 versus CKM 0–2 and AUC 0.82 (95% CI [0.80, 0.84]) for stage 4 versus CKM 0–2, with overlapping CIs supporting the validity of combining them as a unified high risk category (S19 Table). Sixth, comparison of baseline characteristics between included and excluded participants aged ≥18 years showed that the two groups were broadly similar across all evaluated variables, with standardized mean differences (SMD) ranging from −0.196 to 0.177, indicating negligible to small differences. Mortality outcomes were also comparable (all cause: SMD = 0.048; CVD: SMD = 0.023). Full results are provided in S21 Table. Across 20 imputed datasets, the pooled performance of CKMAI (mean C index: 0.851 for all cause mortality, 0.873 for cardiovascular mortality, and 0.787 for CKM high risk status) was nearly identical to the complete case analysis, and CKMAI consistently outperformed PhenoAge, KDM, and CMI across all imputed datasets. Detailed results are provided in S22, S23 Tables. Seventh, to address the competing risk of noncardiovascular death, we performed a Fine Gray subdistribution hazard model. The SHR for CKMAI (SHR 1.024, 95% CI [1.004, 1.045], *P* = 0.018) was nearly identical to the cause specific HR from the standard Cox model (HR 1.06, 95% CI [1.03, 1.09], *P* < 0.001), confirming that the association between CKMAI and cardiovascular mortality is robust and not meaningfully affected by competing risks. Detailed results are provided in S24 Table. Collectively, they confirm that the observed associations are not driven by weighting schemes, follow‑up duration, or prevalent disease at baseline, underscoring the stability of the CKMAI‑mortality relationship.

### Temporal validation and external validation

The early period (2005–2010) comprised 3,900 participants and the late period (2011–2018) comprised 2,996 participants. The two cohorts were well balanced in terms of age (mean 48.0 versus 47.7 years), sex, and ethnicity, supporting the comparability of the two temporally distinct populations. Detailed baseline characteristics are presented in S9 Table.

When the entire modeling pipeline was retrained using the early period (2005–2010) and tested on the late period (2011–2018), the CKMAI model demonstrated strong discriminative performance across all three outcomes on the late test set. For all cause mortality, the C index of CKMAI was 0.837 (95% CI [0.795, 0.878]); for cardiovascular mortality, it was 0.881 (95% CI [0.814, 0.947]); and for high risk CKM status (binary outcome), the AUC was 0.804 (95% CI [0.784, 0.825]). In comparison, PHENOAGE, KDM, and CMI yielded C indices of 0.774 (95% CI [0.729, 0.819]), 0.743 (95% CI [0.692, 0.793]), and 0.504 (95% CI [0.445, 0.564]), respectively, for all cause mortality. For cardiovascular mortality, the corresponding C indices were 0.840 (95% CI [0.779, 0.902]) for PHENOAGE, 0.803 (95% CI [0.727, 0.880]) for KDM, and 0.587 (95% CI [0.473, 0.702]) for CMI. For high risk CKM status, the AUCs were 0.758 (95% CI [0.736, 0.781]) for PHENOAGE, 0.734 (95% CI [0.709, 0.758]) for KDM, and 0.611 (95% CI [0.583, 0.638]) for CMI. For high risk CKM status, the AUCs were 0.758 (0.736–0.781) for PHENOAGE, 0.734 (0.709–0.758) for KDM, and 0.611 (0.583–0.638) for CMI. Across all three outcomes, CKMAI consistently exhibited the highest discriminative ability, substantially outperforming the other aging and cardiometabolic indices (S10 Table and S24 Fig).

Using the original CKMAI weights and standardization parameters applied to both time periods, the CKMAI demonstrated consistent performance across early and late NHANES cycles (S11 Table and S25 Fig). For all cause mortality, the C index was 0.842 (95% CI [0.827, 0.854]) in the early period and 0.808 (95% CI [0.755, 0.853]) in the late period, with corresponding 3 year AUCs of 0.869 (95% CI [0.842, 0.900]) and 0.816 (95% CI [0.766, 0.859]). For cardiovascular mortality, the C indices were 0.854 (95% CI [0.828, 0.875]) and 0.881 (95% CI [0.826, 0.927]), and for high risk CKM status, the AUCs were 0.755 (95% CI [0.736, 0.775]) and 0.775 (95% CI [0.753, 0.798]). When the early data were re standardized using late period means and SDs, and the late data using early period means and SDs, the performance remained virtually unchanged (e.g., early data with late parameters: C index 0.843, 3 year AUC 0.870), confirming robustness against the choice of standardization reference. Across both periods and all metrics, the CKMAI consistently outperformed PhenoAge, KDM, and CMI.

In the hospital validation cohort (*n* = 261; mean age 54.0 years, 54.0% female; 44 patients [16.9%] with CKM stage ≥3), the CKMAI achieved an AUC of 0.789 (95% CI [0.730, 0.847]) for discriminating CKM high risk status (S12 Table, S26 Fig). Baseline characteristics of this cohort, stratified by CKM stage, are presented in S13 Table; normality assessment of continuous variables is provided in S14 Table. While this hospital cohort is limited by its modest size, homogeneous ethnicity (all Han Chinese), and lack of mortality follow-up, the consistent and significant discrimination of CKM high risk status across all indices supports the transferability of the CKMAI to real world clinical settings.

### Mediation analysis

To examine whether depression score mediates the association between CKMAI and the three outcomes, we performed weighted bootstrap mediation analysis with 1,000 replicates, adjusting for sex, ethnicity, education, and age (the same covariates as in Model 3). S8 Table presents ACME, ADE, total effects, and proportions mediated. For all cause mortality, depression score significantly mediated the CKMAI mortality relationship, with a proportion mediated of 5.6% (95% CI [2.3%, 9.0%]; *P* < 0.001). For cardiovascular mortality, the proportion mediated was 8.2% (95% CI [2.8%, 22.0%]; *P* < 0.001). For high risk CKM status, the mediation effect was stronger, with a proportion mediated of 11.1% (95% CI [6.7%, 24.0%]; *P* < 0.001). In all three outcomes, the direct effects (ADE) remained highly significant, indicating that depression only partially mediates the CKMAI‑outcome associations. Figure 6 provides a graphical summary of the mediation pathways and effect sizes for the three outcomes. To test the alternative direction, we performed a reverse mediation analysis with depression score as the exposure, CKMAI as the mediator, and each outcome as the dependent variable. The indirect effects (ACME) were either nonsignificant or biologically implausible (e.g., negative for all cause mortality), ruling out depression as a confounder operating through CKMAI. Additionally, a moderation analysis testing the interaction term CKMAI × Depression showed no significant interaction for any outcome (all *P* > 0.05), indicating that depression does not modify the effect of CKM specific aging. Full results are provided in S17, S18 Tables. These findings suggest that a modest but statistically significant fraction of the increased mortality and CKM progression risk associated with higher CKMAI is attributable to depressive symptomatology.

**Fig 6:**
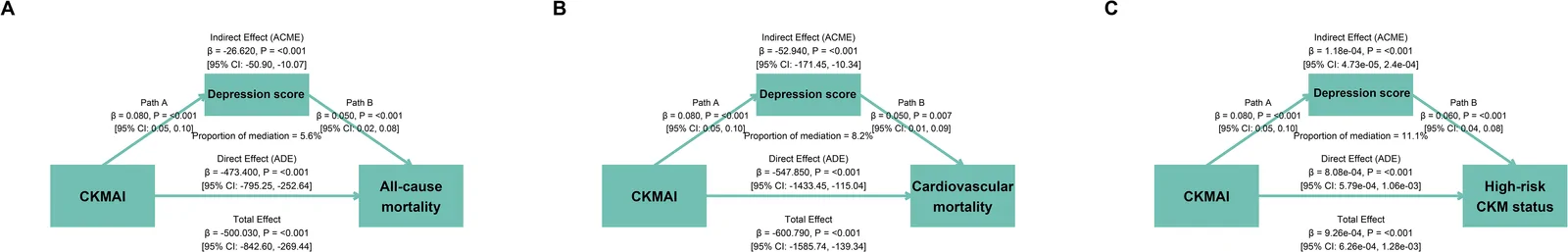
Graphical summary of mediation analysis for all‑cause mortality, cardiovascular mortality, and high‑risk CKM status. **Legend:** The figure illustrates the mediation pathways from CKMAI to outcomes through depression score. Panel A shows all cause mortality, panel B cardiovascular mortality, and panel C high risk CKM status. The indirect effect (average causal mediation effect, ACME) is shown along each pathway, with the proportion mediated indicated. The direct effect (average direct effect, ADE) and total effect are also reported. All effects were statistically significant (*P* < 0.001). *P* values and 95% CIs were derived from weighted bootstrap mediation analysis with 1,000 replicates using the mediation package in R, with the quasi Bayesian Monte Carlo method for confidence interval estimation. CKMAI, CKM specific aging index; CKM, cardiovascular kidney metabolic; ACME, average causal mediation effect; ADE, average direct effect; CI, confidence interval.

## Discussion

In this large, nationally representative cohort study of US adults, we developed and validated a CKM specific aging index using a systematic machine learning framework that integrates 18 routine clinical biomarkers across metabolic, kidney, aging, inflammatory nutritional, and psychosocial domains. CKMAI demonstrated superior discrimination for all‑cause mortality and cardiovascular mortality compared with universal biological age indices (PhenoAge, KDM) and a traditional metabolic index (CMI). It also outperformed these indices in identifying high‑risk CKM status. Importantly, CKMAI is based solely on widely available, making it immediately implementable in primary care settings, including in resource‑limited environments. The incremental value of CKMAI was further supported by significant improvements in net reclassification and integrated discrimination, as well as by consistently higher net benefit across decision curve analyses. Notably, CKMAI exhibited super‑additive interactions with PhenoAge and KDM, indicating that combining CKM‑specific and universal aging measures provides synergistic risk information. Clustering analysis uncovered six distinct aging‑metabolic phenotypes, ranging from a healthy young cluster with near‑zero excess risk to a frail elderly cluster with an 11‑fold higher all‑cause mortality hazard. Depression partially mediated the CKMAI‑outcome associations, with proportions of mediation ranging from 5.6% for all‑cause mortality to 11.1% for high‑risk CKM status, suggesting that addressing mental health could be a tangible target for intervention. The unified high risk definition (CKM stage ≥3) was chosen to prioritize early intervention; delaying action until precise sub stage differentiation (stage 3 versus 4) would risk missing the window for prevention, as both stages represent advanced disease requiring escalation of care. These findings position CKMAI as a practical, interpretable tool for risk stratification across the entire CKM continuum and highlight the importance of integrating psychosocial factors into CKM risk assessment.

While CKMAI is specialized for the CKM pathway, we contend that its framing as an aging index is justified on both biological and empirical grounds. Biologically, the biomarkers comprising CKMAI—particularly RDW (a marker of inflammation and oxidative stress), albumin (a negative acute phase reactant and nutritional indicator), and kidney function markers (eGFR, UACR)—capture systemic physiological decline that accelerates with age and is central to the aging process. Thus, CKMAI does not simply measure CKM disease status; it quantifies the biological wear and tear accumulating across the metabolic, renal, and inflammatory systems that define the CKM continuum. Empirically, we calculated CKMAI_advance (CKMAI − Age) to isolate the aging related signal independent of chronological age. In fully adjusted models, CKMAI_advance remained significantly associated with all cause mortality, cardiovascular mortality, and high risk CKM status, confirming that CKMAI captures aging related information beyond what would be expected based on chronological age alone. Importantly, we do not propose CKMAI as a replacement for universal biological age measures such as PhenoAge; rather, we view it as a complementary tool that provides CKM specific aging information, which may be particularly relevant for risk stratification and intervention in the CKM syndrome population.

Our results fill a critical gap by providing a CKM‑tailored aging index that outperforms generic measures. Recent studies have demonstrated that individual metabolic markers, such as the triglyceride‑glucose index or the atherogenic index of plasma, are associated with cardiovascular outcomes in CKM populations [35,36]. However, these indices focus on single aspects of metabolic dysregulation and do not capture the systemic nature of biological aging or kidney impairment [37]. In contrast, CKMAI’s multi‑domain design—spanning metabolic, renal, inflammatory‑nutritional, and psychosocial factors—offers a holistic reflection of the CKM pathophysiological continuum. The fact that the highest weights in CKMAI were assigned to red cell distribution width (a marker of inflammation and oxidative stress), poverty‑income ratio, and kidney function markers (eGFR, UACR, BUN, SCR) reinforces the multi‑system nature of CKM progression and highlights the potential for targeted interventions. Universal biological age measures, derived from general population mortality, miss these distinctive contributions. Thus, CKMAI not only predicts risk more accurately but also generates hypotheses about modifiable drivers of CKM progression.

From a clinical and public health perspective, CKMAI offers actionable advantages. Of note, the CKMAI was developed as a unified composite index rather than separate prediction models for each outcome. A sensitivity analysis comparing the CKMAI with models using the original variables directly showed virtually identical performance, confirming that the single index captures the shared CKM specific aging signal without sacrificing predictive accuracy. This unified approach, combined with the open source R package and interactive Shiny application, facilitates clinical translation by providing a single, interpretable score analogous to “heart age” or “PhenoAge,” whereas multiple risk scores could create confusion in clinical decision-making. In early CKM stages (0‑2), where metabolic abnormalities are often subclinical but potentially modifiable, CKMAI could help identify individuals who would benefit from intensive lifestyle interventions or earlier pharmacological therapy. For patients in advanced stages (3‑4), CKMAI may refine prognostication and guide the intensity of follow‑up and treatment. The availability of a freely accessible web-based risk calculator (https://zhuzhengyang.shinyapps.io/shiny/) and an open-source R package (CKMAIcalc, https://github.com/zhuzhengyang517/CKMAIcalc) lowers the barrier to clinical implementation, enabling rapid risk estimation at the point of care without specialized software and supporting large scale epidemiological analyses and integration into automated workflows. This directly responds to the American Heart Association’s call for practical tools that translate CKM health metrics into actionable clinical decisions and has the potential to improve cardiovascular-kidney metabolic health equity by democratizing access to precision risk assessment.

Our finding of a super‑additive interaction between CKMAI and PhenoAge is novel and clinically actionable. It suggests that the combined assessment of CKM‑specific aging (as captured by CKMAI) and universal biological aging (as captured by PhenoAge) yields risk estimates that exceed the sum of their individual effects. The wide confidence interval for cardiovascular mortality reflects the limited number of cardiovascular events (*n* = 201) and should be interpreted with appropriate caution. Nevertheless, the point estimate (40.81) with a lower bound well above zero supports a genuine synergistic effect. Mechanistically, CKMAI and PhenoAge, despite sharing some components, capture distinct aging domains—CKM specific versus systemic—and their combined elevation may expose patients to converging inflammatory and metabolic pathways, leading to risk exceeding the sum of each alone. This finding is hypothesis generating and warrants replication in larger cohorts. This pattern has been observed in other contexts, such as the combined effect of smoking and CKM stage on cardiovascular risk [38], and our findings extend this observation to aging indices. Mechanistically, CKMAI may capture organ‑specific damage (particularly in the kidney and metabolic systems), whereas PhenoAge represents systemic physiological decline. When both are elevated, patients are exposed to two independent but synergistic pathways of risk. From a clinical standpoint, this finding encourages the simultaneous use of both indices in high‑risk populations (e.g., stage 2 or stage 3 CKM patients) to refine prognostication and guide more intensive interventions. For example, a patient with moderately elevated CKMAI but normal PhenoAge might benefit from targeted kidney‑protective measures, whereas a patient with both indices elevated would warrant comprehensive cardiorenal‑metabolic management.

The threshold effects identified for CKMAI—with inflection points at 61.6 for all‑cause mortality, 60.7 for cardiovascular mortality, and 34.1 for high‑risk CKM status—provide simple, memorable cut‑offs for clinical decision‑making. Below these thresholds, the risk increase per unit CKMAI was steeper, suggesting that early elevations in CKM‑specific aging are particularly harmful. Above the thresholds, the slope attenuated, possibly due to a saturation of risk or competing causes of death. Such nonlinear dose‑response relationships have been described for other metabolic risk markers in CKM populations [39,40], and our results extend this observation to a composite aging index. These thresholds could be used to define “high‑risk” CKMAI values that trigger more aggressive risk factor modification, and they can be easily communicated to patients (e.g., “a CKMAI above 60 means your risk is rising fast”).

The clustering analysis revealed six distinct aging‑metabolic phenotypes, which directly support personalized medicine for CKM syndrome. While Yang and colleagues identified five clusters in a UK Biobank CKM population using a different set of clinical biomarkers [41]. Our clusters expand this framework by including an anemia‑inflammation phenotype (cluster 6) and a very high‑ALP phenotype (cluster 5) that may indicate liver‑bone‑metabolic dysfunction. The marked gradient in mortality risk across clusters—from a HR of 0.14 in the healthy young cluster to 11.74 in the frail elderly cluster—demonstrates the substantial heterogeneity that exists within the CKM syndrome spectrum, even after accounting for chronological age. These phenotypes offer a roadmap for targeted interventions: patients in cluster 5 (high ALP, low albumin) might benefit from evaluation for liver or bone disease; those in cluster 6 (high RDW, low MCV) warrant work‑up for nutritional deficiencies or chronic inflammation; and cluster 2 (frail elderly) might benefit from comprehensive geriatric assessment, including fall prevention, nutritional support, and deprescribing. The identification of these phenotypes could also inform clinical trial enrichment strategies, ensuring that novel therapies are tested in populations most likely to respond.

Critically, the performance of the CKMAI across demographic subgroups has direct implications for health equity. In stratified analyses by sex and ethnicity, the CKMAI demonstrated generally good discrimination across most groups; however, the observed calibration in the Non‑Hispanic Black subgroup showed minor deviations at extreme risk thresholds, suggesting that the model may require additional refinement or validation in this population. This is particularly relevant given the well‑documented disparities in CKM syndrome burden—racial and ethnic minority groups and women often experience higher rates of metabolic risk factors, delayed diagnosis, and poorer cardiovascular outcomes. Despite these observed differences, the CKMAI maintained adequate overall performance across sexes and racial/ethnic groups, and its use may help avoid the pitfalls of “one‑size‑fits‑all” risk tools that could inadvertently perpetuate existing inequities through differential measurement error or misclassification. However, these subgroup findings should be interpreted with caution, as the analyses were exploratory and may have been underpowered to detect subtle differences in performance. The CKMAI provides a standardized, objective metric that can be applied across diverse patient populations, but routine validation of prediction models across demographic strata remains essential to ensure fairness and clinical utility for all populations, particularly in groups where performance was less optimal.

Our mediation analysis provides quantitative evidence that depressive symptomatology partially explains the association between CKM-specific aging and adverse outcomes. The proportion mediated was modest (5.6%–11.1%) but statistically significant, consistent with recent studies showing that depression accelerates CKM progression and increases mortality risk [42,43]. The larger mediation effect for high‑risk CKM status (11.1%) than for mortality (5.6%–8.2%) suggests that depression may exert its strongest influence during the transition from early to advanced CKM stages [44]. This is a clinically important window: interventions targeting depression—such as cognitive behavioral therapy, physical activity, or pharmacotherapy—could potentially slow CKM progression and reduce the burden of cardiovascular‑kidney‑metabolic disease. The effect is likely mediated through both behavioral pathways (reduced adherence to lifestyle and medication) and biological pathways (chronic low‑grade inflammation, autonomic dysfunction, and activation of the hypothalamic‑pituitary‑adrenal axis) [45,46]. These results highlight the importance of integrating mental health screening and intervention into routine CKM care, particularly in patients with elevated CKMAI who are still in early stages where prevention may be most effective.

Beyond its superior predictive accuracy, CKMAI offers several practical advantages that enhance its clinical utility and potential for widespread adoption. First, the laboratory components of the CKMAI are routinely collected in standard clinical practice, and the nutritional and psychosocial variables can be efficiently obtained from medical records or brief patient questionnaires, making the CKMAI readily calculable without specialized assays or extensive additional patient visits. This contrasts sharply with omics‑based aging clocks that require proteomic or epigenetic profiling, which remain too costly and technically demanding for routine use [47,48]. By leveraging existing laboratory data, CKMAI represents a cost‑effective, scalable solution for CKM risk stratification, particularly in low‑resource settings where access to advanced testing is limited. Second, the online risk calculator enables real‑time risk estimation, empowering clinicians to incorporate CKMAI into shared decision‑making with patients. For instance, a middle‑aged adult with stage 1 CKM (excess adiposity only) and a CKMAI value above 60 could be counseled about their substantially elevated mortality risk and offered more intensive lifestyle or pharmacologic interventions, whereas a patient with stage 2 CKM but CKMAI below 40 might be reassured and managed with standard guideline‑directed care. Third, the linear transformation of CKMAI to a 0‑100 scale provides an intuitive, patient‑friendly metric analogous to well‑known cardiovascular risk scores, facilitating communication and uptake.

How does CKMAI compare with existing CKM risk prediction tools? The PREVENT equations, recently endorsed by the American Heart Association, estimate 10‑year and 30‑year cardiovascular risk using traditional risk factors and kidney function, but they do not incorporate inflammatory, nutritional, or psychosocial domains [49]. However, a formal quantitative comparison between CKMAI and PREVENT was not performed, as the two tools serve distinct purposes: PREVENT predicts 10 year incident cardiovascular events in the general population, whereas CKMAI is specifically optimized for all cause and cardiovascular mortality and high risk CKM status in the CKM syndrome continuum. The different outcome definitions and time scales make direct comparison of discrimination metrics methodologically challenging. Nevertheless, the CKMAI complements existing tools by capturing the accelerated biological aging that accompanies CKM progression, offering prognostic information that is independent of and additive to traditional risk factors. The CKM2S2‑BAG score, another recently proposed tool, uses cholesterol, kidney function, sex, smoking, blood pressure, age, and glucose to predict cardiovascular risk but again omits markers of biological aging and depression [50]. CKMAI complements these tools by capturing the accelerated biological aging that accompanies CKM progression, offering prognostic information that is independent of and additive to traditional risk factors. Moreover, the super‑additive interaction between CKMAI and PhenoAge suggests that using CKMAI alone might be sufficient in many settings, as it already integrates universal aging information, but the combination provides maximal risk discrimination for the highest‑risk patients.

From a mechanistic standpoint, the composition of CKMAI provides clues about the drivers of CKM progression. The prominence of RDW, a marker of erythrocyte heterogeneity that rises with inflammation, oxidative stress, and nutritional deficiencies, underscores the role of chronic low‑grade inflammation as a unifying mechanism across the CKM spectrum [51]. The inclusion of kidney function markers (eGFR, UACR, BUN, and SCR) reflects the bidirectional relationship between metabolic dysfunction and renal impairment—a vicious cycle that accelerates both aging and organ failure [52]. The contribution of albumin, a negative acute‑phase reactant and marker of nutritional status, points to the importance of protein‑energy wasting, particularly in advanced CKM stages where cachexia and frailty become prevalent [53]. Social determinants captured by PIR and education highlight that CKM risk is not purely biological; socioeconomic disadvantage amplifies the burden of metabolic and kidney disease, likely through barriers to healthy lifestyle, medication access, and healthcare utilization [54,55]. Thus, CKMAI serves not only as a prognostic index but also as a hypothesis‑generating tool: each component may represent a modifiable target for intervention, from anti‑inflammatory strategies to nutritional support to social programs addressing poverty and education.

The clustering analysis, by identifying six distinct phenotypes, offers a roadmap for personalized medicine in CKM syndrome, moving beyond a one‑size‑fits‑all staging approach. For example, cluster 5 (high ALP, low albumin) may indicate underlying metabolic bone disease or cholestatic liver dysfunction, which could be further evaluated and treated with vitamin D, calcium, or ursodeoxycholic acid. Cluster 6 (high RDW, low MCV) suggests iron deficiency anemia or chronic disease anemia, warranting iron studies and potentially iron replacement. Cluster 2 (frail elderly) might benefit from comprehensive geriatric assessment, including fall prevention, nutritional support, and deprescribing. By enabling phenotype‑guided care, CKMAI has the potential to improve patient outcomes and reduce unnecessary healthcare utilization.

Several limitations warrant consideration. First, the observational design precludes causal inference; residual confounding cannot be excluded. Second, complete‑case analysis after exclusions could introduce selection bias, although the final cohort mirrored the full NHANES population. Third, while we have performed temporal validation within NHANES (training on 2005–2010 and testing on 2011–2018) and a preliminary external validation in a hospital-based Chinese cohort, the hospital validation cohort was modest in size, ethnically homogeneous (all participants were of Han Chinese ethnicity), and lacked mortality follow-up. Thus, the generalizability of the CKMAI to other populations and its performance for hard clinical endpoints in external settings remain to be confirmed in larger, more diverse cohorts with complete outcome ascertainment [56]. Fourth, the mediation proportion for depression was modest; larger effects might be observed with repeated measures. Fifth, kidney‑related deaths were too few (*n* = 10) to allow robust analysis. Sixth, biomarkers were measured at a single time point, precluding assessment of dynamic changes. Seventh, self‑reported data for some comorbidities and lifestyle factors may introduce misclassification bias, though sensitivity analyses suggest robustness.

Several avenues for future research emerge from this work. Prospective cohort studies with serial measurements of CKMAI could investigate whether changes in CKMAI predict clinical outcomes and whether interventions that lower CKMAI (e.g., SGLT2 inhibitors, GLP‑1 receptor agonists, lifestyle modification) translate into reduced mortality [57,58]. The role of CKMAI in guiding treatment decisions—for example, whether patients with high CKMAI derive greater absolute benefit from intensive blood pressure control, lipid lowering, or newer cardiorenal protective drugs—should be tested in post‑hoc analyses of randomized trials. Additionally, integrating CKMAI into electronic health records with automated risk calculation could facilitate population health management, enabling health systems to identify high‑risk individuals and target resources efficiently. Finally, expanding CKMAI to include emerging biomarkers such as biomarkers of senescence (p16INK4a, p21), circulating mitochondrial DNA, or proteomic signatures might further refine its predictive accuracy and mechanistic insights [59].

CKMAI is a robust, clinically implementable, and cost‑effective CKM‑specific aging index that outperforms universal biological age measures in predicting all‑cause and cardiovascular mortality and high‑risk CKM status using only routine laboratory tests. Its super‑additive interaction with PhenoAge, the identification of six distinct aging‑metabolic phenotypes, and the partial mediation by depression provide new insights into the heterogeneous progression of CKM syndrome and offer actionable targets for intervention—from combined aging assessments to phenotype‑guided care and depression screening. By integrating a diverse set of clinical laboratory measures, nutritional assessments, lifestyle factors, and social determinants of health, CKMAI provides a practical, scalable tool for risk stratification, personalized management, and clinical trial enrichment that can be implemented immediately in primary care and resource‑limited settings. Future efforts should prioritize external validation in diverse cohorts, implementation science to support widespread adoption, and randomized trials testing whether CKMAI‑guided interventions improve patient outcomes.

### Institutional Review Board statement

The National Health and Nutrition Examination Survey (NHANES) protocol was approved by the National Center for Health Statistics (NCHS) Research Ethics Review Board (Protocol #2011-17). All NHANES participants provided written informed consent. The present study is a secondary analysis of de‑identified, publicly available NHANES data and was exempt from additional institutional review board approval. For the hospital‑based validation cohort, the study was conducted in accordance with the Declaration of Helsinki. The requirement for formal ethics approval and written informed consent was waived by the Medical Ethics Committee of the First Affiliated Hospital of Anhui University of Chinese Medicine for this retrospective study utilizing fully anonymized medical records. The study was determined to be exempt from full ethics review based on its retrospective, non‑interventional design, the use of de‑identified data, and the minimal risk posed to participants, in accordance with Chinese regulations for research using anonymized clinical data. All patient data were anonymized prior to analysis, and patient privacy was protected throughout the study.

## Supporting information

S1 Checklist STROBE Statement. Checklist of items that should be included in reports of observational studies. The STROBE checklist is reproduced from the STROBE Statement (https://www.strobe-statement.org/), and is licensed under a Creative Commons Attribution 4.0 International (CC BY 4.0) license.
(DOCX)

S2 Checklist TRIPOD‑AI Statement. Transparent reporting of a multivariable prediction model for individual prognosis or diagnosis. This checklist is reproduced from Collins GS, and colleagues. TRIPOD+AI statement: updated guidance for reporting clinical prediction models that use regression or machine learning methods. BMJ. 2024 Apr 16;385:e078378. https://doi.org/10.1136/bmj-2023-078378, and is licensed under a Creative Commons Attribution 4.0 International (CC BY 4.0) license.
(DOCX)

S1 Protocol Analysis plan. (DOCX)

S1 Methods Supplementary methods for the hospital validation cohort. (DOCX)

S2 Methods Complete hyperparameter settings and tuning grids for all 14 feature selection strategies and 15 survival modeling approaches in the two-stage machine learning pipeline. (DOCX)

S3 Methods Operational instructions for the open-source CKMAIcalc R package, covering installation, input formatting, core function usage, and score interpretation. (DOCX)

S1 Data Minimal dataset (data.csv) containing the values behind the means, standard deviations, and other measures reported; the values used to build graphs; and the points extracted from images for analysis. (XLSX)

S1 Fig Correlation heatmap between CKMAI and biological age indices (PhenoAge, KDM, and chronological age). (TIF)

S2 Fig Correlation matrix among the 18 component variables of CKMAI, mortality outcomes, and high‑risk CKM status. (TIF)

S3 Fig Bootstrap stability selection frequencies and overlap of stable features for all‑cause mortality and CKM high‑risk status. (TIF)

S4 Fig SHAP summary plot combining feature importance (bar plot) and individual SHAP values (bee swarm plot) for all‑cause mortality predictions. (TIF)

S5 Fig Decision curve analysis for all‑cause mortality, cardiovascular mortality, and high‑risk CKM status. (TIF)

S6 Fig Calibration curves for all‑cause mortality, cardiovascular mortality, and high‑risk CKM status. (TIF)

S7 Fig Nomogram for predicting 3‑year, 5‑year, and 10‑year all‑cause mortality based on the CKMAI model. (TIF)

S8 Fig Nomogram for predicting 3‑year, 5‑year, and 10‑year cardiovascular mortality based on the CKMAI model. (TIF)

S9 Fig Nomogram for predicting high‑risk CKM status (stage ≥3) based on the CKMAI model. (TIF)

S10 Fig Restricted cubic spline curves for the association of CKMAI with all‑cause mortality. (TIF)

S11 Fig Restricted cubic spline curves for the association of CKMAI with cardiovascular mortality. (TIF)

S12 Fig Restricted cubic spline curves for the association of CKMAI with high‑risk CKM status. (TIF)

S13 Fig Weighted Kaplan–Meier curves for all‑cause mortality stratified by CKMAI threshold (61.587). (TIF)

S14 Fig Weighted Kaplan–Meier curves for cardiovascular mortality stratified by CKMAI threshold (60.732). (TIF)

S15 Fig Weighted risk plot for high‑risk CKM status stratified by CKMAI threshold (34.092). (TIF)

S16 Fig Forest plot of stratified hazard ratios for all‑cause mortality by CKM stage for CKMAI, PhenoAge, KDM, and CMI. (TIF)

S17 Fig Forest plot of stratified hazard ratios for cardiovascular mortality by CKM stage for CKMAI, PhenoAge, KDM, and CMI. (TIF)

S18 Fig Joint forest plot for additive interaction of CKMAI with PhenoAge, KDM, and CMI on all‑cause mortality. (TIF)

S19 Fig Joint forest plot for additive interaction of CKMAI with PhenoAge, KDM, and CMI on cardiovascular mortality. (TIF)

S20 Fig Joint forest plot for additive interaction of CKMAI with PhenoAge, KDM, and CMI on high‑risk CKM status. (TIF)

S21 Fig Kaplan–Meier survival curves for all‑cause mortality across six aging‑metabolic phenotypes (clusters). (TIF)

S22 Fig Kaplan–Meier survival curves for cardiovascular mortality across six aging‑metabolic phenotypes (clusters). (TIF)

S23 Fig Proportion of participants with high‑risk CKM status (stage ≥3) by cluster, with 95% confidence intervals. (TIF)

S24 Fig Performance comparison of CKMAI, PHENOAGE, KDM, and CMI in the temporal validation set (training: 2005–2010; testing: 2011–2018). (TIF)

S25 Fig Fixed‑weight temporal validation of CKMAI across different standardization schemes. (TIF)

S26 Fig ROC curves for predicting CKM high‑risk status (CKM stage ≥3) in the hospital validation cohort. (TIF)

S27 Fig Calibration plots for CKMAI predictions across demographic subgroups. (TIF)

S1 Table Baseline characteristics of the training set (*n* = 4,827). (DOCX)

S2 Table Baseline characteristics of the testing set (*n* = 2,069). (DOCX)

S3 Table Comparison of CKMAI distribution between the training and testing sets. (DOCX)

S4 Table Complete list of stable features and their bootstrap selection frequencies for all‑cause mortality and CKM high‑risk status. (DOCX)

S5 Table DeLong test *P*‑values for comparisons of time‑dependent AUCs in the testing set. (DOCX)

S6 Table Predictive performance of CKMAI, PhenoAge, KDM, and CMI in the training set. (DOCX)

S7 Table Summary of sensitivity analyses (unweighted, 5‑year follow‑up, and exclusion of baseline CVD/CKD). (DOCX)

S8 Table Mediation analysis of depression score on the associations between CKMAI and outcomes (all‑cause mortality, cardiovascular mortality, and high‑risk CKM status). (DOCX)

S9 Table Baseline characteristics of the study population overall and by survey periods (NHANES 2005–2010 and 2011–2018). (DOCX)

S10 Table C index for all cause and cardiovascular mortality, and AUC for CKM high risk status in the test dataset. (DOCX)

S11 Table Temporal validation of CKMAI and other aging indices across different NHANES cycles (fixed weight analysis). (DOCX)

S12 Table AUC for predicting CKM high-risk status (CKM ≥3) in the hospital validation cohort. (DOCX)

S13 Table Baseline characteristics of the hospital validation cohort stratified by CKM stage. (DOCX)

S14 Table Normality assessment of continuous variables in the hospital validation cohor. (DOCX)

S15 Table Association of CKMAI_advance with clinical outcomes in weighted regression analyses. (DOCX)

S16 Table Comparison of predictive performance: full 18-variable models versus CKMAI. (DOCX)

S17 Table Reverse mediation analysis: depression score → CKMAI → outcome. (DOCX)

S18 Table Moderation analysis: interaction between CKMAI and depression. (DOCX)

S19 Table Discriminative ability of CKMAI for CKM high‑risk status (stage ≥ 3). (DOCX)

S20 Table Variance inflation factors for the candidate indices. (DOCX)

S21 Table Comparison of baseline characteristics between included and excluded participants (aged ≥18 years). (DOCX)

S22 Table Performance of CKMAI, KDM, CMI, and PhenoAge for predicting all‑cause mortality, CVD mortality, and CKM high-risk status across 20 imputed datasets. (DOCX)

S23 Table Summary of performance across 20 imputed datasets. (DOCX)

S24 Table Sensitivity analysis for cardiovascular mortality: comparison of Cox and Fine Gray models. (DOCX)

S25 Table Subgroup performance of the CKMAI across sex and ethnicity strata. (DOCX)

## Abbreviations

ACME: average causal mediation effect
ADE: average direct effect
AI: artificial intelligence
ALP: alkaline phosphatase
AP: attributable proportion
AUC: area under the curve
BIC: Bayesian information criterion
BMI: body mass index
BUN: blood urea nitrogen
CIs: confidence intervals
CKD: chronic kidney disease
CKM: cardiovascular‑kidney‑metabolic
CMI: cardiometabolic index
CVD: cardiovascular disease
DCA: decision curve analysis
eGFR: estimated glomerular filtration rate
FBG: fasting glucose
FDR: false discovery rate
HRs: hazard ratios
IDI: integrated discrimination improvement
MCV: mean corpuscular volume
MICE: multiple imputation by chained equations
NCHS: National Center for Health Statistics
NDI: National Death Index
NHANES: National Health and Nutrition Examination Survey
NRI: net reclassification improvement
ORs: odds ratios
PIR: poverty‑to‑income ratio
PLT: platelet count
RDW: red cell distribution width
RERI: relative excess risk due to interaction
SCR: serum creatinine
SDOH: Social determinants of health
SES: socioeconomic status
SMD: standardized mean differences
TC: total cholesterol
UA: uric acid
UACR: urine albumin‑to‑creatinine ratio
VC: vitamin C
VE: vitamin E
VIF: variance inflation factors
WBC: white blood cell count
WC: waist circumference
WHtR: waist‑to‑height ratio.
