# Supplementary material for: A machine learning-derived aging index for risk stratification and mortality prediction in cardiovascular-kidney-metabolic syndrome: A retrospective cohort study
Source: PLoS Med. 2026 Oct 6;23(10):e1005078. doi: 10.1371/journal.pmed.1005078 (PMC13641365; doi:10.1371/journal.pmed.1005078)
Supplement: Supplementary file 2 [file pmed.1005078.s002.docx]

**S2 Checklist: TRIPOD-AI for Prediction Model Development and Validation**

| Item No. | Section/Topic | Item | Recommendation | Where in manuscript (section/paragraph) |
| --- | --- | --- | --- | --- |
| **Title and abstract** |  |  |  |  |
| 1 | Title | 1a | Identify the study as developing and/or validating a multivariable prediction model | Title |
|  |  | 1b | Indicate the target population, the outcome to be predicted, and the intended setting (e.g., primary care) | Title;  Abstract – Background, para 1;  Abstract – Methods and Findings, para 1 |
| 2 | Abstract | 2 | Provide a structured abstract (TRIPOD+AI for Abstracts) | Abstract – Background, para 1;  Abstract – Methods and Findings, paras 1–2;  Abstract – Conclusions, para 1 |
| **Introduction** |  |  |  |  |
| 3 | Background and objectives | 3a | Explain the medical context and rationale for developing or validating the prediction model | Introduction, paras 1–3 |
|  |  | 3b | State the objectives, including the target population, outcome, and intended use | Introduction, para 4 |
| **Methods** |  |  |  |  |
| 4 | Source of data | 4a | Describe the study design or source of data, including key dates | Methods – Study Design and Data Sources, para 1 |
|  |  | 4b | Describe the setting, locations, and relevant dates | Methods – Study Design and Data Sources, para 1 |
| 5 | Participants | 5a | Describe the eligibility criteria | Methods – Study Population, para 1 |
|  |  | 5b | Describe methods of recruitment and sampling | Methods – Study Population, para 1;  Methods – Study Design and Data Sources, para 1 |
| 6 | Outcome | 6a | Clearly define the outcome, how and when assessed, and the blinding | Methods – Outcome Ascertainment, paras 1–2 |
|  |  | 6b | Report any actions to adjudicate the outcome | Methods – Outcome Ascertainment, para 1 |
| 7 | Predictors / Input features | 7a | Clearly define all input features (predictors), how and when measured, and blinding | Methods – Exposure Assessment: Definition of CKMAI, para 1;  Methods – Covariates, para 1 |
|  |  | 7b | Report any pre-processing of input features (e.g., standardization, imputation) | Methods – Exposure Assessment: Definition of CKMAI, para 1 |
| 8 | Sample size | 8 | Explain how the study size was determined (or provide justification for the final sample size) | Methods – Study Design and Data Sources, para 1 (last sentence) |
| 9 | Missing data | 9a | Describe the extent of missing data and the number of participants with missing data | Methods – Study Population, para 1 |
|  |  | 9b | Describe how missing data were handled (e.g., complete-case, multiple imputation) | Methods – Study Population, para 1 |
| 10 | Model development | 10a | Describe the type of model, algorithm, or statistical modeling technique | Methods – Two-Stage Machine Learning Model Selection, para 1 |
|  |  | 10b | Describe how predictors were selected | Methods – Two-Stage Machine Learning Model Selection, para 2 |
|  |  | 10c | Describe how the final model was selected (e.g., Pareto front optimization) | Methods – Exposure Assessment: Definition of CKMAI, para 3 |
|  |  | 10d | Describe how internal validation was performed (e.g., split-sample, cross-validation) | Methods – Study Population, para 2 |
| 11 | Risk groups | 11 | If done, report how risk groups were created and defined | Results – Threshold and Nonlinear Analysis of CKMAI, para 1;  Results – Table 3 |
| 12 | Model performance | 12a | Report all performance measures (discrimination, calibration, reclassification, decision curve) | Results – Predictive Performance of CKMAI Compared with Existing Indices, para 1 |
|  |  | 12b | Report uncertainty estimates (e.g., 95% CIs) for all performance measures | Results – Predictive Performance of CKMAI Compared with Existing Indices, para 1;  Results – Table 2 |
| 13 | Model updating | 13 | If the model was updated, report details of the updating process | Not applicable |
| 14 | Model interpretation | 14 | Describe how the model is interpreted (e.g., SHAP values, coefficients) | Methods – Two-Stage Machine Learning Model Selection, para 4;  Results – CKMAI Composition and Model Performance, para 1;  Results – Figure 3 |
| 15 | Applicability | 15 | Describe the intended scope of use (e.g., intended users, settings) | Discussion, para 3; Conclusion |
| **Results** |  |  |  |  |
| 16 | Participants | 16a | Describe the flow of participants through the study | Study Population and Baseline Characteristics, para 1; Figure 1 |
|  |  | 16b | Report participant characteristics (demographics, clinical features) | Results – Study Population and Baseline Characteristics, para 2; Table 1 |
|  |  | 16c | Report the number of outcome events | Results – Study Population and Baseline Characteristics, para 1 |
| 17 | Model development | 17 | Report the final prediction model (e.g., all weights, intercept, formula) | Methods – Exposure Assessment: Definition of CKMAI, paras 4–5; Figure 3 |
| 18 | Model performance | 18a | Report model performance in the development data | Results – CKMAI Composition and Model Performance, para 1; Supplementary Table 6 |
|  |  | 18b | Report model performance in the validation data (test set) | Results – Predictive Performance of CKMAI Compared with Existing Indices, para 1; Results – Table 2 |
| 19 | Model updating | 19 | If the model was updated, report performance before and after updating | Not applicable |
| 20 | Sensitivity analysis | 20 | Report sensitivity analyses (e.g., unweighted, 5-year, exclusion of baseline disease) | Results – Subgroup and Sensitivity Analyses, para 2; Supplementary Table 7 |
| **Discussion** |  |  |  |  |
| 21 | Limitations | 21 | Discuss limitations of the study | Discussion, para 12 |
| 22 | Interpretation | 22 | Give overall interpretation of the results in the context of existing evidence | Discussion, paras 1–11 |
| 23 | Implications | 23 | Discuss implications for practice, policy, or future research | Discussion, para 14; Conclusion |
| **Other information** |  |  |  |  |
| 24 | Supplementary information | 24 | Report where the model (code, formula) and supplementary materials are available | Data Availability Statement; Supplementary Materials |
| 25 | Funding | 25 | Give sources of funding and the role of the funders | Funding, para 1 |
| 26 | Conflicts of interest | 26 | Declare any competing interests | Conflict of Interest, para 1 |
| 27 | Registration | 27 | Provide study registration details (if any) | Not applicable |
