# Supplementary material for: A machine learning-derived aging index for risk stratification and mortality prediction in cardiovascular-kidney-metabolic syndrome: A retrospective cohort study
Source: PLoS Med. 2026 Oct 6;23(10):e1005078. doi: 10.1371/journal.pmed.1005078 (PMC13641365; doi:10.1371/journal.pmed.1005078)
Supplement: Supplementary file 3 [file pmed.1005078.s003.docx]

**S3 Protocol: Analysis Plan for “A Machine Learning‑Derived CKM‑Specific Aging Index for Risk Stratification and Mortality Prediction in Cardiovascular‑Kidney‑Metabolic Syndrome”**

**Study registration:** Not applicable (secondary analysis of publicly available NHANES data)
**Ethics:** This is a secondary analysis of de‑identified public data; original NHANES protocols were approved by the NCHS Ethics Review Board.

**1. Study objectives**

- **Primary objective:** To develop and validate a CKM‑specific aging index (CKMAI) using a systematic machine learning framework.
- **Secondary objectives:**
  – To evaluate CKMAI’s predictive performance for all‑cause mortality, cardiovascular mortality, and high‑risk CKM status.
  – To compare CKMAI with existing universal biological age indices (PhenoAge, KDM) and a traditional metabolic index (CMI).
  – To explore additive interactions between CKMAI and universal biological age measures.
  – To identify distinct aging‑metabolic phenotypes via unsupervised clustering.
  – To quantify the mediating role of depression in the CKMAI‑mortality association.

**2. Data source**

- **Database:** National Health and Nutrition Examination Survey (NHANES), cycles 2005–2018.
- **Data availability:** Publicly available at <https://wwwn.cdc.gov/nchs/nhanes/>.
- **Mortality linkage:** National Death Index (NDI) follow‑up through December 31, 2019.

**3. Study population**

**3.1 Inclusion criteria**

- Age ≥ 18 years.
- Complete survival follow‑up data.

**3.2 Exclusion criteria (sequential)**

- Age < 18 years.
- Missing data for CKM stage classification.
- Missing data for primary outcome or core exposure variables.
- Missing data for any covariate used in multivariable models.

**3.3 Final sample**

- N = 6,896 participants (representing 142,649,184 noninstitutionalized US adults when weighted).
- Split: training set 70% (n = 4,827), testing set 30% (n = 2,069) using stratified sampling to preserve CKM stage and survey year distributions.

**4. Exposure (CKMAI)**

- **Definition:** CKMAI is a composite score derived from a Pareto‑optimal Cox proportional hazards model.
- **Domains:** metabolic stress, kidney injury, systemic aging, inflammatory‑nutritional status, psychosocial factors.
- **Variables (18 total):** age, RDW, sex, PIR, ethnicity, marital status, MCV, albumin, ALP, education, diabetes, eGFR, smoking, LYM, UACR, SCR, WBC, BUN.
- **Construction:** All continuous variables standardized to mean 0, SD 1; categorical variables converted to dummy indicators. Over 100 candidate models evaluated using 13 feature selection methods and 15 modeling approaches; Pareto front optimization selected the simple Cox proportional hazards model. Normalized coefficients used as weights, then linearly transformed to 0–100 scale.

**5. Outcomes**

| Outcome | Definition | Source |
| --- | --- | --- |
| **All‑cause mortality** | Death from any cause | NDI linkage |
| **Cardiovascular mortality** | ICD‑10 I00–I09, I11, I13, I20–I51, I60–I69 | NDI linkage |
| **High‑risk CKM status** | CKM stage ≥3 (per AHA definition) | Calculated from baseline data |

**6. Covariates**

Demographic: age, sex, race/ethnicity, education, marital status, poverty‑income ratio (PIR).
Lifestyle: smoking, alcohol consumption, physical activity.
Comorbidities: hypertension, diabetes, dyslipidemia, CKD, cardiovascular disease, cancer, depression.
Laboratory: eGFR, UACR, HbA1c, fasting glucose, lipids, WBC, LYM, MCV, RDW, PLT, albumin, ALP, BUN, SCR, UA, vitamins E, B6, C, total energy intake.

**7. Statistical analysis plan (prespecified)**

**7.1 Weighting**

All analyses account for NHANES complex survey design using sampling weights, strata (SDMVSTRA), and clusters (SDMVPSU).

**7.2 Descriptive statistics**

- Continuous: mean (SD) or median (IQR) after normality test (Shapiro–Wilk).
- Categorical: number (%).
- Baseline table (Table 1) presented without group comparison P values.

**7.3 Model development and validation**

- **Training/testing split:** 70/30.
- **Model selection:** Two‑stage framework (13 feature selection methods × 15 modeling approaches). Performance metrics: Harrell’s C‑index for all‑cause mortality and AUC for high‑risk CKM status. Pareto front optimization selects the simple Cox model.
- **Final model:** simple Cox proportional hazards.

**7.4 Performance evaluation (testing set)**

- Discrimination: time‑dependent AUC (3, 5, 10 years), Harrell’s C‑index.
- Reclassification: net reclassification improvement (NRI), integrated discrimination improvement (IDI).
- Calibration: calibration plots, Greenwood‑Nam‑D’Agostino test.
- Clinical utility: decision curve analysis (DCA).
- Comparison with PhenoAge, KDM, CMI.

**7.5 Threshold and nonlinear analysis**

- Restricted cubic splines (knots at 5th, 35th, 65th, 95th percentiles).
- Two‑piecewise linear/logistic regression (segmented package) to identify inflection points and compare with standard linear model via likelihood ratio test.

**7.6 Additive interactions**

- RERI (relative excess risk due to interaction) and AP (attributable proportion) with bootstrap 95% CI, for CKMAI with PhenoAge, KDM, CMI.

**7.7 Clustering analysis**

- Gaussian mixture models based on age, RDW, MCV, albumin, ALP, PhenoAge.
- Optimal number of clusters determined by Bayesian information criterion (BIC) and silhouette coefficient.
- Mortality risks across clusters estimated using weighted Cox regression with cluster 1 (healthiest) as reference.

**7.8 Mediation analysis**

- Weighted bootstrap (1,000 replicates) to estimate average causal mediation effect (ACME), average direct effect (ADE), and proportion mediated.
- Mediator: depression score (continuous).
- Adjusting for sex, ethnicity, education, age.

**7.9 Subgroup analyses**

- Prespecified subgroups: age (<65 vs. ≥65), sex, race/ethnicity, education, PIR, smoking, drinking, physical activity, hypertension, diabetes, CKD, cancer, CVD.
- Interaction P values adjusted using Benjamini‑Hochberg FDR (q < 0.1).
- Exploratory (post‑hoc) subgroups explicitly indicated as such.

**7.10 Sensitivity analyses**

- Unweighted Cox regression.
- Restrict follow‑up to 5 years.
- Exclude participants with baseline CVD or CKD.

**7.11 Missing data**

- Complete‑case analysis after sequential exclusions (no missing data in final cohort).

**7.12 Software and significance level**

- R version 4.4.0 with packages: survey, survival, rms, mediation, segmented, glmnet, stepAIC, CoxBoost, plsRcox, SuperPCA, randomForestSRC, gbm, fastshap.
- Two‑sided tests, α = 0.05.

**8. Model interpretability**

- SHAP (SHapley Additive exPlanations) values calculated for final model using fastshap package.
- Feature importance summarized as mean absolute SHAP value.

**9. Protocol deviations**

No deviations from this prespecified analysis plan occurred. All analyses were performed as described. Any post‑hoc exploratory analyses are clearly indicated in the manuscript (e.g., some subgroup analyses are explicitly labeled as exploratory).

**10. Ethics and data availability**

- **Ethics:** Original NHANES protocols approved by NCHS Research Ethics Review Board; all participants provided written informed consent. This secondary analysis of de‑identified public data was exempt from additional IRB approval.
- **Data availability:** NHANES data publicly available at <https://wwwn.cdc.gov/nchs/nhanes/>. Analysis code available from corresponding author upon reasonable request.

**11. Dissemination**

Results will be submitted for publication in a peer‑reviewed journal. A web‑based risk calculator (<https://zhuzhengyang.shinyapps.io/shiny/>) has been developed to facilitate clinical implementation.
