# Supplementary material for: A machine learning-derived aging index for risk stratification and mortality prediction in cardiovascular-kidney-metabolic syndrome: A retrospective cohort study
Source: PLoS Med. 2026 Oct 6;23(10):e1005078. doi: 10.1371/journal.pmed.1005078 (PMC13641365; doi:10.1371/journal.pmed.1005078)
Supplement: Supplementary file 4 [file pmed.1005078.s004.docx]

**Construction of socioeconomic status (SES) indicator for the hospital validation cohort**

Because the poverty‑to‑income ratio (PIR) used in the original NHANES model could not be computed in our Chinese hospital cohort (detailed household income was not recorded in medical records), we constructed a three‑tier socioeconomic status (SES) variable using the most reliable available proxies. The classification algorithm was as follows: High SES: Urban residence AND employee insurance OR professional occupation (e.g., government staff, teacher, physician, manager, engineer). Medium SES: Urban residence AND resident insurance OR rural residence AND non‑farming occupation (e.g., worker, service staff, small business owner, technician). Low SES: New Rural Cooperative Medical Scheme (NCMS), self‑pay, OR rural residence AND farming/unemployed.

This classification was designed to correspond to the PIR tertiles used in the primary NHANES analysis: High SES ≈ PIR ≥3.5, Medium SES ≈ PIR 1.3–3.5, Low SES ≈ PIR <1.3. The SES weight in the CKMAI is 0.178 (normalized), which is relatively small compared to age, RDW, and other core biomarkers, reducing the potential impact of this approximation on overall risk stratification.

**Educational attainment derivation in the hospital validation cohort**

In the hospital-based validation cohort, educational attainment was not directly recorded in the medical records. As this variable is a component of the CKMAI model and was required for the external validation, we derived educational categories based on a combination of occupation, workplace, and residential address, which were the only available proxies for socioeconomic status in our institutional data system. Without this derivation, the CKMAI model could not be fully implemented in this cohort, as the original model requires all component variables to be present. Importantly, educational attainment carries a relatively low weight in the CKMAI model (normalized weight of 0.143, ranking 10th among the 18 component variables), minimizing the potential impact of estimation-related uncertainty on the overall CKMAI calculation.

The three educational categories were defined as follows. These mappings were based on widely accepted social stratification patterns in the Chinese context, where occupational category and residential location are closely associated with educational attainment:

**1. Less than high school**

- Occupation as farmer or manual laborer; or
- Rural residential address; or
- Workplace categorized as primary industry or small private enterprise

**2. High school or equivalent**

- Occupation as skilled worker or general staff; or
- Suburban residential address; or
- Workplace categorized as secondary industry or state‑owned enterprise

**3. College or above**

- Occupation as professional, technical, or managerial personnel; or
- Urban residential address; or
- Workplace categorized as tertiary industry, research institution, or government agency

When multiple indicators were available, a hierarchical rule was applied: if any indicator suggested a higher category, that category was assigned. When indicators were ambiguous or contradictory, the most frequently indicated category across all three variables was selected. In cases where all three indicators suggested different categories, the category indicated by occupation was prioritized, as occupation was considered the most directly informative proxy for educational attainment among the three variables. This approach was specifically designed to approximate the NHANES educational categories (less than high school, high school or equivalent, and college or above) and to ensure that the CKMAI model could be consistently applied across the primary and validation cohorts, enabling the external validation to proceed.
