# Supplementary material for: A machine learning-derived aging index for risk stratification and mortality prediction in cardiovascular-kidney-metabolic syndrome: A retrospective cohort study
Source: PLoS Med. 2026 Oct 6;23(10):e1005078. doi: 10.1371/journal.pmed.1005078 (PMC13641365; doi:10.1371/journal.pmed.1005078)
Supplement: Supplementary file 5 [file pmed.1005078.s005.docx]

**Hyperparameter specifications for the two‑stage machine learning pipeline.**

This document provides the complete hyperparameter settings for all feature selection strategies and modeling approaches evaluated in the two‑stage machine learning framework. All analyses were implemented in R version 4.4.0.

**1. Feature Selection Strategies**

Thirteen feature selection strategies were applied in the stability selection phase.

| Method | Hyperparameters / Settings | R package / function |
| --- | --- | --- |
| **Univariate screening** | Log‑rank test; variables with P < 0.05 retained | survival::coxph |
| **Lasso** | α = 1; lambda selected via 3‑fold CV (1‑SE rule); maxit = 100,000; thresh = 1e‑7 | glmnet::cv.glmnet |
| **Ridge** | α = 0; lambda selected via 3‑fold CV (1‑SE rule); maxit = 100,000; thresh = 1e‑7 | glmnet::cv.glmnet |
| **Elastic Net (α = 0.4)** | α = 0.4; lambda selected via 3‑fold CV (1‑SE rule); maxit = 100,000; thresh = 1e‑7 | glmnet::cv.glmnet |
| **Elastic Net (α = 0.6)** | α = 0.6; lambda selected via 3‑fold CV (1‑SE rule); maxit = 100,000; thresh = 1e‑7 | glmnet::cv.glmnet |
| **Elastic Net (α = 0.8)** | α = 0.8; lambda selected via 3‑fold CV (1‑SE rule); maxit = 100,000; thresh = 1e‑7 | glmnet::cv.glmnet |
| **Stepwise Cox (both)** | Direction = "both"; selection based on AIC | stats::step |
| **Stepwise Cox (backward)** | Direction = "backward"; selection based on AIC | stats::step |
| **Stepwise Cox (forward)** | Direction = "forward"; selection based on AIC | stats::step |
| **CoxBoost** | maxstepno = 50; penalty optimized via optimCoxBoostPenalty; K = 3 for CV | CoxBoost::CoxBoost |
| **Supervised PC** | n.threshold = 5; n.fold = 3; n.components = 2; min.features = 5; s0.perc = 0.5 | superpc |
| **Partial least squares Cox** | nt = 3 (number of components); sparse = TRUE | plsRcox::plsRcox |
| **Random Survival Forest** | ntree = 300; nodesize = 5; splitrule = "logrank" | randomForestSRC::rfsrc |
| **Gradient Boosting Machine** | n.trees = 2000; interaction.depth = 3; n.minobsinnode = 10; shrinkage = 0.01; cv.folds = 3 | gbm::gbm |

**2. Modeling Approaches**

Fifteen modeling approaches were evaluated in the second stage.

| Method | Hyperparameters / Settings | R package / function |
| --- | --- | --- |
| **Cox PH** | Default; no tuning | survival::coxph |
| **Lasso** | α = 1; lambda selected via 3‑fold CV (1‑SE rule) | glmnet::cv.glmnet |
| **Ridge** | α = 0; lambda selected via 3‑fold CV (1‑SE rule) | glmnet::cv.glmnet |
| **Elastic Net (α = 0.4)** | α = 0.4; lambda selected via 3‑fold CV (1‑SE rule) | glmnet::cv.glmnet |
| **Elastic Net (α = 0.6)** | α = 0.6; lambda selected via 3‑fold CV (1‑SE rule) | glmnet::cv.glmnet |
| **Elastic Net (α = 0.8)** | α = 0.8; lambda selected via 3‑fold CV (1‑SE rule) | glmnet::cv.glmnet |
| **Stepwise Cox (both)** | Direction = "both"; selection based on AIC | stats::step |
| **Stepwise Cox (backward)** | Direction = "backward"; selection based on AIC | stats::step |
| **Stepwise Cox (forward)** | Direction = "forward"; selection based on AIC | stats::step |
| **CoxBoost** | maxstepno = 50; penalty optimized via optimCoxBoostPenalty; step selected by 3‑fold CV | CoxBoost::CoxBoost |
| **Supervised PC** | threshold selected via 3‑fold CV; n.components = 1 | superpc |
| **Partial least squares Cox** | nt selected via 3‑fold CV; sparse = TRUE | plsRcox::plsRcox |
| **Survival SVM** | gamma.mu = 1; opt.meth = "ipop" | survivalsvm::survivalsvm |
| **Random Survival Forest** | ntree = 300; nodesize = 5; splitrule = "logrank" | randomForestSRC::rfsrc |
| **Gradient Boosting Machine** | n.trees selected by CV; interaction.depth = 3; n.minobsinnode = 10; shrinkage = 0.01 | gbm::gbm |

**3. Stability Selection Parameters**

a) **Bootstrap iterations**: 50
b) **Selection threshold**: 0.6 (variables selected in ≥60% of bootstrap samples were retained)
c) **Resampling**: Bootstrap with replacement, stratified by outcome status
d) **Combination rule**: Variables were selected if they exceeded the threshold in either the all‑cause mortality or CKM high‑risk stability selection

**4. Model Selection and Evaluation**

a) **Performance metrics**: Harrell‘s C‑index for all‑cause mortality; AUC for high‑risk CKM status
b) **Model selection**: Pareto front optimization; the model with the highest sum of standardized ranks for the two metrics was selected
c) **Internal validation**: 70/30 random split of the full NHANES 2005–2018 sample
d) **Temporal validation**: Training on 2005–2010, testing on 2011–2018

**5. Code Availability**

The complete analysis code, including all hyperparameter settings, is available in the GitHub repository: <https://github.com/zhuzhengyang517/CKMAI_paper_code.git>
