# Supplementary material for: A machine learning-derived aging index for risk stratification and mortality prediction in cardiovascular-kidney-metabolic syndrome: A retrospective cohort study
Source: PLoS Med. 2026 Oct 6;23(10):e1005078. doi: 10.1371/journal.pmed.1005078 (PMC13641365; doi:10.1371/journal.pmed.1005078)
Supplement: Supplementary file 6 [file pmed.1005078.s006.docx]

**CKMAIcalc R package: user guide.**

**1. Package overview**

CKMAIcalc is an R package for calculating the **Composite Kidney‑Metabolic‑Aging Index (CKMAI)** and its associated risk predictions. It is based on 18 clinical and laboratory variables, automatically handles dummy variable encoding, and includes pre‑trained Cox proportional hazards and logistic regression models. The package outputs:

a) CKMAI score (0–100 scale)
b) CKMAI_advance (CKMAI minus chronological age)
c) 10‑year all‑cause mortality risk (%)
d) 10‑year cardiovascular mortality risk (%)
e) CKM high‑risk (stage ≥3) probability (%)

All model parameters are embedded within the package, ensuring consistency with the derivation cohort and eliminating the need for external parameter files.

**2. Installation**

From GitHub (recommended):

r

install.packages("devtools")

devtools::install_github("zhuzhengyang517/CKMAIcalc")

**3. Loading the package**

r

library(CKMAIcalc)

**4. Core functions**

**4.1** compute_CKMAI(data)

Computes the CKMAI score (0–100 scale). Higher scores indicate higher risk.

1. **Input**: data – a data frame containing the required variables (all numeric). Categorical variables can be provided in original coding; the function automatically generates dummy variables.
   a) Continuous variables: Age, WBC, LYM, MCV, RDW, ALB, ALP, BUN, SCR, UACR, EGFR
   b) Categorical variables (original coding): Sex (1=male, 2=female), Smoking (0/1), Diabetes (0/1), Education (1/2/3), Ethnicity (1/2/3/4), Marital_Status (1/2/3), PIR (1/2/3)
2. **Output**: Numeric vector of CKMAI scores.

**Example:**

r

new_data <- data.frame(

Age = 65, Sex = 1, Smoking = 1, Diabetes = 0,

Education = 3, Ethnicity = 1, Marital_Status = 0, PIR = 2,

WBC = 7.5, LYM = 30, MCV = 90, RDW = 13.5,

ALB = 4.2, ALP = 70, BUN = 15, SCR = 0.9, UACR = 10, EGFR = 90

)

ckmai <- compute_CKMAI(new_data)

print(ckmai) *# e.g., 42.3*

**4.2** compute_CKMAI_advance(data)

Computes CKMAI − Age, representing the age‑adjusted component of the index.

- **Input**: Same as compute_CKMAI; must include Age.
- **Output**: Numeric vector of CKMAI_advance values.

**Example:**

r

ckmai_adv <- compute_CKMAI_advance(new_data)

print(ckmai_adv) *# e.g., 42.3 - 65 = -22.7*

**4.3** predict_risks(data, times = 120, as_percent = TRUE)

Predicts 10‑year (default) risks for all‑cause mortality, cardiovascular mortality, and CKM high‑risk status.

- **Input**:
  a) data: Same as compute_CKMAI, with additional required variables: Hypertension (0/1), EGFR, UACR.
  b) times: Prediction time point in months (default 120 for 10 years; e.g., 60 for 5 years).
  c) as_percent: Logical; if TRUE (default), returns percentages (0–100); if FALSE, returns probabilities (0–1).
- **Output**: Original data frame with three new columns:
  a) risk_all_pct / risk_all: All‑cause mortality risk
  b) risk_cvd_pct / risk_cvd: Cardiovascular mortality risk
  c) risk_ckm_high_pct / risk_ckm_high: CKM high‑risk probability

**Example:**

r

new_data <- predict_risks(new_data, times = 120, as_percent = TRUE)

print(new_data[, c("CKMAI", "risk_all_pct", "risk_cvd_pct", "risk_ckm_high_pct")])

**5. Data requirements**

a) All input variables must be numeric. Missing values (NA) will cause the corresponding row to be skipped; missing data should be handled prior to analysis.
b) Categorical variables can be provided in original coding; the package automatically converts them to the required dummy variables. If dummy variables (e.g., Sex1) already exist in the data, they will be used directly.
c) predict_risks requires Hypertension, EGFR, and UACR in addition to the CKMAI variables.

**6. Complete workflow example**

r

library(CKMAIcalc)

df <- read.csv("my_data.csv")

*# Compute CKMAI and CKMAI_advance*

df$CKMAI <- compute_CKMAI(df)

df$CKMAI_advance <- compute_CKMAI_advance(df)

*# Predict 10‑year risks (percentages)*

df <- predict_risks(df, times = 120)

*# View results*

head(df[, c("ID", "CKMAI", "CKMAI_advance", "risk_all_pct", "risk_cvd_pct", "risk_ckm_high_pct")])

*# Save results*

write.csv(df, "my_data_with_risks.csv", row.names = FALSE)

**7. Model parameters**

All standardization parameters (means and SDs) and model coefficients were derived from the NHANES 2005‑2018 70% training set, following stability selection and Pareto front optimization. All models are embedded within the package, ensuring reproducibility and consistency with the original study.

**8. Citation**

If you use this package in your research, please cite:

Zhu Z, et al. CKMAIcalc: An R package for calculating the Composite Kidney‑Metabolic‑Aging Index. GitHub repository, <https://github.com/zhuzhengyang517/CKMAIcalc>.
