# Supplementary material for: A machine learning-derived aging index for risk stratification and mortality prediction in cardiovascular-kidney-metabolic syndrome: A retrospective cohort study
Source: PLoS Med. 2026 Oct 6;23(10):e1005078. doi: 10.1371/journal.pmed.1005078 (PMC13641365; doi:10.1371/journal.pmed.1005078)
Supplement: Supplementary file 7 [file pmed.1005078.s035.docx]

**Supplementary Table 1**. Demographic and clinical characteristics of the training cohort

| Variable | Total  (n = 4827) | Stage 0  (n=360) | Stage 1  (n=774) | Stage 2  (n=2866) | Stage 3  (n=267) | Stage 4  (n=560) |
| --- | --- | --- | --- | --- | --- | --- |
|  |  |  |  |  |  |  |
| Age, median (IQR), years | 47.00 (34.00, 61.00) | 29.00 (23.00, 40.00) | 40.00 (29.00, 52.00) | 50.00 (36.00, 61.00) | 52.00 (45.00, 62.00) | 65.00 (56.00, 73.00) |
| BMI, median (IQR), kg/m² | 28.10 (24.22, 32.90) | 21.10 (19.57, 22.61) | 27.08 (24.30, 31.00) | 29.20 (25.47, 33.97) | 31.20 (28.60, 34.01) | 29.22 (25.20, 33.20) |
| WC, median (IQR), cm | 98.80 (88.00, 110.50) | 76.80 (72.80, 81.40) | 94.70 (88.00, 104.10) | 101.70 (92.10, 113.00) | 108.40 (101.30, 115.50) | 104.80 (94.50, 115.60) |
| WHtR, median (IQR) | 0.58 (0.52, 0.65) | 0.46 (0.44, 0.48) | 0.56 (0.51, 0.61) | 0.60 (0.54, 0.67) | 0.62 (0.58, 0.67) | 0.61 (0.56, 0.69) |
| Depression score, median (IQR) | 2.00 (0.00, 4.00) | 2.00 (0.00, 3.00) | 2.00 (0.00, 4.00) | 2.00 (0.00, 4.00) | 1.00 (0.00, 3.00) | 2.00 (0.00, 6.00) |
| WBC, median (IQR), ×1000 cells/μL | 6.50 (5.50, 7.90) | 5.80 (4.80, 7.10) | 6.30 (5.40, 7.60) | 6.70 (5.60, 8.00) | 6.90 (5.70, 8.40) | 6.60 (5.60, 8.10) |
| LYM, median (IQR), % | 30.10 (24.80, 35.50) | 32.40 (26.90, 37.60) | 31.10 (26.80, 35.80) | 29.80 (24.50, 35.30) | 28.60 (22.70, 32.80) | 27.10 (21.40, 33.60) |
| MCV, median (IQR), fL | 89.80 (86.60, 92.50) | 90.60 (87.20, 92.70) | 89.60 (86.70, 92.30) | 89.50 (86.40, 92.30) | 89.90 (86.60, 93.00) | 90.50 (87.30, 93.50) |
| RDW, median (IQR), % | 13.00 (12.40, 13.60) | 12.80 (12.30, 13.20) | 12.90 (12.50, 13.50) | 13.00 (12.40, 13.60) | 13.00 (12.50, 13.50) | 13.40 (12.70, 14.00) |
| HbA1c, median (IQR), % | 5.50 (5.20, 5.80) | 5.10 (5.00, 5.30) | 5.40 (5.10, 5.60) | 5.50 (5.20, 5.80) | 5.70 (5.50, 6.20) | 5.70 (5.40, 6.20) |
| ALB, median (IQR), g/dL | 4.20 (4.00, 4.50) | 4.40 (4.10, 4.60) | 4.30 (4.00, 4.50) | 4.20 (4.00, 4.40) | 4.30 (4.10, 4.50) | 4.10 (3.90, 4.40) |
| ALP, median (IQR), U/L | 66.00 (54.00, 80.00) | 56.00 (47.00, 67.00) | 62.00 (53.00, 77.00) | 68.00 (56.00, 81.00) | 66.00 (55.00, 82.00) | 73.00 (58.00, 90.00) |
| BUN, median (IQR), mg/dL | 13.00 (11.00, 16.00) | 12.00 (9.00, 15.00) | 13.00 (11.00, 16.00) | 13.00 (11.00, 16.00) | 15.00 (11.00, 19.00) | 15.00 (12.00, 19.00) |
| UACR, median (IQR), mg/g | 6.33 (4.23, 11.69) | 6.27 (4.21, 9.14) | 5.28 (3.83, 7.61) | 6.51 (4.31, 12.87) | 7.30 (4.62, 19.15) | 9.56 (5.26, 25.35) |
| EGFR, median (IQR), mL/min/1.73m² | 98.78 (83.92, 111.81) | 111.15 (99.24, 123.23) | 103.40 (90.66, 116.14) | 98.15 (83.19, 110.39) | 92.47 (79.07, 105.10) | 83.08 (66.41, 96.02) |
| TG, median (IQR), mg/dL | 105.00 (71.00, 156.00) | 62.00 (48.00, 78.00) | 75.00 (55.00, 97.00) | 133.00 (88.00, 181.00) | 138.00 (97.00, 207.00) | 116.00 (79.00, 169.00) |
| HDL, median (IQR), mg/dL | 51.00 (42.00, 63.00) | 64.00 (53.00, 75.00) | 56.00 (48.00, 66.00) | 48.00 (40.00, 60.00) | 45.00 (37.00, 52.00) | 48.00 (40.00, 59.00) |
| TG/HDL, median (IQR) | 2.00 (1.18, 3.57) | 1.02 (0.70, 1.33) | 1.33 (0.90, 1.87) | 2.61 (1.59, 4.22) | 3.13 (1.91, 5.03) | 2.45 (1.41, 4.18) |
| CMI, median (IQR) | 1.19 (0.64, 2.21) | 0.46 (0.32, 0.62) | 0.75 (0.49, 1.09) | 1.60 (0.93, 2.63) | 2.03 (1.21, 3.08) | 1.59 (0.86, 2.66) |
| PHENOAGE, median (IQR) | 58.19 (44.00, 71.31) | 37.96 (28.15, 47.94) | 49.22 (38.39, 61.05) | 60.31 (48.19, 72.35) | 64.75 (55.99, 78.46) | 75.09 (64.74, 83.28) |
| KDM, median (IQR) | 40.75 (29.47, 53.50) | 23.50 (17.89, 31.83) | 32.78 (25.24, 42.18) | 44.36 (33.47, 56.24) | 48.76 (39.25, 63.78) | 53.85 (41.07, 69.55) |
| CKMAI, median (IQR) | 40.25 (27.87, 51.47) | 25.37 (18.58, 33.18) | 33.58 (23.44, 45.45) | 42.26 (29.87, 51.52) | 47.95 (39.12, 58.88) | 56.02 (46.74, 63.55) |
| Weight, median (IQR), kg | 81.60 (68.20, 96.60) | 59.00 (53.60, 66.80) | 79.90 (68.00, 91.50) | 84.60 (72.20, 99.30) | 96.60 (85.00, 105.40) | 83.30 (70.30, 98.00) |
| Height, median (IQR), cm | 169.50 (162.30, 176.90) | 167.20 (161.90, 174.00) | 169.30 (163.10, 176.90) | 169.50 (161.50, 177.00) | 174.20 (168.30, 179.30) | 169.10 (162.70, 175.60) |
| VE, median (IQR), mg | 0.06 (0.00, 0.35) | 0.07 (0.00, 0.54) | 0.06 (0.00, 0.40) | 0.06 (0.00, 0.33) | 0.09 (0.00, 0.33) | 0.05 (0.00, 0.25) |
| B6, median (IQR), mg | 0.01 (0.00, 0.15) | 0.02 (0.00, 0.18) | 0.01 (0.00, 0.16) | 0.01 (0.00, 0.15) | 0.01 (0.00, 0.13) | 0.01 (0.00, 0.11) |
| VC, median (IQR), mg | 0.00 (0.00, 0.10) | 0.00 (0.00, 0.10) | 0.00 (0.00, 0.00) | 0.00 (0.00, 0.10) | 0.00 (0.00, 0.30) | 0.00 (0.00, 0.10) |
| TC, median (IQR), mg/dL | 190.00 (164.00, 219.00) | 172.00 (151.00, 191.00) | 183.00 (160.00, 209.00) | 198.00 (171.00, 226.00) | 187.00 (161.00, 228.00) | 174.00 (147.00, 203.00) |
| PLT, median (IQR), ×1000 cells/μL | 239.00 (204.00, 283.00) | 234.00 (201.00, 274.00) | 234.00 (203.00, 276.00) | 248.00 (209.00, 289.00) | 233.00 (193.00, 273.00) | 217.00 (184.00, 264.00) |
| FBG, median (IQR), mg/dL | 102.00 (95.00, 110.00) | 91.00 (87.00, 95.00) | 102.00 (97.00, 107.00) | 102.00 (95.00, 112.00) | 111.00 (103.00, 128.00) | 107.00 (99.00, 122.00) |
| SCR, median (IQR), mg/dL | 0.86 (0.73, 1.00) | 0.80 (0.68, 0.90) | 0.84 (0.72, 0.96) | 0.85 (0.73, 1.00) | 0.95 (0.84, 1.11) | 0.93 (0.81, 1.10) |
| UA, median (IQR), mg/dL | 5.50 (4.60, 6.40) | 4.50 (3.90, 5.20) | 5.20 (4.50, 6.10) | 5.70 (4.80, 6.60) | 6.10 (5.30, 7.10) | 5.80 (4.90, 6.80) |
| Energy Intake, median (IQR), kcal | 2005.50 (1538.00, 2584.00) | 2003.50 (1545.50, 2558.50) | 2099.50 (1626.00, 2600.50) | 1996.50 (1535.00, 2600.50) | 2134.00 (1605.50, 2765.00) | 1764.50 (1391.50, 2244.00) |
| Follow up, median (IQR), months | 66.00 (36.00, 137.00) | 58.00 (37.00, 141.00) | 51.00 (33.00, 133.00) | 110.00 (36.00, 139.00) | 121.00 (51.00, 143.00) | 62.00 (32.00, 126.00) |
| Sex, % |  |  |  |  |  |  |
| Male | 2559 (52.44) | 124 (32.76) | 413 (53.26) | 1464 (51.32) | 215 (83.23) | 343 (60.55) |
| Female | 2268 (47.56) | 236 (67.24) | 361 (46.74) | 1402 (48.68) | 52 (16.77) | 217 (39.45) |
| Ethnicity, % |  |  |  |  |  |  |
| Mexican American | 771 (7.64) | 34 (4.65) | 152 (9.89) | 503 (8.19) | 33 (4.88) | 49 (3.75) |
| Non-Hispanic White | 2279 (70.95) | 167 (69.74) | 347 (70.14) | 1307 (70.63) | 139 (74.61) | 319 (73.96) |
| Non-Hispanic Black | 888 (9.59) | 89 (13.69) | 109 (7.02) | 521 (9.51) | 50 (9.43) | 119 (11.64) |
| Other | 889 (11.82) | 70 (11.91) | 166 (12.95) | 535 (11.67) | 45 (11.09) | 73 (10.66) |
| Education, % |  |  |  |  |  |  |
| Less than high school | 1090 (14.39) | 46 (9.33) | 155 (12.11) | 659 (14.08) | 69 (20.29) | 161 (23.48) |
| High school or equivalent | 1171 (24.35) | 78 (19.32) | 163 (21.34) | 701 (25.11) | 72 (26.57) | 157 (30.20) |
| College or above | 2566 (61.25) | 236 (71.35) | 456 (66.55) | 1506 (60.80) | 126 (53.14) | 242 (46.31) |
| Marital Status, % |  |  |  |  |  |  |
| Married or living with a partner | 3017 (65.98) | 172 (55.65) | 505 (68.66) | 1801 (65.32) | 205 (79.30) | 334 (67.33) |
| Single (widowed/divorced/separated) | 1044 (17.69) | 30 (5.83) | 119 (13.89) | 663 (19.78) | 41 (12.85) | 191 (28.14) |
| Never married | 766 (16.33) | 158 (38.52) | 150 (17.46) | 402 (14.90) | 21 (7.86) | 35 (4.53) |
| PIR, % |  |  |  |  |  |  |
| <1.3 | 1368 (18.47) | 98 (17.85) | 199 (16.53) | 809 (18.46) | 74 (17.66) | 188 (24.00) |
| 1.3 to <3.5 | 1901 (36.71) | 132 (33.09) | 305 (36.39) | 1129 (36.78) | 92 (33.20) | 243 (42.99) |
| >=3.5 | 1558 (44.82) | 130 (49.05) | 270 (47.08) | 928 (44.76) | 101 (49.13) | 129 (33.00) |
| Smoking, % |  |  |  |  |  |  |
| No | 2517 (52.02) | 222 (61.24) | 447 (56.90) | 1510 (52.16) | 120 (42.22) | 218 (36.08) |
| Yes | 2310 (47.98) | 138 (38.76) | 327 (43.10) | 1356 (47.84) | 147 (57.78) | 342 (63.92) |
| Drinking, % |  |  |  |  |  |  |
| No | 1544 (27.85) | 98 (24.98) | 213 (23.37) | 953 (29.18) | 65 (22.56) | 215 (35.42) |
| Yes | 3283 (72.15) | 262 (75.02) | 561 (76.63) | 1913 (70.82) | 202 (77.44) | 345 (64.58) |
| Physical Activity, % |  |  |  |  |  |  |
| Low physical activity | 1818 (32.30) | 98 (23.27) | 216 (24.85) | 1124 (34.65) | 96 (28.20) | 284 (45.78) |
| High physical activity | 3009 (67.70) | 262 (76.73) | 558 (75.15) | 1742 (65.35) | 171 (71.80) | 276 (54.22) |
| Hypertension, % |  |  |  |  |  |  |
| No | 2614 (59.90) | 360 (100.00) | 774 (100.00) | 1351 (50.26) | 3 (0.67) | 126 (25.41) |
| Yes | 2213 (40.10) | 0 (0.00) | 0 (0.00) | 1515 (49.74) | 264 (99.33) | 434 (74.59) |
| Diabetes, % |  |  |  |  |  |  |
| No | 3738 (83.37) | 358 (99.79) | 755 (98.05) | 2162 (81.13) | 147 (58.78) | 316 (62.11) |
| Yes | 1089 (16.63) | 2 (0.21) | 19 (1.95) | 704 (18.87) | 120 (41.22) | 244 (37.89) |
| CKD, % |  |  |  |  |  |  |
| No | 4026 (87.43) | 360 (100.00) | 774 (100.00) | 2367 (85.14) | 177 (74.72) | 348 (68.31) |
| Yes | 801 (12.57) | 0 (0.00) | 0 (0.00) | 499 (14.86) | 90 (25.28) | 212 (31.69) |
| Cancer, % |  |  |  |  |  |  |
| No | 4358 (90.72) | 347 (94.42) | 730 (93.77) | 2619 (91.20) | 213 (83.88) | 449 (80.82) |
| Yes | 469 (9.28) | 13 (5.58) | 44 (6.23) | 247 (8.80) | 54 (16.12) | 111 (19.18) |
| Depression, % |  |  |  |  |  |  |
| No | 4433 (92.99) | 338 (95.13) | 726 (94.00) | 2640 (93.15) | 249 (94.02) | 480 (86.71) |
| Yes | 394 (7.01) | 22 (4.87) | 48 (6.00) | 226 (6.85) | 18 (5.98) | 80 (13.29) |
| Mortality Status, % |  |  |  |  |  |  |
| No | 4367 (93.40) | 355 (98.53) | 753 (98.24) | 2639 (94.26) | 226 (89.01) | 394 (73.86) |
| Yes | 460 (6.60) | 5 (1.47) | 21 (1.76) | 227 (5.74) | 41 (10.99) | 166 (26.14) |
| Death CVD, % |  |  |  |  |  |  |
| No | 4688 (97.87) | 360 (100.00) | 767 (99.38) | 2800 (98.09) | 259 (98.13) | 502 (90.61) |
| Yes | 139 (2.13) | 0 (0.00) | 7 (0.62) | 66 (1.91) | 8 (1.87) | 58 (9.39) |
| Death Renal, % |  |  |  |  |  |  |
| No | 4821 (99.93) | 360 (100.00) | 774 (100.00) | 2864 (99.97) | 266 (99.77) | 557 (99.55) |
| Yes | 6 (0.07) | 0 (0.00) | 0 (0.00) | 2 (0.03) | 1 (0.23) | 3 (0.45) |
| Abbreviations: BMI, body mass index; WC, waist circumference; WHtR, waist‑to‑height ratio; WBC, white blood cell count; LYM, lymphocyte percentage; MCV, mean corpuscular volume; RDW, red cell distribution width; PLT, platelet count; HbA1c, glycated hemoglobin; FBG, fasting blood glucose; ALP, alkaline phosphatase; BUN, blood urea nitrogen; SCR, serum creatinine; UA, uric acid; UACR, urine albumin‑to‑creatinine ratio; eGFR, estimated glomerular filtration rate; TC, total cholesterol; HDL, high‑density lipoprotein; TG, triglycerides; CMI, cardiometabolic index; VE, vitamin E; B6, vitamin B6; VC, vitamin C; PHENOAGE, phenotypic age; KDM, KDM biological age; CKMAI, CKM‑specific aging index; PIR, poverty‑to‑income ratio; CKD, chronic kidney disease; CVD, cardiovascular disease. Percentages are weighted to account for complex survey design. | | | | | | |
