# Supplementary material for: A machine learning-derived aging index for risk stratification and mortality prediction in cardiovascular-kidney-metabolic syndrome: A retrospective cohort study
Source: PLoS Med. 2026 Oct 6;23(10):e1005078. doi: 10.1371/journal.pmed.1005078 (PMC13641365; doi:10.1371/journal.pmed.1005078)
Supplement: Supplementary file 8 [file pmed.1005078.s036.docx]

**Supplementary Table 2.** Demographic and clinical characteristics of the testing cohort

| Variable | Total  (n = 2069) | Stage 0  (n=146) | Stage 1  (n=344) | Stage 2  (n=1239) | Stage 3  (n=107) | Stage 4  (n=233) |
| --- | --- | --- | --- | --- | --- | --- |
|  |  |  |  |  |  |  |
| Age, median (IQR), years | 48.00 (34.00, 60.00) | 33.00 (25.00, 47.00) | 40.00 (30.00, 50.00) | 50.00 (36.00, 61.00) | 50.00 (45.00, 62.00) | 66.00 (56.00, 76.00) |
| BMI, median (IQR), kg/m² | 28.21 (24.30, 32.97) | 21.60 (19.85, 22.77) | 26.70 (23.80, 30.70) | 29.40 (26.10, 34.25) | 29.68 (27.31, 33.30) | 30.80 (26.10, 36.13) |
| WC, median (IQR), cm | 98.80 (87.20, 110.00) | 77.70 (73.40, 83.30) | 93.50 (85.30, 104.70) | 101.00 (91.40, 112.00) | 106.20 (98.30, 114.20) | 106.60 (96.10, 116.00) |
| WHtR, median (IQR) | 0.58 (0.52, 0.65) | 0.47 (0.44, 0.49) | 0.56 (0.51, 0.61) | 0.59 (0.55, 0.66) | 0.60 (0.56, 0.65) | 0.64 (0.57, 0.70) |
| Depression score, median (IQR) | 2.00 (0.00, 4.00) | 1.00 (0.00, 3.00) | 1.00 (0.00, 4.00) | 2.00 (0.00, 4.00) | 1.00 (0.00, 3.00) | 2.00 (0.00, 5.00) |
| WBC, median (IQR), ×1000 cells/μL | 6.50 (5.50, 7.80) | 6.10 (5.00, 6.90) | 6.20 (5.20, 7.30) | 6.60 (5.50, 7.80) | 7.10 (5.70, 8.50) | 7.00 (5.70, 8.20) |
| LYM, median (IQR), % | 30.00 (24.60, 34.80) | 31.90 (26.80, 36.30) | 30.20 (25.30, 34.30) | 30.30 (25.20, 35.30) | 26.60 (23.00, 31.80) | 26.20 (21.20, 31.80) |
| MCV, median (IQR), fL | 89.60 (86.70, 92.60) | 90.60 (87.90, 93.20) | 88.90 (86.40, 92.10) | 89.60 (86.80, 92.50) | 89.40 (84.90, 91.90) | 90.90 (86.80, 93.70) |
| RDW, median (IQR), % | 13.00 (12.40, 13.60) | 12.70 (12.20, 13.30) | 12.90 (12.50, 13.60) | 13.00 (12.40, 13.60) | 13.00 (12.30, 13.50) | 13.30 (13.00, 14.00) |
| HbA1c, median (IQR), % | 5.50 (5.20, 5.80) | 5.20 (5.00, 5.40) | 5.40 (5.10, 5.60) | 5.50 (5.20, 5.80) | 5.40 (5.30, 6.00) | 5.80 (5.60, 6.60) |
| ALB, median (IQR), g/dL | 4.30 (4.00, 4.50) | 4.30 (4.10, 4.50) | 4.30 (4.10, 4.50) | 4.20 (4.00, 4.50) | 4.30 (4.10, 4.50) | 4.10 (3.90, 4.30) |
| ALP, median (IQR), U/L | 65.00 (55.00, 80.00) | 57.00 (47.00, 64.00) | 63.00 (53.00, 77.00) | 68.00 (56.00, 82.00) | 64.00 (57.00, 78.00) | 70.00 (60.00, 88.00) |
| BUN, median (IQR), mg/dL | 13.00 (11.00, 16.00) | 12.00 (10.00, 14.00) | 13.00 (11.00, 16.00) | 13.00 (11.00, 16.00) | 15.00 (13.00, 18.00) | 15.00 (12.00, 20.00) |
| UACR, median (IQR), mg/g | 6.17 (4.29, 10.70) | 5.60 (4.26, 10.25) | 5.01 (4.00, 7.50) | 6.35 (4.43, 11.27) | 6.76 (4.00, 14.82) | 8.51 (5.37, 19.27) |
| EGFR, median (IQR), mL/min/1.73m² | 98.04 (83.47, 110.69) | 107.07 (93.09, 119.60) | 102.74 (90.03, 116.04) | 97.76 (83.49, 109.58) | 90.66 (77.33, 106.88) | 81.98 (66.82, 94.99) |
| TG, median (IQR), mg/dL | 106.00 (72.00, 156.00) | 62.00 (47.00, 82.00) | 78.00 (58.00, 102.00) | 133.00 (85.00, 178.00) | 134.00 (91.00, 198.00) | 113.00 (77.00, 160.00) |
| HDL, median (IQR), mg/dL | 51.00 (42.00, 64.00) | 64.00 (54.00, 73.00) | 55.00 (46.00, 66.00) | 49.00 (41.00, 61.00) | 44.00 (37.00, 53.00) | 48.00 (40.00, 61.00) |
| TG/HDL, median (IQR) | 2.02 (1.19, 3.46) | 1.00 (0.69, 1.39) | 1.43 (0.93, 2.00) | 2.65 (1.46, 4.08) | 3.38 (1.72, 5.18) | 2.40 (1.33, 3.70) |
| CMI, median (IQR) | 1.19 (0.65, 2.16) | 0.48 (0.33, 0.65) | 0.84 (0.49, 1.16) | 1.60 (0.89, 2.50) | 2.16 (0.98, 3.32) | 1.56 (0.83, 2.39) |
| PHENOAGE, median (IQR) | 57.25 (44.31, 70.80) | 40.77 (32.70, 51.58) | 49.50 (38.63, 59.97) | 58.06 (46.82, 70.79) | 63.24 (53.48, 77.30) | 77.74 (68.95, 87.78) |
| KDM, median (IQR) | 41.55 (30.11, 53.65) | 24.91 (20.56, 32.55) | 32.45 (24.50, 41.13) | 44.55 (34.38, 56.10) | 47.66 (36.81, 60.10) | 56.55 (44.92, 71.41) |
| CKMAI, median (IQR) | 39.23 (28.38, 51.99) | 27.26 (18.34, 37.27) | 33.76 (24.39, 42.77) | 41.37 (29.23, 52.04) | 45.24 (37.75, 54.75) | 59.56 (47.26, 64.33) |
| Weight, median (IQR), kg | 81.30 (67.90, 97.10) | 60.60 (53.10, 67.70) | 77.20 (67.50, 90.30) | 84.50 (71.00, 99.20) | 92.40 (83.50, 103.20) | 86.80 (70.10, 103.60) |
| Height, median (IQR), cm | 168.90 (161.80, 176.60) | 168.50 (162.40, 174.30) | 169.10 (161.80, 177.30) | 168.40 (161.60, 176.10) | 176.60 (170.90, 180.90) | 168.10 (159.60, 176.40) |
| VE, median (IQR), mg | 0.07 (0.00, 0.36) | 0.08 (0.00, 0.61) | 0.05 (0.00, 0.28) | 0.07 (0.00, 0.38) | 0.08 (0.02, 0.41) | 0.08 (0.00, 0.25) |
| B6, median (IQR), mg | 0.02 (0.00, 0.16) | 0.03 (0.00, 0.16) | 0.02 (0.00, 0.13) | 0.02 (0.00, 0.19) | 0.04 (0.00, 0.20) | 0.01 (0.00, 0.12) |
| VC, median (IQR), mg | 0.00 (0.00, 0.30) | 0.00 (0.00, 0.20) | 0.00 (0.00, 0.10) | 0.00 (0.00, 0.40) | 0.00 (0.00, 0.80) | 0.00 (0.00, 0.00) |
| TC, median (IQR), mg/dL | 191.00 (167.00, 218.00) | 180.00 (160.00, 198.00) | 190.00 (164.00, 214.00) | 194.00 (172.00, 228.00) | 185.00 (169.00, 204.00) | 172.00 (142.00, 199.00) |
| PLT, median (IQR), ×1000 cells/μL | 243.00 (207.00, 284.00) | 236.00 (203.00, 267.00) | 242.00 (207.00, 279.00) | 251.00 (210.00, 294.00) | 236.00 (199.00, 279.00) | 220.00 (192.00, 256.00) |
| FBG, median (IQR), mg/dL | 101.00 (95.00, 111.00) | 93.00 (89.00, 96.00) | 102.00 (97.00, 107.00) | 102.00 (96.00, 112.00) | 107.00 (99.00, 117.00) | 110.00 (99.00, 131.00) |
| SCR, median (IQR), mg/dL | 0.85 (0.72, 1.00) | 0.80 (0.72, 0.92) | 0.83 (0.71, 0.94) | 0.84 (0.71, 1.00) | 1.02 (0.89, 1.11) | 0.94 (0.80, 1.08) |
| UA, median (IQR), mg/dL | 5.50 (4.60, 6.40) | 4.40 (3.90, 5.40) | 5.10 (4.30, 5.90) | 5.60 (4.70, 6.50) | 6.40 (5.70, 7.20) | 5.80 (4.70, 6.80) |
| Energy Intake, median (IQR), kcal | 2038.00 (1532.50, 2602.00) | 2173.50 (1550.00, 2605.50) | 2061.00 (1590.00, 2769.00) | 2007.50 (1519.00, 2562.00) | 2227.00 (1692.50, 2834.00) | 1827.50 (1446.00, 2465.50) |
| Follow up, median (IQR), months | 132.00 (110.00, 151.00) | 132.00 (110.00, 156.00) | 129.00 (83.00, 149.00) | 134.00 (115.00, 153.00) | 128.00 (114.00, 149.00) | 120.00 (56.00, 138.00) |
| Sex, % |  |  |  |  |  |  |
| Male | 1044 (49.81) | 45 (35.86) | 167 (49.31) | 601 (48.21) | 91 (89.09) | 140 (55.04) |
| Female | 1025 (50.19) | 101 (64.14) | 177 (50.69) | 638 (51.79) | 16 (10.91) | 93 (44.96) |
| Ethnicity, % |  |  |  |  |  |  |
| Mexican American | 354 (8.04) | 13 (3.50) | 72 (11.01) | 228 (8.56) | 17 (5.89) | 24 (4.20) |
| Non-Hispanic White | 940 (69.96) | 69 (72.26) | 140 (66.37) | 548 (69.20) | 56 (74.33) | 127 (77.93) |
| Non-Hispanic Black | 367 (9.43) | 36 (12.63) | 53 (8.63) | 213 (9.02) | 13 (6.88) | 52 (11.94) |
| Other | 408 (12.57) | 28 (11.62) | 79 (14.00) | 250 (13.22) | 21 (12.91) | 30 (5.93) |
| Education, % |  |  |  |  |  |  |
| Less than high school | 475 (13.58) | 16 (6.96) | 63 (11.87) | 292 (14.19) | 31 (11.79) | 73 (20.95) |
| High school or equivalent | 497 (25.19) | 31 (20.41) | 79 (22.21) | 301 (26.46) | 28 (31.53) | 58 (24.32) |
| College or above | 1097 (61.23) | 99 (72.63) | 202 (65.92) | 646 (59.35) | 48 (56.68) | 102 (54.73) |
| Marital Status, % |  |  |  |  |  |  |
| Married or living with a partner | 1251 (63.63) | 78 (58.37) | 204 (61.48) | 768 (64.77) | 78 (76.98) | 123 (58.54) |
| Single (widowed/divorced/separated) | 507 (20.60) | 23 (13.85) | 61 (16.98) | 300 (20.25) | 24 (19.90) | 99 (38.27) |
| Never married | 311 (15.76) | 45 (27.78) | 79 (21.54) | 171 (14.99) | 5 (3.12) | 11 (3.19) |
| PIR, % |  |  |  |  |  |  |
| <1.3 | 573 (18.25) | 40 (18.15) | 97 (19.57) | 340 (17.62) | 24 (12.13) | 72 (23.36) |
| 1.3 to <3.5 | 822 (36.08) | 52 (33.05) | 119 (32.76) | 510 (37.11) | 40 (32.89) | 101 (40.92) |
| >=3.5 | 674 (45.67) | 54 (48.80) | 128 (47.66) | 389 (45.28) | 43 (54.97) | 60 (35.72) |
| Smoking, % |  |  |  |  |  |  |
| No | 1107 (54.47) | 91 (57.76) | 199 (53.88) | 671 (56.05) | 52 (51.54) | 94 (42.86) |
| Yes | 962 (45.53) | 55 (42.24) | 145 (46.12) | 568 (43.95) | 55 (48.46) | 139 (57.14) |
| Drinking, % |  |  |  |  |  |  |
| No | 691 (28.04) | 43 (21.90) | 102 (24.30) | 445 (30.63) | 19 (13.29) | 82 (32.63) |
| Yes | 1378 (71.96) | 103 (78.10) | 242 (75.70) | 794 (69.37) | 88 (86.71) | 151 (67.37) |
| Physical Activity, % |  |  |  |  |  |  |
| Low physical activity | 768 (31.23) | 32 (18.63) | 97 (26.82) | 483 (33.25) | 41 (29.97) | 115 (40.58) |
| High physical activity | 1301 (68.77) | 114 (81.37) | 247 (73.18) | 756 (66.75) | 66 (70.03) | 118 (59.42) |
| Hypertension, % |  |  |  |  |  |  |
| No | 1153 (60.53) | 146 (100.00) | 344 (100.00) | 606 (51.87) | 3 (1.89) | 54 (27.80) |
| Yes | 916 (39.47) | 0 (0.00) | 0 (0.00) | 633 (48.13) | 104 (98.11) | 179 (72.20) |
| Diabetes, % |  |  |  |  |  |  |
| No | 1575 (82.52) | 145 (99.58) | 333 (97.18) | 928 (80.48) | 61 (71.26) | 108 (53.92) |
| Yes | 494 (17.48) | 1 (0.42) | 11 (2.82) | 311 (19.52) | 46 (28.74) | 125 (46.08) |
| CKD, % |  |  |  |  |  |  |
| No | 1737 (88.69) | 146 (100.00) | 344 (100.00) | 1022 (85.88) | 81 (85.07) | 144 (74.26) |
| Yes | 332 (11.31) | 0 (0.00) | 0 (0.00) | 217 (14.12) | 26 (14.93) | 89 (25.74) |
| Cancer, % |  |  |  |  |  |  |
| No | 1860 (89.40) | 140 (95.41) | 328 (94.33) | 1121 (89.25) | 88 (80.39) | 183 (78.62) |
| Yes | 209 (10.60) | 6 (4.59) | 16 (5.67) | 118 (10.75) | 19 (19.61) | 50 (21.38) |
| Depression, % |  |  |  |  |  |  |
| No | 1895 (93.20) | 132 (93.44) | 320 (94.43) | 1145 (93.45) | 99 (94.95) | 199 (87.70) |
| Yes | 174 (6.80) | 14 (6.56) | 24 (5.57) | 94 (6.55) | 8 (5.05) | 34 (12.30) |
| Mortality Status, % |  |  |  |  |  |  |
| No | 1869 (93.57) | 144 (99.03) | 329 (97.11) | 1147 (94.66) | 90 (90.98) | 159 (74.25) |
| Yes | 200 (6.43) | 2 (0.97) | 15 (2.89) | 92 (5.34) | 17 (9.02) | 74 (25.75) |
| Death CVD, % |  |  |  |  |  |  |
| No | 2007 (98.00) | 146 (100.00) | 341 (99.11) | 1215 (98.70) | 103 (98.45) | 202 (88.40) |
| Yes | 62 (2.00) | 0 (0.00) | 3 (0.89) | 24 (1.30) | 4 (1.55) | 31 (11.60) |
| Death Renal, % |  |  |  |  |  |  |
| No | 2065 (99.93) | 146 (100.00) | 344 (100.00) | 1239 (100.00) | 106 (99.43) | 230 (99.51) |
| Yes | 4 (0.07) | 0 (0.00) | 0 (0.00) | 0 (0.00) | 1 (0.57) | 3 (0.49) |
| Abbreviations: BMI, body mass index; WC, waist circumference; WHtR, waist‑to‑height ratio; WBC, white blood cell count; LYM, lymphocyte percentage; MCV, mean corpuscular volume; RDW, red cell distribution width; PLT, platelet count; HbA1c, glycated hemoglobin; FBG, fasting blood glucose; ALP, alkaline phosphatase; BUN, blood urea nitrogen; SCR, serum creatinine; UA, uric acid; UACR, urine albumin‑to‑creatinine ratio; eGFR, estimated glomerular filtration rate; TC, total cholesterol; HDL, high‑density lipoprotein; TG, triglycerides; CMI, cardiometabolic index; VE, vitamin E; B6, vitamin B6; VC, vitamin C; PHENOAGE, phenotypic age; KDM, KDM biological age; CKMAI, CKM‑specific aging index; PIR, poverty‑to‑income ratio; CKD, chronic kidney disease; CVD, cardiovascular disease. Percentages are weighted to account for complex survey design. | | | | | | |
