# Supplementary material for: A machine learning-derived aging index for risk stratification and mortality prediction in cardiovascular-kidney-metabolic syndrome: A retrospective cohort study
Source: PLoS Med. 2026 Oct 6;23(10):e1005078. doi: 10.1371/journal.pmed.1005078 (PMC13641365; doi:10.1371/journal.pmed.1005078)
Supplement: Supplementary file 9 [file pmed.1005078.s037.docx]

**Supplementary Table 3.** Comparison of CKMAI distribution between the training and testing sets

| Dataset | Mean | SD | Mean ± SD | P value* |
| --- | --- | --- | --- | --- |
| Training (n = 4,827) | 40.949 | 16.586 | 40.95 ± 16.59 |  |
| Testing (n = 2,069) | 41.142 | 16.885 | 41.14 ± 16.89 | 0.745 |

*P value from two‑sample t‑test comparing mean CKMAI between training and testing sets.
