# Supplementary material for: A machine learning-derived aging index for risk stratification and mortality prediction in cardiovascular-kidney-metabolic syndrome: A retrospective cohort study
Source: PLoS Med. 2026 Oct 6;23(10):e1005078. doi: 10.1371/journal.pmed.1005078 (PMC13641365; doi:10.1371/journal.pmed.1005078)
Supplement: Supplementary file 10 [file pmed.1005078.s038.docx]

**Supplementary Table 4.** Complete list of stable features and their bootstrap selection frequencies

| Feature | Freq All Cause | Freq CKM |
| --- | --- | --- |
| Age | 0.908571 | 0.522857 |
| RDW | 0.9 | 0.571429 |
| UACR | 0.891429 | 0.548571 |
| Marital_Status2 | 0.86 | 0.51 |
| PIR3 | 0.852857 | 0.562857 |
| Diabetes1 | 0.848571 | 0.478571 |
| LYM | 0.805714 | 0.432857 |
| ALP | 0.788571 | 0.571429 |
| Education3 | 0.781429 | 0.424286 |
| Ethnicity2 | 0.772857 | 0.485714 |
| Sex1 | 0.752857 | 0.565714 |
| EGFR | 0.747143 | 0.558571 |
| BUN | 0.742857 | 0.561429 |
| Smoking1 | 0.741429 | 0.571429 |
| ALB | 0.712857 | 0.401429 |
| MCV | 0.681429 | 0.56 |
| WBC | 0.615714 | 0.478571 |
| SCR | 0.611429 | 0.518571 |
| PLT | 0.592857 | 0.541429 |
| Sex2 | 0.592857 | 0.392857 |
| Hypertension1 | 0.582857 | 0.521429 |
| Physical_Activity2 | 0.564286 | 0.414286 |
| PIR2 | 0.547143 | 0.472857 |
| Weight | 0.545714 | 0.452857 |
| WC | 0.544286 | 0.512857 |
| BMI | 0.544286 | 0.47 |
| Energy_Intake | 0.524286 | 0.484286 |
| WHtR | 0.524286 | 0.462857 |
| Ethnicity4 | 0.518571 | 0.514286 |
| HbA1c | 0.514286 | 0.532857 |
| UA | 0.5 | 0.408571 |
| Height | 0.497143 | 0.358571 |
| TC | 0.494286 | 0.537143 |
| FBG | 0.487143 | 0.467143 |
| Drinking1 | 0.462857 | 0.534286 |
| Education2 | 0.452857 | 0.434286 |
| VC | 0.427143 | 0.391429 |
| Ethnicity3 | 0.424286 | 0.404286 |
| B6 | 0.411429 | 0.382857 |
| Marital_Status3 | 0.408571 | 0.448571 |
| TG | 0.402857 | 0.365714 |
| VE | 0.381429 | 0.37 |
| TG_HDL | 0.38 | 0.442857 |
| HDL | 0.378571 | 0.475714 |

Freq All Cause: bootstrap selection frequency for all cause mortality; Freq CKM: bootstrap selection frequency for CKM high risk status. Stability selection was performed using 500 bootstrap resamples with the glmnet and survival packages. A selection frequency ≥60% was considered stable. Abbreviations: RDW, red cell distribution width; UACR, urine albumin to creatinine ratio; PIR, poverty to income ratio (suffix 2 = 1.3–<3.5, suffix 3 = ≥3.5); LYM, lymphocyte percentage; ALP, alkaline phosphatase; EGFR, estimated glomerular filtration rate; BUN, blood urea nitrogen; ALB, albumin; MCV, mean corpuscular volume; WBC, white blood cell count; SCR, serum creatinine; PLT, platelet count; WC, waist circumference; BMI, body mass index; WHtR, waist to height ratio; HbA1c, glycated hemoglobin; UA, uric acid; TC, total cholesterol; FBG, fasting blood glucose; TG, triglycerides; HDL, high density lipoprotein; VE, vitamin E; B6, vitamin B6; VC, vitamin C. Categorical variable codes: Marital_Status2 = single (widowed/divorced/separated), Marital_Status3 = never married; Education2 = high school or equivalent, Education3 = college or above; Ethnicity2 = Non Hispanic White, Ethnicity3 = Non Hispanic Black, Ethnicity4 = other; Sex1 = male, Sex2 = female; Diabetes1 = yes; Smoking1 = yes; Hypertension1 = yes; Drinking1 = yes; Physical_Activity2 = high physical activity. Detailed variable definitions are provided in the Methods section.
