# Supplementary material for: A machine learning-derived aging index for risk stratification and mortality prediction in cardiovascular-kidney-metabolic syndrome: A retrospective cohort study
Source: PLoS Med. 2026 Oct 6;23(10):e1005078. doi: 10.1371/journal.pmed.1005078 (PMC13641365; doi:10.1371/journal.pmed.1005078)
Supplement: Supplementary file 11 [file pmed.1005078.s039.docx]

**Supplementary Table 5.** DeLong test P‑values for comparisons of time‑dependent AUCs in the testing set

| Outcome | Time (years) | CKMAI vs. PhenoAge | CKMAI vs. KDM | CKMAI vs. CMI | PhenoAge vs. KDM | PhenoAge vs. CMI | KDM vs. CMI |
| --- | --- | --- | --- | --- | --- | --- | --- |
| **All‑cause mortality** | 3 | 0.003** | <0.001*** | <0.001*** | <0.001*** | <0.001*** | <0.001*** |
|  | 5 | <0.001*** | <0.001*** | <0.001*** | 0.003** | <0.001*** | <0.001*** |
|  | 10 | <0.001*** | <0.001*** | <0.001*** | <0.001*** | <0.001*** | <0.001*** |
| **Cardiovascular mortality** | 3 | 0.073 | 0.002** | <0.001*** | 0.018* | <0.001*** | 0.020* |
|  | 5 | <0.001*** | <0.001*** | <0.001*** | <0.001*** | <0.001*** | <0.001*** |
|  | 10 | 0.003** | <0.001*** | <0.001*** | 0.004** | <0.001*** | <0.001*** |
| **High‑risk CKM status** | – | 0.002** | <0.001*** | <0.001*** | – | – | – |

Abbreviations: CKM, cardiovascular kidney metabolic. P values were obtained from DeLong tests for paired comparisons of time dependent AUCs using the timeROC package. Significance codes: ***P < 0.001; **P < 0.01; *P < 0.05. All comparisons were performed on the independent testing set. The statistical test used was DeLong's method for comparing correlated ROC curves.
