# Supplementary material for: A machine learning-derived aging index for risk stratification and mortality prediction in cardiovascular-kidney-metabolic syndrome: A retrospective cohort study
Source: PLoS Med. 2026 Oct 6;23(10):e1005078. doi: 10.1371/journal.pmed.1005078 (PMC13641365; doi:10.1371/journal.pmed.1005078)
Supplement: Supplementary file 12 [file pmed.1005078.s040.docx]

**Supplementary Table 6.** Predictive performance of CKMAI, PhenoAge, KDM, and CMI in the training set

| Outcome | Metric | CKMAI | PhenoAge | KDM | CMI |
| --- | --- | --- | --- | --- | --- |
| **All‑cause mortality** | Time‑dependent AUC (95% CI), 3y | 0.883 (0.864,0.914) | 0.820 (0.796,0.852) | 0.742 (0.688,0.780) | 0.520 (0.475,0.585) |
|  | Time‑dependent AUC (95% CI), 5y | 0.881 (0.859,0.902) | 0.835 (0.810,0.860) | 0.763 (0.733,0.799) | 0.511 (0.470,0.549) |
|  | Time‑dependent AUC (95% CI), 10y | 0.897 (0.879,0.913) | 0.852 (0.833,0.872) | 0.771 (0.741,0.792) | 0.514 (0.483,0.547) |
| **Cardiovascular mortality** | Time‑dependent AUC (95% CI), 3y | 0.877 (0.819,0.927) | 0.792 (0.729,0.860) | 0.705 (0.620,0.827) | 0.559 (0.467,0.637) |
|  | Time‑dependent AUC (95% CI), 5y | 0.905 (0.873,0.933) | 0.854 (0.817,0.884) | 0.765 (0.708,0.834) | 0.549 (0.497,0.586) |
|  | Time‑dependent AUC (95% CI), 10y | 0.907 (0.881,0.927) | 0.871 (0.847,0.892) | 0.782 (0.738,0.817) | 0.536 (0.494,0.587) |
| **High‑risk CKM status** | AUC (95% CI) | 0.767 (0.751,0.783) | 0.735 (0.718,0.752) | 0.715 (0.696,0.734) | 0.605 (0.585,0.626) |

Abbreviations: CI, confidence interval; AUC, area under the curve; CKM, cardiovascular kidney metabolic; PHENOAGE, phenotypic age; KDM, KDM biological age; CKMAI, CKM specific aging index; CMI, cardiometabolic index. All AUC values are point estimates with 95% confidence intervals (CI). 95% CIs were derived from 200 bootstrap resamples. NRI and IDI were not calculated for the training set as they are typically reserved for independent validation. Time dependent AUCs were calculated using the timeROC package.
