# Supplementary material for: A machine learning-derived aging index for risk stratification and mortality prediction in cardiovascular-kidney-metabolic syndrome: A retrospective cohort study
Source: PLoS Med. 2026 Oct 6;23(10):e1005078. doi: 10.1371/journal.pmed.1005078 (PMC13641365; doi:10.1371/journal.pmed.1005078)
Supplement: Supplementary file 13 [file pmed.1005078.s041.docx]

**Supplementary Table 7.** Summary of sensitivity analyses

| Sensitivity analysis | HR (95% CI) for all‑cause mortality |
| --- | --- |
| Unweighted Cox regression | 1.092 (1.088,1.096) |
| 5‑year restricted follow‑up | 1.096 (1.085,1.108) |
| Exclusion of baseline CVD/CKD | 1.097 (1.084,1.110) |
| Primary weighted analysis (reference) | 1.097 (1.089,1.106) |

Abbreviations: HR, hazard ratio; CI, confidence interval; CVD, cardiovascular disease; CKD, chronic kidney disease. All HRs are derived from weighted Cox proportional hazards models, with the primary weighted analysis as the reference. 95% CIs were calculated using Wald-type confidence intervals. P values for all HRs were <0.001 based on Wald tests. The unweighted analysis did not apply NHANES survey weights. The 5 year restricted follow up analysis censored participants at 5 years. The exclusion analysis removed participants with baseline CVD or CKD.
