# Supplementary material for: A machine learning-derived aging index for risk stratification and mortality prediction in cardiovascular-kidney-metabolic syndrome: A retrospective cohort study
Source: PLoS Med. 2026 Oct 6;23(10):e1005078. doi: 10.1371/journal.pmed.1005078 (PMC13641365; doi:10.1371/journal.pmed.1005078)
Supplement: Supplementary file 14 [file pmed.1005078.s042.docx]

**Supplementary Table 8.** Mediation analysis of depression score on the associations between CKMAI and outcomes

| Outcome | Effect | Estimate (95% CI) | P value | Proportion mediated (95% CI) |
| --- | --- | --- | --- | --- |
| **All‑cause mortality** | ACME | –26.62 (–50.90, –10.07) | <0.001 | 5.6% (2.3%–9.0%) |
|  | ADE | –473.40 (–795.25, –252.64) | <0.001 |  |
|  | Total effect | –500.03 (–842.60, –269.44) | <0.001 |  |
| **Cardiovascular mortality** | ACME | –52.94 (–171.45, –10.34) | <0.001 | 8.2% (2.8%–22.0%) |
|  | ADE | –547.85 (–1433.45, –115.04) | <0.001 |  |
|  | Total effect | –600.79 (–1585.74, –139.34) | <0.001 |  |
| **High‑risk CKM status** | ACME | 1.18e-04 (4.73e-05, 2.40e-04)^a^ | <0.001 | 11.1% (6.7%–24.0%) |
|  | ADE | 8.08e-04 (5.79e-04, 1.06e-03) | <0.001 |  |
|  | Total effect | 9.26e-04 (6.26e-04, 1.28e-03) | <0.001 |  |

Abbreviations: ACME, average causal mediation effect (indirect effect); ADE, average direct effect; CI, confidence interval; CKM, cardiovascular kidney metabolic. Estimates for all cause and cardiovascular mortality are on the hazard scale; estimates for high risk CKM status are on the probability scale. All models adjusted for sex, ethnicity, education, and age. P values were derived from weighted bootstrap mediation analysis with 1,000 replicates using the mediation package in R, with the quasi Bayesian Monte Carlo method for confidence interval estimation. The proportion mediated is calculated as ACME/(ACME+ADE). ^a^ The 95% CI upper bound is reported as 2.40e-04 based on the quasi Bayesian output; the original output showed 0.00 due to rounding, but the true upper bound is non zero as indicated by the proportion mediated confidence interval.
