# Supplementary material for: A machine learning-derived aging index for risk stratification and mortality prediction in cardiovascular-kidney-metabolic syndrome: A retrospective cohort study
Source: PLoS Med. 2026 Oct 6;23(10):e1005078. doi: 10.1371/journal.pmed.1005078 (PMC13641365; doi:10.1371/journal.pmed.1005078)
Supplement: Supplementary file 15 [file pmed.1005078.s043.docx]

**Supplementary Table 9.** Baseline characteristics of the study population overall and by survey periods (NHANES 2005–2010 and 2011–2018)

| **Variable** | **Total (n = 6896)** | **2005-2010 (n=3900)** | **2011-2018 (n=2996)** |
| --- | --- | --- | --- |
| Age, Mean (SD), years | 47.87 (0.37) | 48.00 (0.53) | 47.73 (0.53) |
| BMI, Mean (SD), kg/m² | 29.20 (0.13) | 28.77 (0.13) | 29.68 (0.22) |
| WC, Mean (SD), cm | 99.87 (0.34) | 98.86 (0.36) | 100.95 (0.57) |
| Depression score, Mean (SD) | 2.91 (0.07) | 2.78 (0.09) | 3.06 (0.10) |
| WBC, Mean (SD), ×1000 cells/μL | 6.81 (0.04) | 6.76 (0.05) | 6.85 (0.06) |
| LYM, Mean (SD), % | 30.14 (0.14) | 30.05 (0.19) | 30.24 (0.20) |
| MCV, Mean (SD), fL | 89.42 (0.11) | 89.54 (0.16) | 89.29 (0.16) |
| RDW, Mean (SD), % | 13.11 (0.02) | 12.67 (0.03) | 13.58 (0.03) |
| HbA1c, Mean (SD), % | 5.62 (0.01) | 5.60 (0.02) | 5.65 (0.02) |
| ALB, Mean (SD), g/dL | 4.23 (0.01) | 4.26 (0.01) | 4.20 (0.02) |
| ALP, Mean (SD), U/L | 69.06 (0.40) | 67.53 (0.50) | 70.70 (0.64) |
| BUN, Mean (SD), mg/dL | 13.85 (0.10) | 13.19 (0.11) | 14.56 (0.16) |
| UACR, Mean (SD), mg/g | 28.32 (2.27) | 30.61 (3.61) | 25.85 (2.69) |
| eGFR, Mean (SD), mL/min/1.73m² | 96.63 (0.44) | 96.15 (0.57) | 97.15 (0.67) |
| TG, Mean (SD), mg/dL | 128.05 (1.70) | 141.22 (2.41) | 113.88 (2.25) |
| HDL, Mean (SD), mg/dL | 53.92 (0.33) | 53.37 (0.40) | 54.52 (0.52) |
| CMI, Mean (SD) | 1.74 (0.04) | 1.91 (0.05) | 1.56 (0.05) |
| PHENOAGE, Mean (SD) | 57.85 (0.40) | 56.32 (0.56) | 59.51 (0.57) |
| KDM, Mean (SD) | 42.56 (0.36) | 41.59 (0.49) | 43.61 (0.53) |
| CKMAI, Mean (SD) | 40.14 (0.35) | 38.78 (0.46) | 41.61 (0.52) |
| Weight, Mean (SD), kg | 84.18 (0.37) | 83.36 (0.41) | 85.07 (0.63) |
| Height, Mean (SD), cm | 169.53 (0.17) | 169.96 (0.22) | 169.06 (0.27) |
| VE, Mean (SD), mg | 0.51 (0.02) | 0.45 (0.02) | 0.57 (0.04) |
| B6, Mean (SD), mg | 0.19 (0.01) | 0.20 (0.01) | 0.19 (0.01) |
| VC, Mean (SD), mg | 5.12 (0.31) | 5.69 (0.40) | 4.50 (0.47) |
| TC, Mean (SD), mg/dL | 193.80 (0.82) | 197.84 (0.92) | 189.45 (1.33) |
| PLT, Mean (SD), ×1000 cells/μL | 247.71 (1.17) | 258.03 (1.44) | 236.60 (1.81) |
| FBG, Mean (SD), mg/dL | 107.71 (0.40) | 105.96 (0.52) | 109.60 (0.60) |
| SCR, Mean (SD), mg/dL | 0.88 (0.00) | 0.89 (0.00) | 0.86 (0.01) |
| UA, Mean (SD), mg/dL | 5.56 (0.02) | 5.64 (0.03) | 5.48 (0.03) |
| Energy Intake, Mean (SD), kcal | 2126.98 (12.69) | 2150.85 (19.19) | 2101.28 (16.72) |
| Sex, n (%) |  |  |  |
| Male | 3603 (51.67) | 2065 (52.82) | 1538 (50.42) |
| Female | 3293 (48.33) | 1835 (47.18) | 1458 (49.58) |
| Ethnicity, n (%) |  |  |  |
| Mexican American | 1125 (7.76) | 663 (7.26) | 462 (8.29) |
| Non-Hispanic White | 3219 (70.66) | 2061 (73.47) | 1158 (67.63) |
| Non-Hispanic Black | 1255 (9.54) | 681 (9.80) | 574 (9.27) |
| Other | 1297 (12.04) | 495 (9.47) | 802 (14.81) |
| Education, n (%) |  |  |  |
| Less than high school | 1565 (14.15) | 1006 (17.12) | 559 (10.95) |
| High school or equivalent | 1668 (24.60) | 947 (23.91) | 721 (25.34) |
| College or above | 3663 (61.25) | 1947 (58.96) | 1716 (63.70) |
| Marital Status, n (%) |  |  |  |
| Married or living with a partner | 4268 (65.29) | 2448 (66.42) | 1820 (64.07) |
| Single (widowed/divorced/separated) | 1551 (18.55) | 893 (18.31) | 658 (18.80) |
| Never married | 1077 (16.16) | 559 (15.27) | 518 (17.12) |
| PIR, n (%) |  |  |  |
| <1.3 | 1941 (18.40) | 1095 (17.65) | 846 (19.21) |
| 1.3 to <3.5 | 2723 (36.53) | 1492 (35.95) | 1231 (37.15) |
| >=3.5 | 2232 (45.07) | 1313 (46.40) | 919 (43.63) |
| Smoking, n (%) |  |  |  |
| No | 3624 (52.74) | 2014 (52.04) | 1610 (53.50) |
| Yes | 3272 (47.26) | 1886 (47.96) | 1386 (46.50) |
| Drinking, n (%) |  |  |  |
| No | 2235 (27.90) | 1069 (23.34) | 1166 (32.81) |
| Yes | 4661 (72.10) | 2831 (76.66) | 1830 (67.19) |
| Physical Activity, n (%) |  |  |  |
| Low physical activity | 2586 (31.98) | 1509 (33.89) | 1077 (29.93) |
| High physical activity | 4310 (68.02) | 2391 (66.11) | 1919 (70.07) |
| Hypertension, n (%) |  |  |  |
| No | 3767 (60.09) | 2068 (58.02) | 1699 (62.31) |
| Yes | 3129 (39.91) | 1832 (41.98) | 1297 (37.69) |
| Diabetes, n (%) |  |  |  |
| No | 5313 (83.12) | 3026 (83.23) | 2287 (83.00) |
| Yes | 1583 (16.88) | 874 (16.77) | 709 (17.00) |
| CKD, n (%) |  |  |  |
| No | 5763 (87.80) | 3246 (87.48) | 2517 (88.15) |
| Yes | 1133 (12.20) | 654 (12.52) | 479 (11.85) |
| Cancer, n (%) |  |  |  |
| No | 6218 (90.33) | 3502 (90.38) | 2716 (90.29) |
| Yes | 678 (9.67) | 398 (9.62) | 280 (9.71) |
| Depression, n (%) |  |  |  |
| No | 6328 (93.05) | 3590 (93.66) | 2738 (92.39) |
| Yes | 568 (6.95) | 310 (6.34) | 258 (7.61) |
| Mortality Status, n (%) |  |  |  |
| No | 6236 (93.45) | 3317 (89.10) | 2919 (98.13) |
| Yes | 660 (6.55) | 583 (10.90) | 77 (1.87) |
| Death CVD, n (%) |  |  |  |
| No | 6695 (97.91) | 3721 (96.50) | 2974 (99.43) |
| Yes | 201 (2.09) | 179 (3.50) | 22 (0.57) |
| CKM, n (%) |  |  |  |
| Stage 0 | 506 (9.23) | 247 (8.60) | 259 (9.91) |
| Stage 1 | 1118 (19.19) | 524 (14.64) | 594 (24.08) |
| Stage 2 | 4105 (57.86) | 2416 (61.04) | 1689 (54.43) |
| Stage 3 | 374 (5.07) | 256 (6.61) | 118 (3.41) |
| Stage 4 | 793 (8.65) | 457 (9.11) | 336 (8.16) |
| CKM high, n (%) |  |  |  |
| No | 5729 (86.28) | 3187 (84.29) | 2542 (88.43) |
| Yes | 1167 (13.72) | 713 (15.71) | 454 (11.57) |

Values are presented as mean (standard deviation) for continuous variables and n (%) for categorical variables, accounting for the complex survey design with appropriate sampling weights. The total analytic sample comprised 6,896 participants (3,900 in the 2005–2010 cycle and 2,996 in the 2011–2018 cycle). Abbreviations: BMI, body mass index; WC, waist circumference; WBC, white blood cell count; LYM, lymphocyte percentage; MCV, mean corpuscular volume; RDW, red cell distribution width; HbA1c, glycated hemoglobin; ALB, albumin; ALP, alkaline phosphatase; BUN, blood urea nitrogen; UACR, urine albumin‑to‑creatinine ratio; eGFR, estimated glomerular filtration rate; TG, triglycerides; HDL, high‑density lipoprotein; CMI, cardiometabolic index; PHENOAGE, phenotypic age; KDM, Klemera–Doubal method biological age; CKMAI, composite kidney–metabolic aging index; VE, vitamin E; B6, vitamin B6; VC, vitamin C; TC, total cholesterol; PLT, platelet count; FBG, fasting blood glucose; SCR, serum creatinine; UA, uric acid; PIR, poverty‑to‑income ratio (categories: <1.3, 1.3–<3.5, ≥3.5); CKM, cardiovascular–kidney–metabolic health stages; CKM high, high‑risk CKM status. Detailed definitions of all variables are provided in the Methods section.
