# Supplementary material for: A machine learning-derived aging index for risk stratification and mortality prediction in cardiovascular-kidney-metabolic syndrome: A retrospective cohort study
Source: PLoS Med. 2026 Oct 6;23(10):e1005078. doi: 10.1371/journal.pmed.1005078 (PMC13641365; doi:10.1371/journal.pmed.1005078)
Supplement: Supplementary file 16 [file pmed.1005078.s044.docx]

**Supplementary Table 10.** C‑index for all‑cause and cardiovascular mortality, and AUC for CKM high‑risk status in the test dataset

| Indicator | All‑cause mortality (C‑index) | Cardiovascular mortality (C‑index) | CKM high‑risk (AUC) |
| --- | --- | --- | --- |
| **CKMAI** | 0.837 (0.795,0.878) | 0.881 (0.814,0.947) | 0.804 (0.784,0.825) |
| **PHENOAGE** | 0.774 (0.729,0.819) | 0.840 (0.779,0.902) | 0.758 (0.736,0.781) |
| **KDM** | 0.743 (0.692,0.793) | 0.803 (0.727,0.880) | 0.734 (0.709,0.758) |
| **CMI** | 0.504 (0.445,0.564) | 0.587 (0.473,0.702) | 0.611 (0.583,0.638) |

Data are presented as estimate (95% confidence interval). For mortality outcomes (time to event data), Harrell's C index is reported; for CKM high risk status (binary outcome), the AUC is reported. All estimates were derived from the test set (NHANES 2011–2018 for mortality outcomes, and the corresponding validation set for CKM high risk). 95% CIs were derived from 200 bootstrap resamples. Abbreviations: CKMAI, composite kidney--metabolic aging index; PHENOAGE, phenotypic age; KDM, Klemera--Doubal method biological age; CMI, cardiometabolic index; CKM, cardiovascular--kidney--metabolic.
