# Supplementary material for: A machine learning-derived aging index for risk stratification and mortality prediction in cardiovascular-kidney-metabolic syndrome: A retrospective cohort study
Source: PLoS Med. 2026 Oct 6;23(10):e1005078. doi: 10.1371/journal.pmed.1005078 (PMC13641365; doi:10.1371/journal.pmed.1005078)
Supplement: Supplementary file 17 [file pmed.1005078.s045.docx]

**Supplementary Table 11.** Temporal validation of CKMAI and other aging indices across different NHANES cycles (fixed,weight analysis)

| **Period** | **Standardization approach** | **Index** |  | **C,index (all,cause)** | **AUC₃ (all,cause)** | **AUC₅ (all,cause)** | **C,index (CVD)** | **AUC₃ (CVD)** | **AUC₅ (CVD)** | **AUC (CKM≥3)** |
| --- | --- | --- | --- | --- | --- | --- | --- | --- | --- | --- |
| **2005,2010 (Early)** | Original training set | CKMAI |  | 0.842 (0.827,0.854) | 0.869 (0.842,0.900) | 0.865 (0.836,0.889) | 0.854 (0.828,0.875) | 0.856 (0.789,0.919) | 0.876 (0.836,0.908) | 0.755 (0.736,0.775) |
|  | Re,standardized using late period | CKMAI |  | 0.843 (0.828,0.855) | 0.870 (0.844,0.899) | 0.866 (0.837,0.889) | 0.854 (0.830,0.876) | 0.859 (0.795,0.920) | 0.877 (0.837,0.910) | 0.756 (0.737,0.775) |
|  | Direct calculation | PhenoAge |  | 0.832 (0.817,0.846) | 0.861 (0.830,0.886) | 0.857 (0.832,0.876) | 0.854 (0.832,0.875) | 0.807 (0.750,0.863) | 0.859 (0.831,0.889) | 0.741 (0.721,0.766) |
|  | Direct calculation | KDM |  | 0.755 (0.738,0.770) | 0.743 (0.695,0.796) | 0.773 (0.738,0.806) | 0.768 (0.734,0.807) | 0.650 (0.533,0.754) | 0.749 (0.683,0.807) | 0.709 (0.688,0.730) |
|  | Direct calculation | CMI |  | 0.482 (0.470,0.542) | 0.525 (0.475,0.584) | 0.524 (0.484,0.562) | 0.474 (0.457,0.565) | 0.533 (0.436,0.640) | 0.546 (0.485,0.603) | 0.592 (0.571,0.615) |
| **2011,2018 (Late)** | Original training set | CKMAI |  | 0.808 (0.755,0.853) | 0.816 (0.766,0.859) | 0.906 (0.708,1.000) | 0.881 (0.826,0.927) | 0.864 (0.772,0.935) | 0.962 (0.813,1.000) | 0.775 (0.753,0.798) |
|  | Re,standardized using early period | CKMAI |  | 0.810 (0.756,0.854) | 0.817 (0.768,0.861) | 0.898 (0.705,1.000) | 0.882 (0.827,0.926) | 0.865 (0.775,0.936) | 0.958 (0.798,1.000) | 0.776 (0.754,0.799) |
|  | Direct calculation | PhenoAge |  | 0.774 (0.719,0.818) | 0.790 (0.727,0.837) | 0.883 (0.670,0.999) | 0.835 (0.758,0.902) | 0.851 (0.757,0.922) | 0.947 (0.783,1.000) | 0.758 (0.738,0.781) |
|  | Direct calculation | KDM |  | 0.742 (0.691,0.796) | 0.761 (0.698,0.815) | 0.924 (0.811,0.994) | 0.830 (0.741,0.897) | 0.867 (0.765,0.945) | 0.965 (0.839,1.000) | 0.734 (0.710,0.757) |
|  | Direct calculation | CMI |  | 0.503 (0.461,0.573) | 0.478 (0.416,0.548) | 0.355 (0.162,0.643) | 0.591 (0.441,0.703) | 0.583 (0.463,0.714) | 0.475 (0.224,0.743) | 0.611 (0.582,0.637) |

Original training set: CKMAI calculated with weights and standardization parameters derived from the 70% random split of NHANES 2005 2018. Re standardized using late/early period: CKMAI calculated with fixed original weights but using the means and SDs from the opposite period (late or early) for standardization. Direct calculation: indices computed directly on the raw data without any external standardization. Values in parentheses are 95% confidence intervals derived from 200 bootstrap resamples. CKMAI, CKM specific aging index; PHENOAGE, phenotypic age; KDM, KDM biological age; CMI, cardiometabolic index; CKM, cardiovascular kidney metabolic; CVD, cardiovascular disease; AUC₃, time dependent AUC at 3 years; AUC₅, time dependent AUC at 5 years; CI, confidence interval.
