# Supplementary material for: A machine learning-derived aging index for risk stratification and mortality prediction in cardiovascular-kidney-metabolic syndrome: A retrospective cohort study
Source: PLoS Med. 2026 Oct 6;23(10):e1005078. doi: 10.1371/journal.pmed.1005078 (PMC13641365; doi:10.1371/journal.pmed.1005078)
Supplement: Supplementary file 18 [file pmed.1005078.s046.docx]

**Supplementary Table 12.** AUC for predicting CKM high-risk status (CKM≥3) in the hospital validation cohort

| Index | AUC (95% CI) |
| --- | --- |
| **CKMAI** | 0.789 (0.730,0.847) |

CKM, cardiovascular–kidney–metabolic; CKMAI, composite kidney–metabolic aging index;
