# Supplementary material for: A machine learning-derived aging index for risk stratification and mortality prediction in cardiovascular-kidney-metabolic syndrome: A retrospective cohort study
Source: PLoS Med. 2026 Oct 6;23(10):e1005078. doi: 10.1371/journal.pmed.1005078 (PMC13641365; doi:10.1371/journal.pmed.1005078)
Supplement: Supplementary file 19 [file pmed.1005078.s047.docx]

**Supplementary Table 13.** Baseline characteristics of the hospital validation cohort stratified by CKM stage

| Variable | Total  (n = 261) | Stage 0  (n = 138) | Stage 1  (n = 39) | Stage 2  (n = 29) | Stage 3  (n = 11) | Stage 4  (n = 44) |
| --- | --- | --- | --- | --- | --- | --- |
| ALB, g/dL, Mean ± SD | 4.21 ± 0.35 | 4.25 ± 0.37 | 4.18 ± 0.29 | 4.20 ± 0.30 | 4.15 ± 0.32 | 4.13 ± 0.34 |
| SCR, mg/dL, Mean ± SD | 0.93 ± 0.41 | 0.88 ± 0.39 | 0.91 ± 0.41 | 0.78 ± 0.37 | 1.19 ± 0.48 | 1.13 ± 0.38 |
| CKMAI, Mean ± SD | 41.41 ± 18.49 | 35.74 ± 18.75 | 41.37 ± 17.21 | 41.97 ± 14.22 | 47.00 ± 13.12 | 57.46 ± 11.81 |
| Weight, kg, Median (Q1, Q3) | 76.01  (65.11, 90.75) | 73.40  (63.09, 86.59) | 78.87  (72.05, 98.54) | 73.69  (57.76, 85.36) | 88.66  (79.60, 99.82) | 78.00  (67.87, 96.94) |
| Height, cm, Median (Q1, Q3) | 165.33 (157.97, 172.94) | 166.08 (159.38, 172.59) | 167.30 (160.74, 175.95) | 157.17 (153.01, 171.16) | 175.63 (168.22, 178.95) | 163.12 (156.24, 167.25) |
| BMI, kg/m², Median (Q1, Q3) | 27.34  (24.03, 32.31) | 26.51  (23.43, 31.34) | 28.16  (23.43, 32.54) | 27.16  (23.90, 32.06) | 33.68  (29.27, 34.58) | 29.80  (25.86, 35.59) |
| WC, cm, Median (Q1, Q3) | 98.27  (87.55, 108.33) | 93.61  (84.16, 104.75) | 101.07  (91.45, 112.46) | 96.05  (86.30, 104.62) | 108.27  (105.66, 111.59) | 104.16  (93.88, 121.20) |
| HDL, mg/dL, Median (Q1, Q3) | 50.71  (41.53, 62.48) | 51.41  (41.40, 63.75) | 47.73  (39.36, 56.55) | 51.10  (44.18, 68.79) | 42.45  (35.72, 52.34) | 50.00  (42.55, 61.05) |
| TG, mg/dL, Median (Q1, Q3) | 112.84  (78.51, 153.76) | 106.22  (77.72, 152.65) | 88.02  (70.68, 141.99) | 131.73  (91.43, 163.26) | 191.79  (117.13, 309.74) | 121.27  (72.42, 151.47) |
| WBC, ×10³/μL, Median (Q1, Q3) | 7.12  (5.87, 8.49) | 7.28  (6.05, 8.66) | 7.51  (6.25, 8.78) | 6.70  (5.29, 7.12) | 6.46  (5.82, 7.30) | 6.83  (5.68, 8.11) |
| LYM, %, Median (Q1, Q3) | 30.66  (23.66, 35.74) | 30.66  (23.61, 36.25) | 32.04  (25.02, 35.32) | 32.09  (26.18, 37.74) | 26.47  (23.96, 43.05) | 27.16  (22.50, 32.53) |
| MCV, fL, Median (Q1, Q3) | 89.23  (86.67, 93.41) | 89.00  (85.50, 92.22) | 89.96  (88.22, 93.44) | 89.29  (86.91, 93.36) | 90.90  (85.00, 93.58) | 89.91  (87.68, 95.43) |
| RDW, %, Median (Q1, Q3) | 13.18  (12.59, 13.93) | 13.18  (12.58, 13.91) | 13.13  (12.51, 13.61) | 13.18  (12.71, 13.53) | 13.25  (12.87, 14.16) | 13.13  (12.55, 14.95) |
| ALP, U/L, Median (Q1, Q3) | 65.74  (55.17, 80.09) | 63.69  (52.76, 79.96) | 65.39  (54.51, 76.88) | 67.57  (59.19, 79.40) | 60.71  (50.60, 75.02) | 71.22  (60.26, 91.11) |
| BUN, mg/dL, Median (Q1, Q3) | 13.02  (10.21, 16.46) | 12.50  (9.60, 14.77) | 14.10  (11.65, 17.41) | 11.63  (10.17, 14.54) | 17.52  (9.88, 22.77) | 15.86  (13.29, 18.81) |
| Age, years, Median (Q1, Q3) | 54.00  (38.00, 69.00) | 45.00  (29.25, 62.00) | 55.00  (42.00, 69.50) | 60.00  (45.00, 67.00) | 59.00  (47.00, 65.00) | 71.00  (64.75, 78.00) |
| UACR, mg/g, Median (Q1, Q3) | 12.82  (3.93, 22.97) | 10.90  (3.73, 20.61) | 15.10  (4.48, 25.00) | 10.78  (4.70, 18.52) | 12.56  (2.73, 21.40) | 16.51  (4.71, 30.87) |
| eGFR, mL/min/1.73m², Median (Q1, Q3) | 84.46  (63.66,  111.91) | 93.41  (70.36,  118.23) | 82.46  (63.77,  109.17) | 87.68  (71.07,  110.08) | 72.27  (65.26,  92.95) | 60.58  (48.13,  77.47) |
| Sex, n (%) |  |  |  |  |  |  |
| Male | 120 (45.98) | 62 (44.93) | 17 (43.59) | 9 (31.03) | 10 (90.91) | 22 (50.00) |
| Female | 141 (54.02) | 76 (55.07) | 22 (56.41) | 20 (68.97) | 1 (9.09) | 22 (50.00) |
| Smoking, n (%) |  |  |  |  |  |  |
| No | 155 (59.39) | 86 (62.32) | 19 (48.72) | 24 (82.76) | 7 (63.64) | 19 (43.18) |
| Yes | 106 (40.61) | 52 (37.68) | 20 (51.28) | 5 (17.24) | 4 (36.36) | 25 (56.82) |
| Education, n (%) |  |  |  |  |  |  |
| Less than high school | 66 (25.29) | 30 (21.74) | 8 (20.51) | 10 (34.48) | 2 (18.18) | 16 (36.36) |
| High school or equivalent | 55 (21.07) | 35 (25.36) | 6 (15.38) | 1 (3.45) | 4 (36.36) | 9 (20.45) |
| College or above | 140 (53.64) | 73 (52.90) | 25 (64.10) | 18 (62.07) | 5 (45.45) | 19 (43.18) |
| Marital Status, n (%) |  |  |  |  |  |  |
| Married/cohabiting | 141 (54.02) | 69 (50.00) | 21 (53.85) | 23 (79.31) | 8 (72.73) | 20 (45.45) |
| Single/divorced/separated | 65 (24.90) | 31 (22.46) | 7 (17.95) | 5 (17.24) | 1 (9.09) | 21 (47.73) |
| Never married | 55 (21.07) | 38 (27.54) | 11 (28.21) | 1 (3.45) | 2 (18.18) | 3 (6.82) |
| SES, n (%) |  |  |  |  |  |  |
| Low | 87 (33.33) | 42 (30.43) | 10 (25.64) | 8 (27.59) | 6 (54.55) | 21 (47.73) |
| Medium | 93 (35.63) | 58 (42.03) | 12 (30.77) | 7 (24.14) | 1 (9.09) | 15 (34.09) |
| High | 81 (31.03) | 38 (27.54) | 17 (43.59) | 14 (48.28) | 4 (36.36) | 8 (18.18) |
| Diabetes, n (%) |  |  |  |  |  |  |
| No | 201 (77.01) | 118 (85.51) | 36 (92.31) | 21 (72.41) | 7 (63.64) | 19 (43.18) |
| Yes | 60 (22.99) | 20 (14.49) | 3 (7.69) | 8 (27.59) | 4 (36.36) | 25 (56.82) |

Continuous variables are presented as mean ± SD for approximately normally distributed variables (ALB, SCR, PHENOAGE, CKMAI) and as median (Q1, Q3) for skewed variables (all others). Categorical variables are presented as n (column percentage). Percentages may not sum to 100% due to rounding. Abbreviations: BMI, body mass index; WC, waist circumference; WBC, white blood cell count; LYM, lymphocyte percentage; MCV, mean corpuscular volume; RDW, red cell distribution width; ALB, albumin; ALP, alkaline phosphatase; BUN, blood urea nitrogen; SCR, serum creatinine; UACR, urine albumin to creatinine ratio; eGFR, estimated glomerular filtration rate; TG, triglycerides; HDL, high density lipoprotein; CKMAI, CKM specific aging index; SES, socioeconomic status.
