# Supplementary material for: A machine learning-derived aging index for risk stratification and mortality prediction in cardiovascular-kidney-metabolic syndrome: A retrospective cohort study
Source: PLoS Med. 2026 Oct 6;23(10):e1005078. doi: 10.1371/journal.pmed.1005078 (PMC13641365; doi:10.1371/journal.pmed.1005078)
Supplement: Supplementary file 20 [file pmed.1005078.s048.docx]

**Supplementary Table 14.** Normality assessment of continuous variables in the hospital validation cohor

| Variable | Abbreviation | N | W statistic | P value | Normality assumption |
| --- | --- | --- | --- | --- | --- |
| Albumin, g/dL | ALB | 261 | 0.9925 | 0.207 | Yes |
| Serum creatinine, mg/dL | SCR | 261 | 0.9935 | 0.321 | Yes |
| CKMAI | CKMAI | 261 | 0.9916 | 0.139 | Yes |
| Body weight, kg | Weight | 261 | 0.9287 | <0.001 | No |
| Height, cm | Height | 261 | 0.9882 | 0.032 | No |
| BMI, kg/m² | BMI | 261 | 0.9187 | <0.001 | No |
| Waist circumference, cm | WC | 261 | 0.9579 | <0.001 | No |
| HDL‑C, mg/dL | HDL | 261 | 0.9722 | <0.001 | No |
| Triglycerides, mg/dL | TG | 261 | 0.7478 | <0.001 | No |
| WBC, ×10³/μL | WBC | 261 | 0.9796 | <0.001 | No |
| Lymphocyte, % | LYM | 261 | 0.9791 | <0.001 | No |
| MCV, fL | MCV | 261 | 0.9480 | <0.001 | No |
| RDW, % | RDW | 261 | 0.8039 | <0.001 | No |
| ALP, U/L | ALP | 261 | 0.7270 | <0.001 | No |
| BUN, mg/dL | BUN | 261 | 0.8463 | <0.001 | No |
| Age, years | Age | 261 | 0.9431 | <0.001 | No |
| UACR, mg/g | UACR | 261 | 0.1403 | <0.001 | No |
| eGFR, mL/min/1.73m² | eGFR | 261 | 0.9878 | 0.026 | No |

ALB, albumin; SCR, serum creatinine; CKMAI, composite kidney–metabolic aging index; BMI, body mass index; WC, waist circumference; HDL, high‑density lipoprotein cholesterol; TG, triglycerides; WBC, white blood cell count; LYM, lymphocyte percentage; MCV, mean corpuscular volume; RDW, red cell distribution width; ALP, alkaline phosphatase; BUN, blood urea nitrogen; UACR, urine albumin‑to‑creatinine ratio; eGFR, estimated glomerular filtration rate; The Shapiro–Wilk test was used to evaluate the normality of each continuous variable, with a two‑sided p value < 0.05 indicating a statistically significant deviation from the normal distribution. The test was performed on the complete hospital‑based validation cohort (N = 261). Variables with p ≥ 0.05 (specifically, ALB, SCR, and CKMAI) were considered to satisfy the normality assumption, thereby supporting the use of parametric methods for these variables; conversely, the remaining variables exhibited non‑normal distributions (p < 0.05).
