# Supplementary material for: A machine learning-derived aging index for risk stratification and mortality prediction in cardiovascular-kidney-metabolic syndrome: A retrospective cohort study
Source: PLoS Med. 2026 Oct 6;23(10):e1005078. doi: 10.1371/journal.pmed.1005078 (PMC13641365; doi:10.1371/journal.pmed.1005078)
Supplement: Supplementary file 21 [file pmed.1005078.s049.docx]

**Supplementary Table 15.** Association of CKMAI_advance with clinical outcomes in weighted regression analyses

| Outcome | Model | Effect measure | HR / OR (95% CI) | P value |
| --- | --- | --- | --- | --- |
| **All‑cause mortality** | Model 1 | HR | 1.06 (1.04 , 1.07) | <0.001 |
|  | Model 2 | HR | 1.08 (1.07 , 1.10) | <0.001 |
| **Cardiovascular mortality** | Model 1 | HR | 1.04 (1.01 , 1.07) | 0.008 |
|  | Model 2 | HR | 1.06 (1.03 , 1.09) | <0.001 |
| **CKM high‑risk (stage ≥3)** | Model 1 | OR | 1.03 (1.01 , 1.04) | 0.005 |
|  | Model 2 | OR | 1.03 (1.01 , 1.05) | 0.005 |

Abbreviations: HR, hazard ratio; OR, odds ratio; CI, confidence interval; CKMAI, composite kidney–metabolic aging index; CKM, cardiovascular–kidney–metabolic. CKMAI_advance was modeled as a continuous variable (per 1‑unit increment). **Model 1** was adjusted for age and sex. **Model 2** was further adjusted for ethnicity, education level, marital status, poverty‑to‑income ratio, smoking status, alcohol drinking, physical activity, hypertension, diabetes, and cancer, in addition to age and sex. All regression models accounted for the complex multistage probability survey design of NHANES, incorporating primary sampling units (PSUs), stratification, and appropriate mobile examination center (MEC) examination weights to ensure nationally representative estimates
