# Supplementary material for: A machine learning-derived aging index for risk stratification and mortality prediction in cardiovascular-kidney-metabolic syndrome: A retrospective cohort study
Source: PLoS Med. 2026 Oct 6;23(10):e1005078. doi: 10.1371/journal.pmed.1005078 (PMC13641365; doi:10.1371/journal.pmed.1005078)
Supplement: Supplementary file 22 [file pmed.1005078.s050.docx]

**Supplementary Table 16.** Comparison of predictive performance: full 18-variable models vs. CKMAI

| Outcome | Model | Time (years) | AUC (95% CI) |
| --- | --- | --- | --- |
| **All-cause mortality** | 18‑variable Cox model | 3 | 0.889 (0.882,0.892) |
|  |  | 5 | 0.907 (0.900,0.909) |
|  |  | 10 | 0.893 (0.886,0.894) |
|  | **CKMAI** (single index) | 3 | 0.893 (0.857,0.921) |
|  |  | 5 | 0.907 (0.876,0.930) |
|  |  | 10 | 0.890 (0.867,0.910) |
| **CKM high‑risk (stage ≥3)** | 8‑variable stability‑selected model | - | 0.775 (0.770,0.778) |
|  | **CKMAI** (single index) | - | 0.790 (0.765,0.790) |

Abbreviations: AUC, area under the curve; CI, confidence interval. The 18 variable model includes all components of the CKMAI; the 8 variable model includes only variables that passed stability selection for CKM high risk. All AUCs were estimated in the independent test set. 95% CIs were derived from 200 bootstrap resamples. The CKMAI achieved virtually identical performance to the full 18 variable model for mortality, and outperformed the 8 variable stability selected model for CKM high risk, while offering a single, interpretable clinical metric.
