# Supplementary material for: A machine learning-derived aging index for risk stratification and mortality prediction in cardiovascular-kidney-metabolic syndrome: A retrospective cohort study
Source: PLoS Med. 2026 Oct 6;23(10):e1005078. doi: 10.1371/journal.pmed.1005078 (PMC13641365; doi:10.1371/journal.pmed.1005078)
Supplement: Supplementary file 23 [file pmed.1005078.s051.docx]

**Supplementary Table 17.** Reverse mediation analysis: depression score → CKMAI → outcome

| Outcome | Effect | Estimate (Median) | 95% CI | P |
| --- | --- | --- | --- | --- |
| **CKM_high** | ACME | 0.0045 | −0.0175, 0.0046 | >0.05 |
|  | ADE | 0.0595 | 0.0551, 0.0873 | <0.05 |
|  | Prop | 0.0698 | −0.25, 0.0735 | >0.05 |
| **All‑cause mortality** | ACME | −0.0216 | −0.1692, −0.0155 | <0.05 |
|  | ADE | 0.0278 | 0.0207, 0.0508 | <0.05 |
|  | Prop | −1.10 | −8.45, 4.64 | >0.05 |
| **CVD mortality** | ACME | −0.0191 | −0.0262, 0 | >0.05 |
|  | ADE | 0.0111 | −0.004, 0.0253 | >0.05 |
|  | Prop | 2.378 | −0.553, 124.35 | >0.05 |

Abbreviations: ACME, average causal mediation effect (indirect effect); ADE, average direct effect; Prop, proportion mediated; CVD, cardiovascular disease; CI, confidence interval; CKM, cardiovascular--kidney--metabolic; NHANES, National Health and Nutrition Examination Survey; PSU, primary sampling unit; PIR, poverty to income ratio. All effect estimates (ACME and ADE) are presented on the log odds scale for the binary CKM_high outcome and on the log hazard scale for the two mortality outcomes (all cause and CVD mortality). The proportion mediated (Prop) is defined as the ratio of the indirect effect (ACME) to the total effect (ACME + ADE); values can be negative in the presence of competitive mediation or suppression effects, and Prop confidence intervals may extend beyond the [0, 1] range in such cases. All mediation models were adjusted for the following covariates: age, sex, ethnicity, education level, marital status, PIR, smoking status, alcohol drinking, physical activity, hypertension, diabetes, and cancer. The 95% confidence intervals and two sided P values were derived from 500 PSU level cluster bootstrap resamples using the boot package in R, which appropriately account for the complex multistage probability survey design of NHANES (incorporating PSUs, stratification, and mobile examination center examination weights) to ensure nationally representative variance estimation.
