# Supplementary material for: A machine learning-derived aging index for risk stratification and mortality prediction in cardiovascular-kidney-metabolic syndrome: A retrospective cohort study
Source: PLoS Med. 2026 Oct 6;23(10):e1005078. doi: 10.1371/journal.pmed.1005078 (PMC13641365; doi:10.1371/journal.pmed.1005078)
Supplement: Supplementary file 24 [file pmed.1005078.s052.docx]

**Supplementary Table 18.** Moderation analysis: interaction between CKMAI and depression

| Outcome | Interaction term (CKMAI × Depression) | Effect measure | 95% CI | P value |
| --- | --- | --- | --- | --- |
| CKM high‑risk (stage ≥3) | CKMAI × Depression | OR = 0.985 | 0.966,1.004 | 0.116 |
| All‑cause mortality | CKMAI × Depression | HR = 0.977 | 0.953,1.002 | 0.073 |
| Cardiovascular mortality | CKMAI × Depression | HR = 1.007 | 0.971,1.044 | 0.713 |

Abbreviations: CKMAI, composite kidney--metabolic aging index; PIR, poverty to income ratio; PSU, primary sampling unit; OR, odds ratio; HR, hazard ratio; CI, confidence interval. Interaction terms represent the product of CKMAI and depression in the regression models. All models were adjusted for sex, age, ethnicity, education, marital status, PIR, smoking, alcohol drinking, physical activity, hypertension, diabetes, and cancer, and accounted for NHANES complex survey design (PSU, strata, and sampling weights). P values were derived from Wald tests for the interaction term coefficients in the weighted regression models. A P > 0.05 indicates no significant effect modification by depression on the CKMAI--outcome associations.
