# Supplementary material for: A machine learning-derived aging index for risk stratification and mortality prediction in cardiovascular-kidney-metabolic syndrome: A retrospective cohort study
Source: PLoS Med. 2026 Oct 6;23(10):e1005078. doi: 10.1371/journal.pmed.1005078 (PMC13641365; doi:10.1371/journal.pmed.1005078)
Supplement: Supplementary file 25 [file pmed.1005078.s053.docx]

**Supplementary Table 19.** Discriminative ability of CKMAI for CKM high‑risk status (stage ≥ 3)

| Outcome | AUC (95% CI) |
| --- | --- |
| Stage 3 vs 0‑2 | 0.74 (0.72, 0.93) |
| Stage 4 vs 0‑2 | 0.82 (0.80, 0.84) |
| CKM high‑risk (combined) | 0.79 (0.765, 0.790) |

CKMAI, composite kidney–metabolic aging index; CKM, cardiovascular–kidney–metabolic; AUC, area under the receiver operating characteristic curve; CI, confidence interval. AUC estimates and their 95% CIs were calculated using DeLong’s method. In the stage‑specific analyses, the other high‑risk stage was excluded from the reference group (CKM 0–2) to ensure a clean comparison between the two groups.
