# Supplementary material for: A machine learning-derived aging index for risk stratification and mortality prediction in cardiovascular-kidney-metabolic syndrome: A retrospective cohort study
Source: PLoS Med. 2026 Oct 6;23(10):e1005078. doi: 10.1371/journal.pmed.1005078 (PMC13641365; doi:10.1371/journal.pmed.1005078)
Supplement: Supplementary file 26 [file pmed.1005078.s054.docx]

**Supplementary Table 20.** Variance inflation factors for the candidate indices

| Variable | VIF |
| --- | --- |
| PHENOAGE | 3.753 |
| CKMAI | 3.367 |
| CMI | 1.019 |
| KDM | 1.919 |

VIF, variance inflation factor; PHENOAGE, phenotypic age; CKMAI, composite kidney–metabolic aging index; CMI, cardiometabolic index; KDM, Klemera–Doubal method biological age. VIF quantifies the extent of multicollinearity among predictors in a regression model; values below 5 generally indicate no substantial collinearity, while values exceeding 10 suggest problematic multicollinearity.
