# Supplementary material for: A machine learning-derived aging index for risk stratification and mortality prediction in cardiovascular-kidney-metabolic syndrome: A retrospective cohort study
Source: PLoS Med. 2026 Oct 6;23(10):e1005078. doi: 10.1371/journal.pmed.1005078 (PMC13641365; doi:10.1371/journal.pmed.1005078)
Supplement: Supplementary file 27 [file pmed.1005078.s055.docx]

**Supplementary Table 21.** Comparison of baseline characteristics between included and excluded participants (aged ≥18 years)

| Variable | Level | Excluded (n=35,247) | Included (n=6,896) | SMD | Excluded_Missing (%) |
| --- | --- | --- | --- | --- | --- |
| **Demographics** |  |  |  |  |  |
| Age, mean (SD) | – | 47.41 (19.16) | 50.66 (17.41) | 0.177 | 0.0 |
| Sex, n (%) | Male | 16,855 (47.8) | 3,603 (52.2) | 0.089 | 0.0 |
|  | Female | 18,392 (52.2) | 3,293 (47.8) |  | 0.0 |
| Ethnicity, n (%) | Mexican American | 5,677 (16.1) | 1,125 (16.3) | 0.006 | 0.0 |
|  | Non‑Hispanic White | 13,975 (39.6) | 3,219 (46.7) |  |  |
|  | Non‑Hispanic Black | 7,998 (22.7) | 1,255 (18.2) |  |  |
|  | Other | 7,597 (21.6) | 1,297 (18.8) |  |  |
| Education, n (%) | Less than high school | 9,408 (26.7) | 1,565 (22.7) | −0.094 | 0.2 |
|  | High school or equivalent | 8,324 (23.7) | 1,668 (24.2) |  |  |
|  | College or above | 17,452 (49.6) | 3,663 (53.1) |  |  |
| Marital status, n (%) | Married/partner | 19,275 (57.7) | 4,268 (61.9) | 0.085 | 5.2 |
|  | Widowed/divorced/separated | 7,457 (22.3) | 1,551 (22.5) |  |  |
|  | Never married | 6,671 (20.0) | 1,077 (15.6) |  |  |
| PIR, mean (SD) | – | 1.95 (0.79) | 2.04 (0.78) | 0.117 | 11.6 |
| **Lifestyle** |  |  |  |  |  |
| Smoking, n (%) | No | 19,252 (57.1) | 3,624 (52.6) | −0.091 | 4.3 |
|  | Yes | 14,473 (42.9) | 3,272 (47.4) |  |  |
| Drinking, n (%) | No | 9,199 (32.7) | 2,235 (32.4) | −0.006 | 20.2 |
|  | Yes | 18,943 (67.3) | 4,661 (67.6) |  |  |
| Physical activity, n (%) | Low | 13,020 (39.3) | 2,586 (37.5) | −0.036 | 5.9 |
|  | High | 20,137 (60.7) | 4,310 (62.5) |  |  |
| **Anthropometric** |  |  |  |  |  |
| BMI, kg/m², mean (SD) | – | 28.89 (7.01) | 29.47 (6.99) | 0.082 | 6.4 |
| WC, cm, mean (SD) | – | 98.15 (16.55) | 100.44 (16.56) | 0.138 | 11.3 |
| Weight, kg, mean (SD) | – | 80.65 (21.68) | 83.43 (21.93) | 0.127 | 6.2 |
| Height, cm, mean (SD) | – | 166.83 (10.17) | 168.01 (10.03) | 0.117 | 6.2 |
| **Comorbidities** |  |  |  |  |  |
| Hypertension, n (%) | No | 21,421 (60.9) | 3,767 (54.6) | −0.126 | 0.1 |
|  | Yes | 13,776 (39.1) | 3,129 (45.4) |  |  |
| Diabetes, n (%) | No | 28,876 (83.3) | 5,313 (77.0) | −0.158 | 1.7 |
|  | Yes | 5,786 (16.7) | 1,583 (23.0) |  |  |
| Cancer, n (%) | No | 29,714 (90.5) | 6,218 (90.2) | −0.013 | 6.9 |
|  | Yes | 3,104 (9.5) | 678 (9.8) |  |  |
| Depression score, mean (SD) | – | 3.23 (4.30) | 3.08 (4.09) | −0.036 | 16.7 |
| **Laboratory** |  |  |  |  |  |
| WBC, ×10³/μL, mean (SD) | – | 7.38 (3.38) | 6.84 (1.94) | −0.196 | 10.7 |
| LYM, %, mean (SD) | – | 30.69 (8.90) | 30.39 (7.78) | −0.036 | 10.9 |
| MCV, fL, mean (SD) | – | 88.95 (6.14) | 89.19 (4.97) | 0.043 | 10.7 |
| RDW, %, mean (SD) | – | 13.33 (1.43) | 13.21 (1.12) | −0.092 | 10.7 |
| HbA1c, %, mean (SD) | – | 5.72 (1.08) | 5.79 (1.04) | 0.063 | 10.7 |
| Albumin, g/dL, mean (SD) | – | 4.22 (0.37) | 4.20 (0.34) | −0.059 | 12.2 |
| ALP, U/L, mean (SD) | – | 71.03 (26.38) | 71.17 (22.74) | 0.006 | 12.3 |
| BUN, mg/dL, mean (SD) | – | 13.43 (6.12) | 13.96 (5.69) | 0.089 | 12.3 |
| UACR, mg/g, mean (SD) | – | 51.72 (633.81) | 38.87 (250.89) | −0.027 | 7.6 |
| eGFR, mL/min/1.73m², mean (SD) | – | 96.30 (23.85) | 94.47 (21.57) | −0.080 | 12.3 |
| TG, mg/dL, mean (SD) | – | 122.67 (115.28) | 130.16 (96.85) | 0.070 | 68.0 |
| HDL, mg/dL, mean (SD) | – | 53.00 (16.11) | 53.47 (16.00) | 0.029 | 11.9 |
| TC, mg/dL, mean (SD) | – | 191.05 (42.16) | 193.53 (41.89) | 0.059 | 11.9 |
| Platelet, ×10³/μL, mean (SD) | – | 248.36 (66.59) | 246.55 (65.79) | −0.027 | 10.7 |
| FBG, mg/dL, mean (SD) | – | 107.82 (38.18) | 110.99 (32.74) | 0.089 | 67.0 |
| Creatinine, mg/dL, mean (SD) | – | 0.90 (0.47) | 0.89 (0.27) | −0.033 | 12.3 |
| Uric acid, mg/dL, mean (SD) | – | 5.39 (1.45) | 5.61 (1.42) | 0.155 | 12.3 |
| **CKM Staging** |  |  |  |  |  |
| CKM stage, n (%) | 0 | 799 (9.6) | 506 (7.3) | −0.081 | 76.3 |
|  | 1 | 1,441 (17.3) | 1,118 (16.2) |  |  |
|  | 2 | 4,633 (55.5) | 4,105 (59.5) |  |  |
|  | 3 | 479 (5.7) | 374 (5.4) |  |  |
|  | 4 | 992 (11.9) | 793 (11.5) |  |  |
| **Outcomes** |  |  |  |  |  |
| All‑cause mortality, n (%) | No | 31,256 (89.0) | 6,236 (90.4) | 0.048 | 0.3 |
|  | Yes | 3,870 (11.0) | 660 (9.6) |  |  |
| CVD mortality, n (%) | No | 33,959 (96.7) | 6,695 (97.1) | 0.023 | 0.3 |
|  | Yes | 1,167 (3.3) | 201 (2.9) |  |  |

SMD, standardized mean difference; PIR, poverty‑to‑income ratio; CKM, cardiovascular–kidney–metabolic; WBC, white blood cell count; LYM, lymphocyte percentage; MCV, mean corpuscular volume; RDW, red cell distribution width; HbA1c, glycated hemoglobin; ALP, alkaline phosphatase; BUN, blood urea nitrogen; UACR, urine albumin‑to‑creatinine ratio; eGFR, estimated glomerular filtration rate; TG, triglycerides; HDL, high‑density lipoprotein; TC, total cholesterol; FBG, fasting blood glucose; CVD, cardiovascular disease. Data are presented as mean (SD) for continuous variables and n (%) for categorical variables. SMD quantifies the balance between excluded and included participants; values < 0.2 generally indicate good balance. Excluded_Missing denotes the proportion of missing data in the excluded sample prior to exclusion.

.
