# Supplementary material for: A machine learning-derived aging index for risk stratification and mortality prediction in cardiovascular-kidney-metabolic syndrome: A retrospective cohort study
Source: PLoS Med. 2026 Oct 6;23(10):e1005078. doi: 10.1371/journal.pmed.1005078 (PMC13641365; doi:10.1371/journal.pmed.1005078)
Supplement: Supplementary file 28 [file pmed.1005078.s056.docx]

**Supplementary Table 22**. Performance of CKMAI, KDM, CMI, and PhenoAge for predicting all‑cause mortality, CVD mortality, and CKM high-risk status across 20 imputed datasets

| Dataset | Index | Cindex_all | Cindex_all_95CI | Cindex_cvd | Cindex_cvd_95CI | AUC_ckm | AUC_ckm_95CI |
| --- | --- | --- | --- | --- | --- | --- | --- |
| 1 | CKMAI | 0.851 | 0.846,0.856 | 0.872 | 0.864,0.88 | 0.787 | 0.781,0.792 |
| 1 | KDM | 0.79 | 0.783,0.797 | 0.825 | 0.816,0.836 | 0.765 | 0.759,0.771 |
| 1 | CMI | 0.531 | 0.523,0.541 | 0.563 | 0.547,0.579 | 0.628 | 0.621,0.635 |
| 1 | PhenoAge | 0.807 | 0.8,0.814 | 0.829 | 0.815,0.839 | 0.756 | 0.75,0.762 |
| 2 | CKMAI | 0.85 | 0.845,0.855 | 0.873 | 0.863,0.882 | 0.787 | 0.781,0.793 |
| 2 | KDM | 0.787 | 0.781,0.795 | 0.826 | 0.816,0.837 | 0.766 | 0.76,0.772 |
| 2 | CMI | 0.527 | 0.517,0.536 | 0.552 | 0.537,0.568 | 0.621 | 0.613,0.628 |
| 2 | PhenoAge | 0.808 | 0.802,0.815 | 0.833 | 0.823,0.843 | 0.753 | 0.747,0.759 |
| 3 | CKMAI | 0.85 | 0.844,0.856 | 0.872 | 0.861,0.881 | 0.789 | 0.783,0.795 |
| 3 | KDM | 0.786 | 0.778,0.793 | 0.823 | 0.812,0.833 | 0.766 | 0.76,0.772 |
| 3 | CMI | 0.532 | 0.522,0.541 | 0.565 | 0.55,0.582 | 0.624 | 0.617,0.632 |
| 3 | PhenoAge | 0.808 | 0.801,0.814 | 0.833 | 0.823,0.845 | 0.758 | 0.752,0.764 |
| 4 | CKMAI | 0.851 | 0.846,0.856 | 0.873 | 0.863,0.88 | 0.783 | 0.777,0.789 |
| 4 | KDM | 0.787 | 0.78,0.793 | 0.823 | 0.811,0.834 | 0.763 | 0.757,0.769 |
| 4 | CMI | 0.537 | 0.528,0.546 | 0.558 | 0.542,0.574 | 0.628 | 0.621,0.635 |
| 4 | PhenoAge | 0.81 | 0.804,0.817 | 0.833 | 0.821,0.844 | 0.754 | 0.748,0.76 |
| 5 | CKMAI | 0.852 | 0.846,0.858 | 0.874 | 0.864,0.882 | 0.787 | 0.781,0.792 |
| 5 | KDM | 0.788 | 0.78,0.795 | 0.824 | 0.815,0.835 | 0.766 | 0.76,0.772 |
| 5 | CMI | 0.533 | 0.525,0.542 | 0.552 | 0.534,0.567 | 0.627 | 0.62,0.634 |
| 5 | PhenoAge | 0.808 | 0.801,0.815 | 0.831 | 0.82,0.842 | 0.757 | 0.751,0.763 |
| 6 | CKMAI | 0.85 | 0.845,0.856 | 0.871 | 0.863,0.881 | 0.789 | 0.783,0.794 |
| 6 | KDM | 0.788 | 0.781,0.796 | 0.822 | 0.81,0.832 | 0.769 | 0.763,0.775 |
| 6 | CMI | 0.536 | 0.527,0.545 | 0.559 | 0.545,0.575 | 0.634 | 0.627,0.641 |
| 6 | PhenoAge | 0.807 | 0.801,0.814 | 0.832 | 0.822,0.842 | 0.758 | 0.752,0.764 |
| 7 | CKMAI | 0.851 | 0.845,0.857 | 0.875 | 0.867,0.883 | 0.788 | 0.783,0.794 |
| 7 | KDM | 0.79 | 0.782,0.797 | 0.825 | 0.812,0.835 | 0.769 | 0.763,0.775 |
| 7 | CMI | 0.533 | 0.524,0.542 | 0.552 | 0.536,0.564 | 0.627 | 0.62,0.634 |
| 7 | PhenoAge | 0.808 | 0.802,0.814 | 0.835 | 0.824,0.845 | 0.76 | 0.754,0.766 |
| 8 | CKMAI | 0.851 | 0.845,0.857 | 0.873 | 0.864,0.881 | 0.789 | 0.783,0.794 |
| 8 | KDM | 0.787 | 0.779,0.794 | 0.825 | 0.813,0.835 | 0.768 | 0.762,0.774 |
| 8 | CMI | 0.526 | 0.517,0.535 | 0.551 | 0.537,0.568 | 0.625 | 0.618,0.632 |
| 8 | PhenoAge | 0.809 | 0.802,0.816 | 0.834 | 0.822,0.844 | 0.757 | 0.751,0.763 |
| 9 | CKMAI | 0.85 | 0.844,0.856 | 0.872 | 0.863,0.88 | 0.799 | 0.794,0.805 |
| 9 | KDM | 0.789 | 0.783,0.795 | 0.824 | 0.812,0.835 | 0.776 | 0.77,0.781 |
| 9 | CMI | 0.523 | 0.514,0.534 | 0.532 | 0.479,0.548 | 0.596 | 0.588,0.604 |
| 9 | PhenoAge | 0.809 | 0.803,0.815 | 0.834 | 0.824,0.844 | 0.768 | 0.762,0.774 |
| 10 | CKMAI | 0.853 | 0.847,0.857 | 0.875 | 0.866,0.883 | 0.788 | 0.782,0.794 |
| 10 | KDM | 0.77 | 0.763,0.777 | 0.801 | 0.791,0.812 | 0.732 | 0.726,0.738 |
| 10 | CMI | 0.531 | 0.523,0.539 | 0.545 | 0.529,0.563 | 0.629 | 0.621,0.636 |
| 10 | PhenoAge | 0.808 | 0.802,0.814 | 0.833 | 0.825,0.842 | 0.76 | 0.754,0.766 |
| 11 | CKMAI | 0.85 | 0.845,0.855 | 0.87 | 0.861,0.879 | 0.789 | 0.783,0.795 |
| 11 | KDM | 0.789 | 0.782,0.796 | 0.824 | 0.814,0.835 | 0.766 | 0.76,0.772 |
| 11 | CMI | 0.531 | 0.52,0.541 | 0.565 | 0.549,0.58 | 0.627 | 0.619,0.634 |
| 11 | PhenoAge | 0.811 | 0.805,0.818 | 0.84 | 0.826,0.849 | 0.758 | 0.752,0.764 |
| 12 | CKMAI | 0.851 | 0.846,0.856 | 0.873 | 0.865,0.882 | 0.786 | 0.78,0.791 |
| 12 | KDM | 0.788 | 0.782,0.796 | 0.823 | 0.813,0.832 | 0.765 | 0.759,0.771 |
| 12 | CMI | 0.529 | 0.519,0.538 | 0.551 | 0.532,0.566 | 0.621 | 0.614,0.628 |
| 12 | PhenoAge | 0.808 | 0.803,0.815 | 0.834 | 0.823,0.845 | 0.753 | 0.747,0.759 |
| 13 | CKMAI | 0.85 | 0.845,0.856 | 0.873 | 0.864,0.88 | 0.788 | 0.782,0.794 |
| 13 | KDM | 0.789 | 0.783,0.795 | 0.826 | 0.814,0.839 | 0.767 | 0.761,0.773 |
| 13 | CMI | 0.525 | 0.513,0.535 | 0.548 | 0.531,0.565 | 0.627 | 0.62,0.634 |
| 13 | PhenoAge | 0.808 | 0.801,0.815 | 0.83 | 0.821,0.843 | 0.756 | 0.75,0.762 |
| 14 | CKMAI | 0.85 | 0.845,0.855 | 0.868 | 0.86,0.877 | 0.789 | 0.784,0.795 |
| 14 | KDM | 0.79 | 0.782,0.796 | 0.82 | 0.81,0.831 | 0.768 | 0.762,0.774 |
| 14 | CMI | 0.529 | 0.52,0.538 | 0.552 | 0.537,0.568 | 0.624 | 0.617,0.631 |
| 14 | PhenoAge | 0.809 | 0.802,0.814 | 0.832 | 0.821,0.842 | 0.757 | 0.751,0.763 |
| 15 | CKMAI | 0.852 | 0.845,0.857 | 0.876 | 0.868,0.884 | 0.787 | 0.782,0.793 |
| 15 | KDM | 0.788 | 0.781,0.795 | 0.825 | 0.813,0.836 | 0.768 | 0.762,0.774 |
| 15 | CMI | 0.531 | 0.521,0.542 | 0.557 | 0.539,0.574 | 0.627 | 0.62,0.634 |
| 15 | PhenoAge | 0.806 | 0.8,0.812 | 0.832 | 0.823,0.842 | 0.755 | 0.749,0.762 |
| 16 | CKMAI | 0.851 | 0.847,0.857 | 0.871 | 0.863,0.88 | 0.786 | 0.78,0.791 |
| 16 | KDM | 0.79 | 0.784,0.797 | 0.824 | 0.814,0.835 | 0.764 | 0.758,0.77 |
| 16 | CMI | 0.532 | 0.523,0.543 | 0.552 | 0.536,0.569 | 0.63 | 0.623,0.638 |
| 16 | PhenoAge | 0.808 | 0.8,0.814 | 0.834 | 0.823,0.844 | 0.754 | 0.748,0.76 |
| 17 | CKMAI | 0.85 | 0.845,0.857 | 0.873 | 0.864,0.881 | 0.788 | 0.782,0.793 |
| 17 | KDM | 0.789 | 0.781,0.796 | 0.824 | 0.814,0.835 | 0.766 | 0.76,0.772 |
| 17 | CMI | 0.533 | 0.523,0.541 | 0.56 | 0.545,0.575 | 0.624 | 0.617,0.631 |
| 17 | PhenoAge | 0.808 | 0.802,0.814 | 0.832 | 0.82,0.841 | 0.759 | 0.753,0.765 |
| 18 | CKMAI | 0.851 | 0.846,0.857 | 0.873 | 0.865,0.881 | 0.786 | 0.78,0.791 |
| 18 | KDM | 0.787 | 0.78,0.794 | 0.826 | 0.816,0.835 | 0.766 | 0.76,0.772 |
| 18 | CMI | 0.535 | 0.526,0.544 | 0.551 | 0.535,0.567 | 0.625 | 0.618,0.632 |
| 18 | PhenoAge | 0.808 | 0.802,0.815 | 0.837 | 0.824,0.847 | 0.754 | 0.748,0.76 |
| 19 | CKMAI | 0.851 | 0.845,0.856 | 0.874 | 0.865,0.883 | 0.764 | 0.756,0.772 |
| 19 | KDM | 0.787 | 0.781,0.794 | 0.823 | 0.815,0.835 | 0.727 | 0.718,0.736 |
| 19 | CMI | 0.525 | 0.516,0.534 | 0.541 | 0.524,0.555 | 0.597 | 0.587,0.608 |
| 19 | PhenoAge | 0.808 | 0.802,0.814 | 0.832 | 0.821,0.841 | 0.742 | 0.733,0.751 |
| 20 | CKMAI | 0.852 | 0.845,0.857 | 0.874 | 0.865,0.883 | 0.789 | 0.783,0.794 |
| 20 | KDM | 0.788 | 0.781,0.795 | 0.823 | 0.811,0.834 | 0.764 | 0.758,0.77 |
| 20 | CMI | 0.537 | 0.528,0.546 | 0.562 | 0.548,0.576 | 0.628 | 0.621,0.635 |
| 20 | PhenoAge | 0.809 | 0.802,0.815 | 0.831 | 0.821,0.84 | 0.758 | 0.752,0.764 |

CKMAI, composite kidney--metabolic aging index; KDM, Klemera--Doubal method biological age; CMI, cardiometabolic index; PHENOAGE, phenotypic age; C index, Harrell's concordance index; AUC, area under the receiver operating characteristic curve; CI, confidence interval; CKM, cardiovascular--kidney--metabolic. Multiple imputation was performed using the MICE package (20 imputations) with chained equations. The C index was used to assess discrimination for all cause mortality (Cindex_all) and cardiovascular mortality (Cindex_cvd) as time to event outcomes, whereas AUC was applied to the binary CKM high risk status. 95% CIs were derived from 200 bootstrap resamples within each imputed dataset.
