# Supplementary material for: A machine learning-derived aging index for risk stratification and mortality prediction in cardiovascular-kidney-metabolic syndrome: A retrospective cohort study
Source: PLoS Med. 2026 Oct 6;23(10):e1005078. doi: 10.1371/journal.pmed.1005078 (PMC13641365; doi:10.1371/journal.pmed.1005078)
Supplement: Supplementary file 29 [file pmed.1005078.s057.docx]

**Supplementary Table 23.** Summary of performance across 20 imputed datasets

| **Index** | **C‑index (all‑cause)** | **C‑index (CVD)** | **AUC (CKM≥3)** |
| --- | --- | --- | --- |
| CKMAI | 0.851 (0.845,0.857) | 0.873 (0.863,0.882) | 0.787 (0.773,0.801) |
| KDM | 0.787 (0.776,0.799) | 0.823 (0.807,0.838) | 0.763 (0.737,0.789) |
| CMI | 0.531 (0.518,0.543) | 0.553 (0.529,0.578) | 0.623 (0.602,0.645) |
| PhenoAge | 0.808 (0.801,0.815) | 0.833 (0.821,0.845) | 0.756 (0.745,0.768) |
| KDM | 0.787 (0.776,0.799) | 0.823 (0.807,0.838) | 0.763 (0.737,0.789) |

CKMAI, composite kidney--metabolic aging index; KDM, Klemera--Doubal method biological age; CMI, cardiometabolic index; PhenoAge, phenotypic age; C index, Harrell's concordance index (for time to event outcomes); AUC, area under the receiver operating characteristic curve (for binary outcome); CKM, cardiovascular--kidney--metabolic; CI, confidence interval. All performance metrics were calculated on the independent test set for each of the 20 multiply imputed datasets. Point estimates and 95% CIs were pooled across imputations using Rubin's rules, with variances combined according to the within and between imputation variability. 95% CIs for individual imputations were derived from 200 bootstrap resamples. The C index was used for mortality outcomes (all cause and CVD), while AUC was used for CKM high risk status (stage ≥ 3).
