# Supplementary material for: A machine learning-derived aging index for risk stratification and mortality prediction in cardiovascular-kidney-metabolic syndrome: A retrospective cohort study
Source: PLoS Med. 2026 Oct 6;23(10):e1005078. doi: 10.1371/journal.pmed.1005078 (PMC13641365; doi:10.1371/journal.pmed.1005078)
Supplement: Supplementary file 30 [file pmed.1005078.s058.docx]

**Supplementary Table 24.** Sensitivity analysis for cardiovascular mortality: comparison of Cox and Fine Gray models

| **Model** | **Effect measure** | **HR / SHR (95% CI)** | **P value** |
| --- | --- | --- | --- |
| Primary Cox (weighted, cause‑specific) | HR | 1.06 (1.03, 1.09) | <0.001 |
| Weighted cause‑specific Cox (replication) | HR | 1.046 (1.013, 1.081) | 0.007 |
| Weighted Fine‑Gray (competing risk) | SHR | 1.024 (1.004 ,1.045) | 0.0178 |

HR, hazard ratio; SHR, subdistribution hazard ratio; CI, confidence interval; CVD, cardiovascular disease; CKMAI, composite kidney--metabolic aging index; NHANES, National Health and Nutrition Examination Survey; PSU, primary sampling unit. The primary Cox model was a weighted cause specific Cox regression accounting for the NHANES complex survey design (PSU, strata, and sampling weights). The replication model used identical specifications to confirm the robustness of the primary estimate. The Fine Gray competing risk model treated non CVD deaths as competing events and reported subdistribution hazard ratios (SHRs) to account for the cumulative incidence of cardiovascular mortality. All models were adjusted for age, sex, ethnicity, education, marital status, poverty to income ratio, smoking, alcohol drinking, physical activity, hypertension, diabetes, and cancer. P values were derived from Wald tests for the Cox models and from the Fine Gray model test for the SHR.
