# Supplementary material for: A machine learning-derived aging index for risk stratification and mortality prediction in cardiovascular-kidney-metabolic syndrome: A retrospective cohort study
Source: PLoS Med. 2026 Oct 6;23(10):e1005078. doi: 10.1371/journal.pmed.1005078 (PMC13641365; doi:10.1371/journal.pmed.1005078)
Supplement: Supplementary file 31 [file pmed.1005078.s059.docx]

**Supplementary Table 25.** Subgroup performance of the CKMAI across sex and race/ethnicity strata

| **Stratification** | **Subgroup** | **n** | **events_all** | **events_cvd** | **events_ckm** | **C-index_all (95%CI)** | **C-index_cvd (95%CI)** | **AUC3_all (95%CI)** | **AUC5_all (95%CI)** | **AUC10_all (95%CI)** | **AUC3_cvd (95%CI)** | **AUC5_cvd (95%CI)** | **AUC10_cvd (95%CI)** | **AUC_ckm (95%CI)** |
| --- | --- | --- | --- | --- | --- | --- | --- | --- | --- | --- | --- | --- | --- | --- |
| Sex | Male | 3603 | 384 | 118 | 789 | 0.835(0.816,0.855) | 0.845(0.820,0.874) | 0.842(0.806,0.877) | 0.867(0.840,0.896) | 0.864(0.847,0.886) | 0.862(0.808,0.886) | 0.892(0.860,0.910) | 0.871(0.837,0.904) | 0.762(0.746,0.779) |
| Sex | Female | 3293 | 276 | 83 | 378 | 0.842(0.820,0.864) | 0.870(0.827,0.914) | 0.858(0.819,0.901) | 0.846(0.801,0.888) | 0.858(0.839,0.884) | 0.855(0.743,0.940) | 0.877(0.790,0.935) | 0.892(0.855,0.933) | 0.727(0.693,0.750) |
| Ethnicity | Non-Hispanic White | 3219 | 449 | 145 | 641 | 0.828(0.810,0.845) | 0.852(0.825,0.882) | 0.838(0.802,0.873) | 0.861(0.834,0.887) | 0.864(0.842,0.883) | 0.849(0.799,0.896) | 0.885(0.848,0.922) | 0.891(0.865,0.914) | 0.762(0.742,0.784) |
| Ethnicity | Other | 1297 | 50 | 13 | 169 | 0.902(0.865,0.931) | 0.930(0.876,0.970) | 0.920(0.878,0.957) | 0.921(0.848,0.972) | 0.917(0.879,0.950) | 0.898(0.754,0.993) | 0.959(0.906,1.000) | 0.938(0.893,0.975) | 0.749(0.706,0.783) |
| Ethnicity | Mexican American | 1125 | 52 | 12 | 123 | 0.824(0.769,0.879) | 0.740(0.546,0.890) | 0.827(0.752,0.896) | 0.893(0.842,0.938) | 0.841(0.761,0.908) | 0.795(0.525,0.987) | 0.878(0.696,0.979) | 0.717(0.459,0.938) | 0.767(0.721,0.811) |
| Ethnicity | Non-Hispanic Black | 1255 | 109 | 31 | 234 | 0.794(0.754,0.841) | 0.797(0.727,0.866) | 0.847(0.770,0.923) | 0.781(0.708,0.857) | 0.803(0.759,0.854) | 0.828(0.707,0.971) | 0.820(0.702,0.910) | 0.819(0.752,0.889) | 0.730(0.697,0.760) |

Abbreviations: AUC, area under the time dependent receiver operating characteristic curve; CI, confidence interval; CKM, cardiovascular kidney metabolic; CVD, cardiovascular disease; events_all, all cause deaths; events_cvd, cardiovascular deaths; events_ckm, participants with CKM stage ≥3. All performance metrics are based on the CKMAI as a continuous predictor, evaluated in the full analytical cohort (n=6,896). Harrell's C index for all cause and CVD mortality, and time dependent AUC at 3, 5, and 10 years for both mortality outcomes, were calculated using timeROC. AUC for high risk CKM status (stage ≥3) was calculated using logistic ROC. 95% confidence intervals were derived from 200 bootstrap resamples using the boot package in R. "Other" ethnicity includes individuals not classified as Non Hispanic White, Non Hispanic Black, or Mexican American.
